# Oxindole–benzothiazole hybrids as CDK2 inhibitors and anticancer agents: design, synthesis and biological evaluation

**DOI:** 10.1186/s13065-024-01277-1

**Published:** 2024-09-13

**Authors:** Heba T. Abdel-Mohsen

**Affiliations:** Chemistry of Natural and Microbial Products Department, Pharmaceutical and Drug Industries Research Institute, National Research Centre, Dokki, P.O. 12622, Cairo, Egypt

**Keywords:** Design, Synthesis, Oxindole–benzothiazole, CDK2 inhibitory activity, Anticancer activity, Molecular docking

## Abstract

In the current study, molecular hybridization between the oxindole core and benzothiazole system through an acetohydrazide moiety was accomplished for the design of a new series of oxindole–benzothiazole hybrids **9a**–**r** targeting CDK2 for cancer therapy. The afforded hybrids displayed promising growth inhibitory activity on NCI cancer cell lines at 10 µM. Compound **9o** displayed mean GI% = 55.91%. Based on the potent activity of **9o**, it was further assessed for its cytotoxic activity at five dose level and it demonstrated GI_50_ reaching 2.02 µM. Analysis of the cell cycle of the prostate cancer cell line DU145 after treatment with **9o** confirmed its ability to arrest its cell cycle at the G1 phase. Moreover, **9o** proved its ability to potentiate the apoptosis and necrosis of the same cell line. Furthermore, the oxindole–benzothiazole hybrids **9b**, **9f** and **9o** showed IC_50_ = 0.70, 0.20 and 0.21 µM, respectively on CDK2. Besides, molecular docking simulation of the synthesized oxindole–benzothiazole hybrid **9o** proved the expected binding mode which involves the accommodation of the oxindole moiety in the ATP binding pocket where it is involved in hydrogen bonding and hydrophobic interactions with the essential amino acids in the hinge region while the benzothiazole moiety is oriented toward the solvent region. Investigation of the physicochemical properties of the hybrids **9a**–**r** highlights their acceptable ADME properties that can be somewhat developed for the discovery of new anticancer agents.

**Supplementary Information:**

The online version contains supplementary material available at 10.1186/s13065-024-01277-1.

## Introduction

Cancer is a global critical heterogeneous disease that arises as a result of unlimited proliferation of cells [1]. Prescription of traditional chemotherapeutic agents was one of the main approaches for the treatment of cancer [2]. However, it is always associated with unselectivity, severe side effects and toxicity. One approach to counteract this drawback is the prescription of a targeted therapy that targets a pathway that is overexpressed in cancer and plays a key role in controlling the proliferation of cancer cells without affecting the normal cells [3]. One of these targeted therapies is the protein kinase inhibitors [3–9].

Cyclin-dependent kinases (CDKs) are a class of serine/threonine kinases that participate directly in the regulation of the cell cycle besides their role in the regulation of growth, proliferation and apoptosis [10]. CDK2 is a subtype from the CDK family that plays a major role in the mechanism of the cell cycle. Several studies reported the up-regulation of CDK2 in diverse types of cancer including breast cancer, prostate cancer, liver cancer and lung cancer [11]. Hence, targeting CDK2 is considered a promising approach for controlling the progression of cancer [12].

**Fig. 1:**
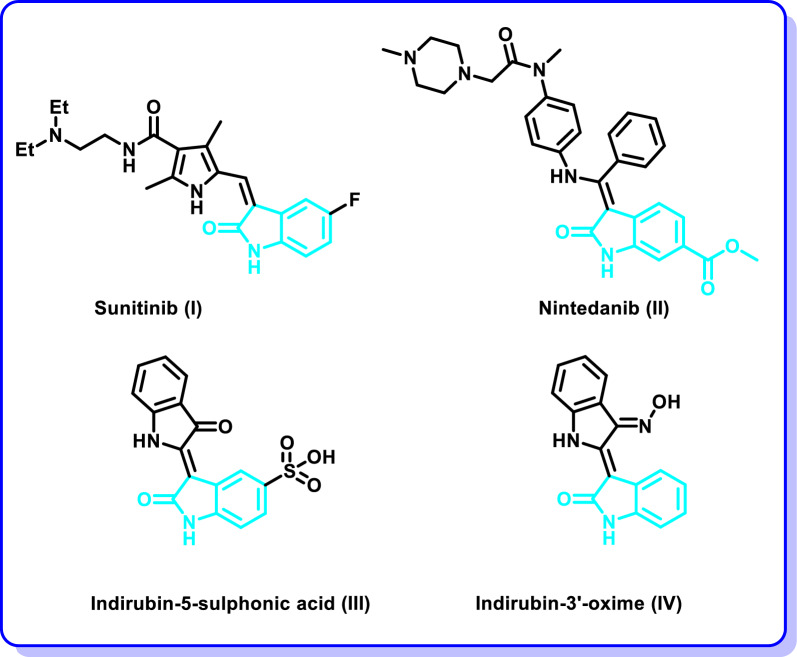
Examples of oxindole based protein kinase inhibitors **I**–**IV**

1*H*-indol-2,3-dione (isatin) is an alkaloid of natural origin that was extracted from the plants of *Isatis genus* [13]. Isatin displayed diverse medicinal applications as an anti-inflammatory and chemotherapeutic agent. Hence, it was extensively utilized as a precursor for the development of different chemotherapeutic agents and protein kinase inhibitors [14–17]. Sunitinib (**I**) (Fig. 1) is an example of isatin incorporating multi-protein kinase inhibitor (VEGFR-2/3, PDGFRα/β, CHK2, and cKit) that was licensed by FDA in the treatment regimen for patients suffering from renal cell carcinomas as well as some types pancreatic tumors [18]. Moreover, Nintedanib (**II**) (Fig. 1) is a multi-angiokinase inhibitor (VEGFR1/2/3, FGFR1/2/3 and PDGFRα/β) that was licensed recently by FDA as an adjuvant therapy for cases suffering from idiopathic pulmonary fibrosis or certain types non-small cell cancer [19, 20]. Also, indurubins are oxindole derivatives with promising antiproliferative activity as well as protein kinase inhibitory activity [21, 22]. For example, indirubin-5-sulphonic acid (**III**) (Fig. 1) displayed potent CDK2 inhibitory activity with IC_50_ = 35 nM) [23]. In addition, indirubin-3′-oxime (**IV**) displayed potent CDK2 inhibitory activity with IC_50_ = 440 nM. In addition, it revealed a broad spectrum of anticancer activity and it arrests the cell cycle at G2/M phase [24].

**Fig. 2:**
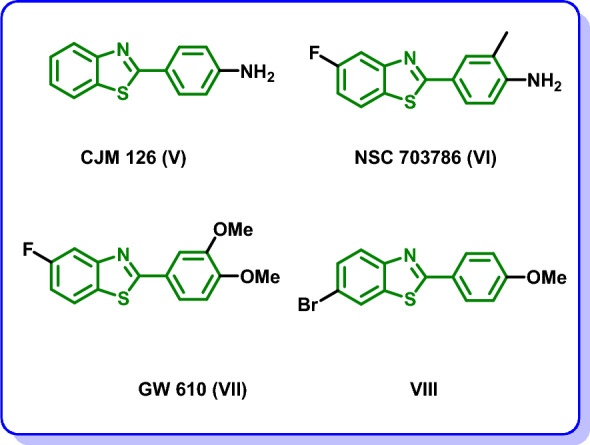
Structures of anticancer benzothiazoles **V**–**VIII**

On the other side, 2-aryl benzothiazole is a privileged scaffold that was reported in diverse molecules with promising anticancer activity and protein kinase inhibitory activity. CJM 126 (**V**) and NSC 703786 (**VI**) (Fig. 2) were reported to induce DNA damage in diverse cancer cell lines including breast, ovarian and colon cancer cell lines [25, 26]. Moreover, GW 610 (**VII**) (Fig. 2) revealed sub-nanomolar growth inhibitory activity in vitro against breast cancer [27]. In addition, compound **VIII** (Fig. 2) was reported to exhibit high growth inhibitory potency of MCF-7 cell line [28, 29].

**Fig. 3:**
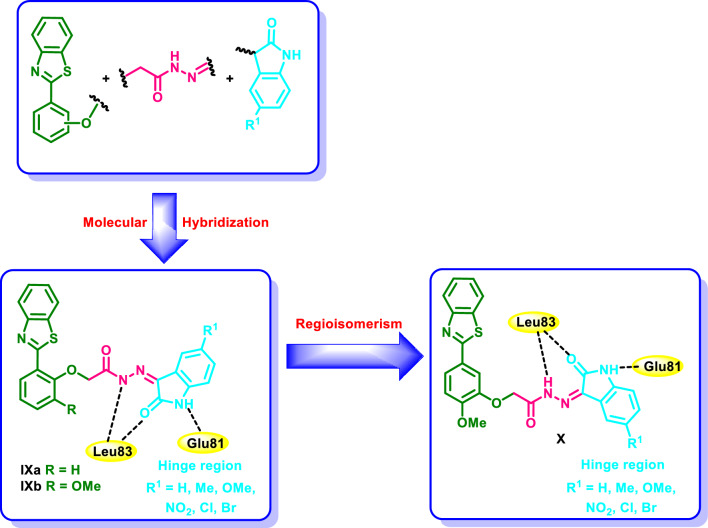
Proposed strategy for the design of the oxindole–benzothaizole hybrids **IX** and** X** as CDK2 inhibitors

Encouraged by the potent antiproliferative activity in conjunction with the privileged protein kinase inhibitory activity of both the oxindole and benzothiazole moieties we were curious in the current investigation to design a new scaffold of oxindole–benzothiazole hybrids **IX** and **X** (Fig. 3) as CDK2 inhibitors. The designed oxindole–benzothiazole scaffold **IX** and **X** was tailored so that the oxindole moiety was linked to 2-phenyl benzothiazole moiety through acetohydrazide linker. The oxindole moiety is expected to occupy the ATP binding site of CDK2 and perform hydrogen bonding with the key amino acid residues Glu81 and Leu83 through CONH group. The oxindole moiety is further settled in the ATP binding site by the ability of the fused benzene ring to form hydrophobic interactions with the side chains of the amino acids lining this region. The benzothiazole moiety is directed towards the solvent region. For studying the SAR, initially scaffold **IXa** (Fig. 3) was designed followed by the introduction of a methoxy group at the three position in **IXb** (Fig. 3) followed by regioisomersim of the oxindole moiety from the two position in scaffolds **IXa** and **IXb** (Fig. 3) to the three position in **X** (Fig. 3). The oxindole–benzothiazole scaffold was subsequently synthesized and submitted for screening their cytotoxic activity on different NCI cell lines derived from diverse types of cancer. The most potent hybrids were subsequently evaluated for their effect on the cell cycle and the apoptosis of a selected cell line. Additionally, the most potent candidate was docked into the binding site of CDK2 to confirm the design strategy.

## Results and discussion

### Chemistry

**Fig. 4:**
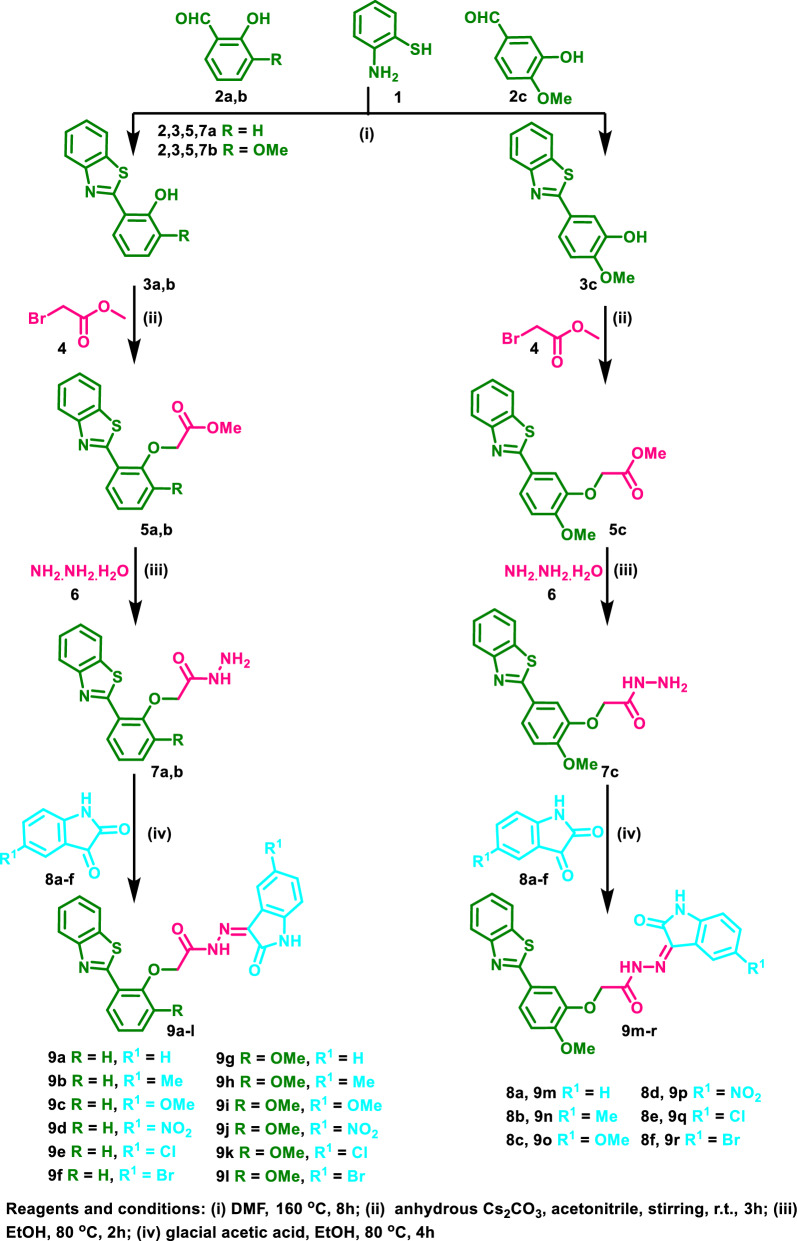
Synthesis of oxindole–benzothiazole hybrids **9a**–**r**

The designed oxindole–benzothiazole hybrids **9a**–**r** was synthesized according to the pathway depicted in Fig. 4. Initially, *o*-aminothiophenol (**1**) was reacted with salicylaldehyde (**2a**), *o*-vanillin (**2b**) or isovanillin (**2c**) in DMF under reflux to afford the corresponding 2-substituted benzothiazole derivatives **3a**–**c** [30, 31]. The hydroxy moiety of **3a**–**c** was further functionalized by the base-catalyzed reaction of **3a**–**c** with methyl bromoacetate (**4**) at room temperature to afford **5a**–**c** which were further reacted with excess hydrazine hydrate (**6**) under reflux to yield the corresponding acid hydrazides **7a**–**c** [30, 32]. The benzothiazole acetohydrazides **7a**–**c** were further reacted with diverse oxindoles **8a**–**f** under acidic conditions to afford the target derivatives **9a**–**r** in good yields (Fig. 4). The structures of the afforded derivatives were further confirmed by IR, ^1^H NMR and ^13^C NMR spectra (for further details see Additional file 1: NMR Spectra of oxindole–benzothiazole hybrids **9a**–**r**; IR charts of the synthesized oxindole–benzothiazoles). For instance, the IR spectrum of **9a** showed the appearance of two bands at *ṽ* 3221 and 3148 cm^−1^ corresponding to NH groups; two bands at *ṽ* 3059 and 3036 cm^−1^ corresponding to aromatic CH; a band at *ṽ* 2959 cm^−1^ corresponding to aliphatic CH; two bands at *ṽ* 1721 and 1694 cm^−1^ corresponding to CO. ^1^H NMR spectrum of **9a** showed the appearance of two singlets at *δ*_H_ 5.25 and 5.64 ppm each corresponding to one proton of the CH_2_ group; one singlet at *δ*_H_ 6.92 corresponding to one aromatic proton; two triplets at *δ*_H_ 7.09 and 7.22 ppm each corresponding to one aromatic proton; a doublet at *δ*_H_ 7.32 ppm corresponding to one aromatic proton; three triplets at *δ*_H_ 7.38, 7.43 and 7.54 ppm corresponding to one, one and two aromatic protons, respectively; three doublets at *δ*_H_ 7.58, 8.07 and 8.12 ppm each corresponding to one aromatic proton; a doublet of doublet at *δ*_H_ 8.47 ppm corresponding to one aromatic proton and two broad peaks at *δ*_H_ 11.23 and 13.52 ppm each corresponding to one NH group. ^13^C NMR spectrum displayed the appearance of a signal at *δ*_C_ 68.50 ppm corresponding to CH_2_; signals at *δ*_C_ 111.26, 113.71, 113.99, 119.63, 121.09, 121.86, 122.55, 122.72, 125.05, 126.32, 129.03, 132.01, 132.33, 135.64, 142.68, 151.62, 155.61, 162.48 ppm corresponding to aromatic carbons and CO groups.

### Biological evaluation

#### Screening of the antiproliferative activity on NCI cancer cell lines at single dose concentration

The oxindole–benzothiazole conjugates **9a**–**c** and **9e**–**r** were assayed for their potential to inhibit the growth of cancer cell lines that originate from diverse types of cancer after treatment with 10 µM concentrations at NCI-USA and the results were depicted in Table 1 and compared with milciclib as a standard (Additional file 1: screening of cytotoxic activity against a panel of sixty human tumor cell lines; one dose mean graphs of the oxindole–benzothiazoles).

**Table 1:** In vitro growth inhibition% (GI%) of NCI 60 cancer cell line panel after treatment with 10 μM of the oxindole–benzothiazoles **9a**–**c and 9e-r**

Cell name	GI%																	
	**9a**	**9b**	**9c**	**9e**	**9f**	**9g**	**9h**	**9i**	**9j**	**9k**	**9l**	**9m**	**9n**	**9o**	**9p**	**9q**	**9r**	**Milciclib**
Leukemia																		
CCRF-CEM	20.22	23.64	60.17	–^a^	21.91	41.07	47.56	44.24	34.81	24.47	15.16	40.05	16.11	57.64	9.28	81.35	42.87	89.60
HL-60(TB)	–	8.38	15.59	–	29.39	15.30	–	–	–	6.09	–	–	–	7.99	–	–	–	127.0
K-562	–	20.57	30.54	–	22.52	32.23	26.17	21.95	–	29.20	18.70	11.23	6.70	64.36	–	50.00	24.52	94.6
MOLT-4	8.23	29.12	47.19	–	36.74	49.06	43.65	45.30	9.01	43.70	36.40	23.70	13.07	36.32	–	71.36	13.12	97.70
RPMI-8226	21.80	30.53	48.87	–	29.01	50.80	54.14	47.68	22.11	29.96	25.41	25.46	14.26	66.93	–	46.48	19.17	99.60
SR	–	18.71	20.98	6.46	43.03	18.95	–	–	–	17.83	16.68	12.20	nd^b^	35.47	–	18.57	–	101.8
Non-small cell lung cancer																		
A549/ATCC	11.63	28.01	25.86	–	29.34	16.44	24.44	18.26	5.97	26.29	17.13	–	–	39.87	7.06	20.18	7.78	108
EKVX	37.48	24.46	25.93	53.99	19.72	29.56	27.92	24.10	26.64	66.74	66.66	–	–	55.62	9.22	25.37	7.18	94.3
HOP-62	–	51.18	–	–	74.09	–	–	–	–	6.81	13.44	nd	–	nd	nd	nd	nd	109.50
HOP-92	6.37	35.86	–	nd	51.76	34.60	23.13	31.66	30.70	nd	nd	–	–	15.22	99.07	52.70	32.33	96.20
NCI-H226	23.61	105.34	23.90	11.52	106.99	45.16	26.01	29.04	21.82	18.20	12.06	–	–	5.08	67.22	24.77	10.50	87.70
NCI-H23	6.36	85.13	17.94	7.05	40.56	25.24	13.40	11.65	22.82	17.71	17.03	14.06	12.32	84.74	47.90	42.70	20.81	97.60
NCI-H322M	–	19.44	–	–	17.70	10.24	6.24	5.89	6.18	–	–	–	10.32	9.70	6.14	–	–	84.90
NCI-H460	–	34.24	6.01	–	18.32	–	–	–	–	8.94	5.26	17.38	–	106.89	–	44.39	–	92.50
NCI-H522	–	37.21	–	11.15	51.70	33.32	–	7.04	–	28.39	25.73	7.23	–	5.70	42.45	21.77	8.27	129.40
Colon cancer																		
COLO 205	–	–	–	–	–	25.08	7.03	–	–	–	–	nd	–	nd	nd	nd	nd	180.1
HCC-2998	–	19.53	–	–	11.62	–	–	–	–	–	–	–	–	44.35	–	–	–	89.6
HCT-116	–	53.56	18.17	–	53.40	20.48	22.91	17.55	7.08	13.42	7.62	41.54	–	87.90	47.25	72.84	30.23	99.8
HCT-15	8.99	31.29	40.05	–	17.46	25.54	5.56	9.87	5.47	31.91	21.00	10.77	9.62	127.40	–	36.40	–	96.9
HT29	nd	nd	nd	–	–	nd	nd	nd	nd	14.83	9.08	–	–	20.32	–	6.74	–	116.2
KM12	6.23	18.69	15.67	–	15.34	8.64	8.90	8.46	5.79	22.47	12.55	–	–	36.53	–	17.57	–	99.4
SW-620	–	–	–	–	–	–	–	–	–	–	–	29.93	6.33	148.26	–	51.82	7.61	88.5
CNS cancer																		
SF-268	6.56	43.82	13.85	–	45.84	5.11	–	–	–	–	8.60	–	5.27	51.91	53.94	27.89	–	92.00
SF-295	14.46	44.34	20.67	–	32.61	29.36	19.06	13.86	11.71	38.31	33.14	–	–	31.59	89.39	23.77	–	84.70
SF-539	5.56	76.88	5.44	–	110.59	9.46	9.04	6.47	–	30.36	9.75	10.76	11.49	12.44	65.68	34.10	21.62	128.60
SNB-19	6.15	74.25	7.69	–	81.08	13.10	8.33	11.58	5.77	–	–	–	–	49.50	86.59	63.00	48.19	87.7
SNB-75	–	54.28	–	–	84.86	–	–	–	–	–	10.42	–	–	–	120.44	15.62	–	120.6
U251	–	56.51	–	–	86.92	8.36	5.89	–	–	–	–	13.36	–	143.46	78.07	63.50	30.21	91.50
Melanoma																		
LOX IMVI	–	84.01	9.06	8.86	59.05	25.54	–	–	–	13.16	19.74	18.28	–	160.95	13.12	72.29	38.27	94.70
MALME-3M	–	57.82	13.58	–	55.98	13.82	16.42	12.81	12.74	2.32	6.30	–	18.46	35.23	8.36	26.21	11.71	108.70
M14	–	11.64	5.86	–	8.00	–	9.67	7.92	8.57	–	–	–	–	39.13	–	23.59	9.47	87.5
MDA-MB-435	–	17.86	19.69	–	14.63	10.97	–	–	6.00	–	5.27	–	–	96.57	–	37.37	–	78.9
SK-MEL-2	nd	nd	nd	–	nd	nd	nd	nd	nd	12.78	9.60	–	–	26.06	115.65	56.54	17.63	nd
SK-MEL-28	–	–	–	–	–	–	–	–	–	–	–	–	6.91	37.85	–	–	–	83.80
SK-MEL-5	13.43	62.70	44.97	5.89	43.95	95.02	62.94	51.82	24.59	23.55	19.70	–	7.31	24.21	5.61	12.80	–	92.70
UACC-257	6.77	–	–	–	13.04	–	–	–	5.08	–	–	–	–	–	–	–	–	114.80
UACC-62	13.82	33.71	28.02	10.86	25.55	46.29	34.75	25.33	11.06	32.61	35.62	–	13.88	–	19.19	12.95	–	109.5
Ovarian cancer																		
IGROV1	–	35.91	–	–	42.62	7.95	6.64	–	–	–	11.35	–	–	18.68	41.60	16.12	–	97.50
OVCAR-3	–	79.13	–	–	81.63	–	–	–	–	–	–	–	–	94.12	–	31.89	–	79.30
OVCAR-4	13.08	99.49	32.22	–	90.58	27.14	20.46	20.14	5.61	13.16	11.40	16.50	-	68.35	13.06	25.24	–	87.10
OVCAR-5	–	7.29	–	–	–	–	–	–	–	8.99	–	–	7.10	9.28	–	–	–	90.90
OVCAR-8	–	57.37	19.15	–	71.28	10.62	12.04	11.59	–	12.69	10.38	20.84	–	93.38	38.18	59.73	38.56	91.10
NCI/ADR-RES	–	71.87	25.58	6.89	49.76	28.45	14.65	10.58	6.51	20.56	19.73	28.96	14.31	130.36	19.93	47.55	27.04	90.10
SK-OV-3	–	15.84	–	–	17.03	8.22	14.52	9.98	–	–	–	nd	–	nd	nd	nd	nd	116.90
Renal cancer																		
786-0	–	57.65	5.40	–	101.23	–	9.21	5.62	–	22.12	16.93	-	44.99	75.63	87.35	44.24	20.37	87.70
A498	–	5.48	–	–	12.61	–	–	–	–	–	–	–	–	–	20.06	–	–	161.60
ACHN	–	52.92	9.04	18.45	64.71	14.78	11.83	6.51	11.44	26.60	22.74	19.82	8.57	96.99	76.97	59.32	30.78	90.4
CAKI-1	22.46	41.90	28.09	6.21	51.85	34.75	36.68	24.98	28.19	39.78	40.19	9.28	12.63	72.22	45.22	50.03	29.61	92.30
RXF 393	5.86	134.59	23.06	–	82.91	35.85	25.38	15.41	9.05	37.81	14.21	–	nd	129.01	99.84	32.76	15.08	148.20
SN12C	7.64	61.93	12.43	8.28	67.11	18.49	9.88	14.66	11.67	14.00	16.14	7.15	5.76	24.01	37.68	36.67	20.61	88.70
TK-10	–	93.55	–	–	55.14	–	–	–	–	–	–	10.32	–	86.88	96.63	62.99	36.32	112.5
UO-31	26.54	27.53	35.66	–	21.89	41.81	38.34	30.29	25.41	33.24	30.14	–	–	44.24	19.08	20.65	15.64	108.7
Prostate cancer																		
PC-3	19.26	22.82	43.83	nd	27.83	44.41	46.62	43.62	18.80	nd	nd	7.63	–	49.56	11.94	26.21	16.13	92.10
DU-145	6.07	22.75	6.75	–	14.27	–	–	–	6.78	–	–	5.42	7.08	130.12	–	19.41	–	84.50
Breast cancer																		
MCF7	25.11	64.25	18.66	15.86	55.39	26.62	37.74	23.12	25.07	35.93	35.92	33.97	25.33	82.77	17.21	57.82	35.42	89.60
MDA-MB-231/ATCC	–	63.76	–	15.59	55.33	18.32	13.89	10.30	8.73	19.83	27.34	9.78	12.37	75.41	49.20	59.98	42.55	85.30
HS 578T	5.85	58.35	–	–	75.23	16.30	9.01	–	–	8.19	–	–	5.72	34.64	96.22	74.27	46.81	nd
BT-549	–	50.79	18.71	–	86.84	25.83	18.44	16.10	–	7.55	–	–	–	33.89	54.51	23.63	21.16	94.80
T-47D	12.02	48.54	36.38	–	60.76	29.32	41.06	37.57	–	40.62	41.03	nd	13.50	nd	nd	nd	nd	105.70
MDA-MB-468	–	90.93	29.51	5.76	63.10	50.47	26.10	30.66	6.21	27.13	19.79	8.24	–	60.54	17.86	–	–	113.40
Mean GI%	–	44.28	12.24	–	43.78	15.46	9.27	6.32	–	14.92	12.52	6.03	–	55.91	30.34	31.16	10.08	> 100

^a^GI% < 5%

^b^Not detected

The oxindole–benzothiazole hybrids **9a**–**r** displayed disparate growth inhibitory activity on NCI cell lines. The synthesized derivatives demonstrated mean growth inhibition percentage spanning from < 5% to 55.91% in reference to milciclib which showed a mean growth inhibitory activity more than 100% (Table 1).

**Fig. 5:**
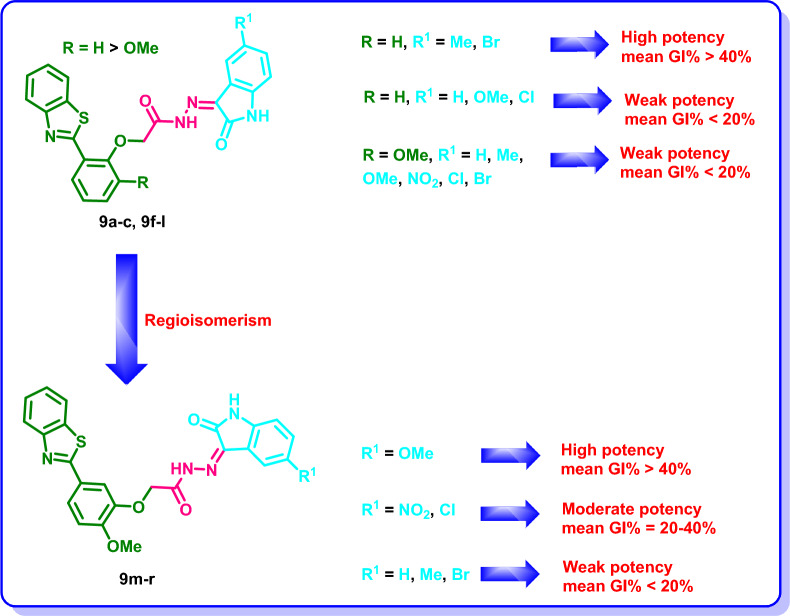
Structure–activity relationship of **9a**–**c** and **9e**–**r** on NCI cancer cell lines

In series **9a**–**f**, the 5-methyl and 5-bromo derivatives **9b** and **9f** showed the most promising inhibitory activity with mean growth inhibition % = 44.28 and 43.78%, respectively, while the unsubstituted oxindole derivative **9a** (mean GI% < 5%) and the chloro substituted oxindole derivative **9e** (mean GI% < 5%) demonstrated the weakest activity on the NCI cancer cell lines (Table 1, Fig. 5).

The introduction of a methoxy group in series **9g**–**l** resulted in a decrease in the mean growth inhibition % for **9h** (mean GI% = 9.27%), **9i** (mean GI% = 6.32%) and **9l** (mean GI% = 12.52%) in reference to **9b** (mean GI% = 44.28%), **9c** (mean GI% = 12.24%) and **9f** (mean GI% = 43.78%), respectively. Meanwhile, an increase in the potency was observed for **9g** (mean GI% = 15.46%) and **9k** (mean GI% = 14.92%) in reference to **9a** (mean GI% < 5%) and **9e** (mean GI% < 5%) (Table 1, Fig. 5).

The regioisomers **9m**–**r** demonstrated a decrease in the potency for **9m** (mean GI% = 6.03%), **9n** (mean GI% < 5%) and **9r** (mean GI% = 10.08%) in reference to **9g** (mean GI% = 15.46%), **9h** (mean GI% = 9.27%) and **9l** (mean GI% = 12.52%), respectively, while an increase in the potency was observed for the derivatives **9o**, **9p** and **9q** (mean GI% = 30.34 to 55.91%) exhibiting 5-methoxy, 5-nitro and 5-chloro substituents, respectively (Table 1, Fig. 5).

#### Antiproliferative activity of 9o on NCI cancer cell lines at five concentrations

**Fig. 6:**
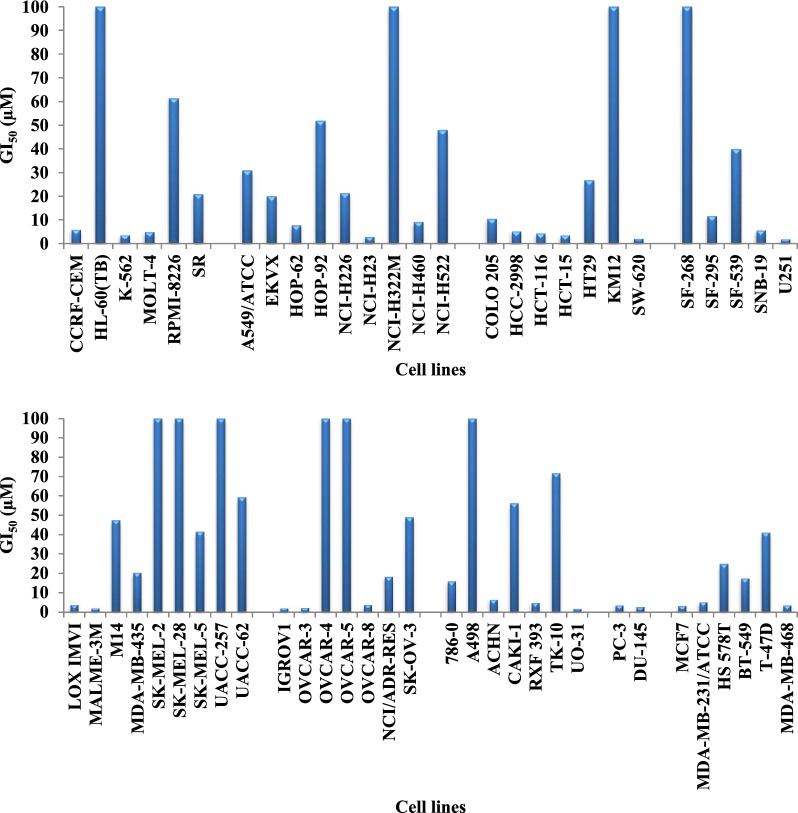
GI_50_ (µM) of **9o** against diverse cancer cell lines

Encouraged by the potent activity of **9o** on diverse cancer cell lines on the one-dose assay (Table 1), it was further selected to be examined at 5-dose concentrations and the GI_50_ was depicted in Table 2 and Fig. 6 (for additional details see Additional file 1: dose-response curves of **9o** on NCI cancer cell lines). The oxindole–benzothiazole hybrid **9o** revealed moderate to potent potency against the tested cell lines (GI_50_ reaching 2.02 µM). Close examination showed that **9o** displayed GI_50_ of 3.75 µM on the K-562 cell line from leukemia, GI_50_ = 3.03 µM on the NCI-H23 cell line from non-small cell lung cancer. HCT-116, HCT-15 and SW-620 cell lines from colon cancer are sensitive to **9o** with GI_50_ = 4.50, 3.60 and 2.27 µM, respectively. Also, the U251 cell line from CNS cancer is very sensitive to **9o** (GI_50_ = 2.02 µM). Additionally, **9o** demonstrated GI_50_ = 4.09 and 2.28 µM on LOX IMVI and MALME-3M cell lines, respectively from melanoma; GI_50_ = 2.22, 2.49 and 4.02 µM on IGROV1, OVCAR-3 and OVCAR-8 cell lines, respectively from ovarian cancer; GI_50_ = 2.03 µM on UO-31 cell line from renal cancer; GI_50_ = 3.78 and 2.92 µM on PC-3 and DU-145 cell lines, respectively from prostate cancer and GI_50_ = 3.44 and 3.72 µM on MCF7 and MDA-MB-468 cell lines, respectively from breast cancer.

**Table 2:** GI_50_ (µM) of oxindole**–**benzothiazole hybrid **9o** on NCI cancer cell lines

Cell name	**9o** GI_50_ (µM)	Cell name	**9o** GI_50_ (µM)
Leukemia		M14	47.4
CCRF-CEM	6.08	MDA-MB-435	20.6
HL-60 (TB)	> 100	SK-MEL-2	> 100
K-562	3.75	SK-MEL-28	> 100
MOLT-4	5.05	SK-MEL-5	41.5
RPMI-8226	61.4	UACC-257	> 100
SR	20.9	UACC-62	59.3
Non-small cell lung cancer		Ovarian cancer	
A549/ATCC	30.9	IGROV1	2.22
EKVX	20.0	OVCAR-3	2.49
HOP-62	7.84	OVCAR-4	> 100
HOP-92	51.9	OVCAR-5	> 100
NCI-H226	21.3	OVCAR-8	4.02
NCI-H23	3.03	NCI/ADR-RES	18.5
NCI-H322M	> 100	SK-OV-3	48.9
NCI-H460	9.26	Renal cancer	
NCI-H522	48.0	786-0	16.1
Colon cancer		A498	> 100
COLO 205	10.6	ACHN	6.70
HCC-2998	5.34	CAKI-1	56.2
HCT-116	4.50	RXF 393	5.06
HCT-15	3.60	SN12C	25.6
HT29	26.8	TK-10	71.7
KM12	> 100	UO-31	2.03
SW-620	2.27	Prostate cancer	
CNS cancer		PC-3	3.78
SF-268	> 100	DU-145	2.92
SF-295	11.8	Breast cancer	
SF-539	39.9	MCF7	3.44
SNB-19	5.72	MDA-MB-231/ATCC	5.44
SNB-75	nd^a^	HS 578T	24.8
U251	2.02	BT-549	17.6
Melanoma		T-47D	41.0
LOX IMVI	4.09	MDA-MB-468	3.72
MALME-3M	2.28

#### Effect of 9o on the cell cycle of DU145 prostate cancer

**Fig. 7:**
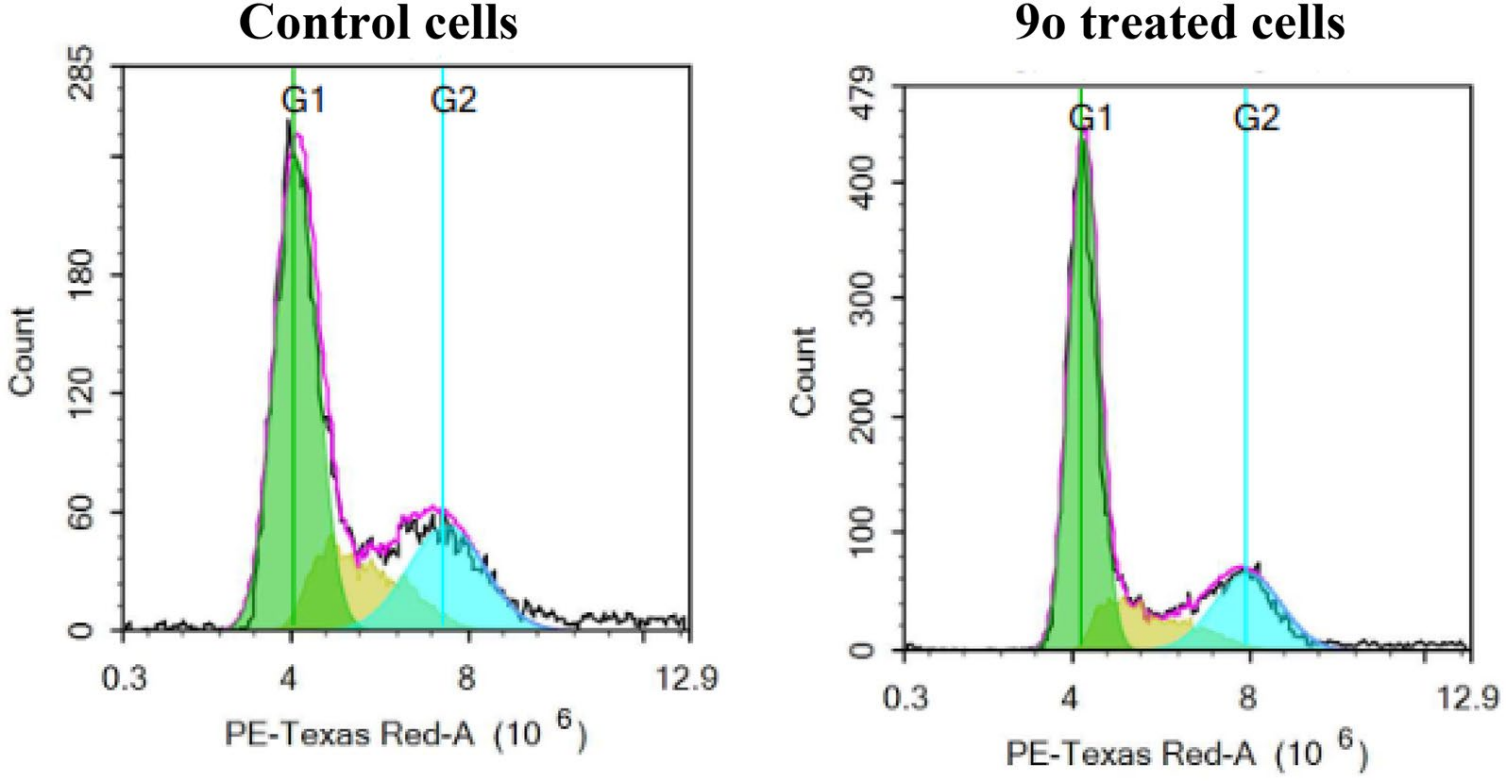
Cell cycle of DU145 before and after treatment with **9o**

Motivated by the potent activity of **9o** on prostate cancer cell lines in Table 2, it was further examined for its effect on the cell cycle of the DU145 cell line at its GI_50_ concentration and the results were depicted in Fig. 7 and Table 3. Obviously, **9o** proved the ability to arrest the cell cycle of the DU-145 cell line at the G1 phase as the % of cells accumulated in the G1 phase raised from 57.91% in control cells to 61.40% in **9o** treated cells. Concurrently, there is a decline in the % of cells in the G2 phase from 22.20% in control cells to 20.94% in **9o** treated cells.

**Table 3:** Different phases of cell cycle of DU145 before and after treatment with **9o**

Comp.	%G0/G1	%S	%G2/M	%Sub-G1
Control	57.91	19.89	22.20	0.88
**9o**	61.40	17.66	20.94	0.41

#### Apoptotic effect of 9o on DU145 prostate cancer

**Fig. 8:**
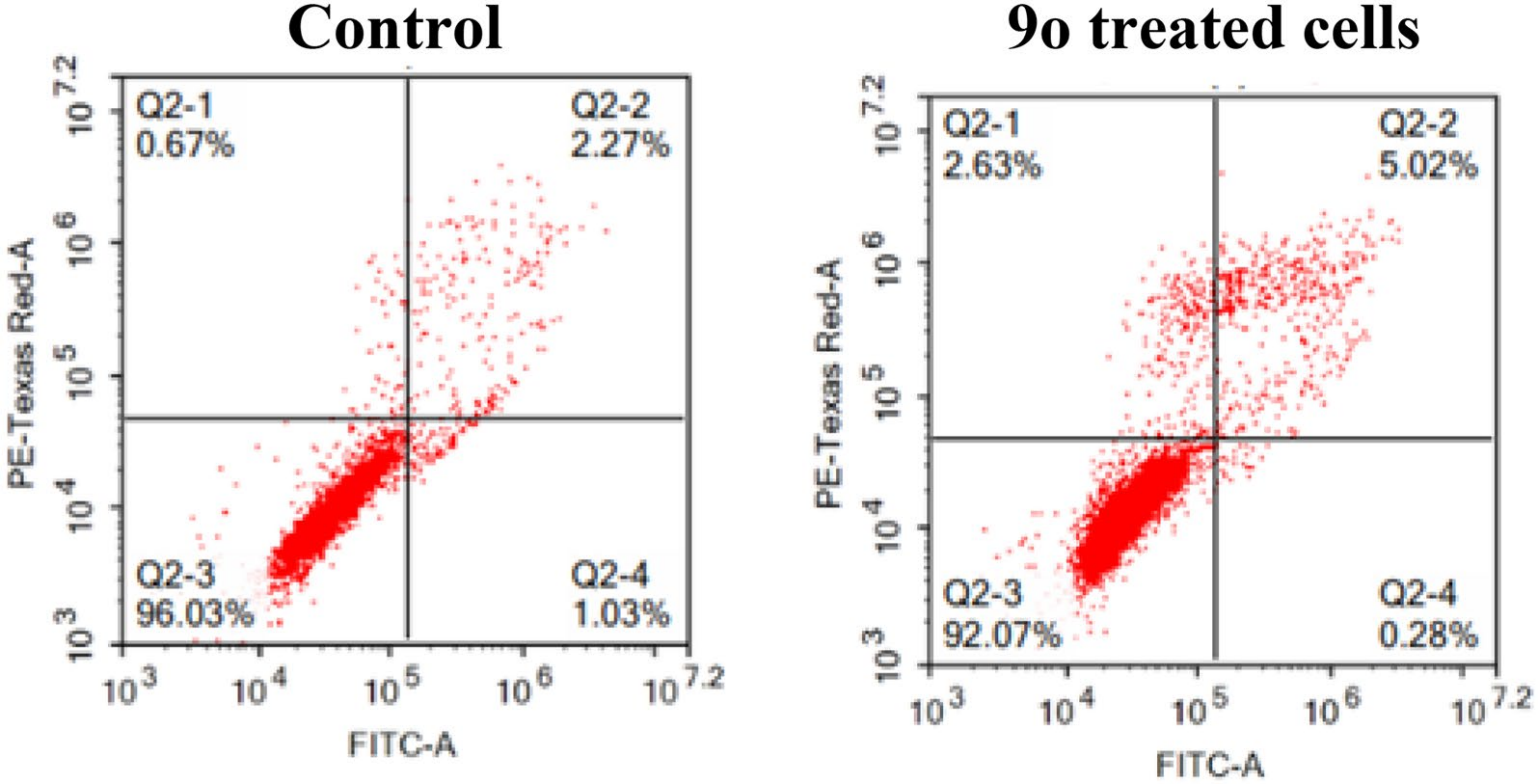
DU145 cell line before and after treatment with **9o** (Q2–3, viable; Q2–4, early apoptotic; Q2–2, late apoptotic; Q2–1, necrotic)

In parallel, the capability of **9o** to potentiate the apoptosis of the DU145 cell line was explored at its GI_50_ concentration. The presented results in Fig. 8 confirm the potency of **9o** to induce the apoptosis and necrosis of the DU145 cell line as the % of cells in the late apoptotic stage elevated from 2.27% in control cells to 5.02% in treated cells. Also, Fig. 8, showed that **9o** increased the number of cells in the necrotic stage from 0.67% in control cells to 2.63% in treated cells.

#### Inhibitory activity of selected candidates on CDK2

The oxindole–benzothiazole conjugates **9b, 9f** and **9o** were assayed for their potential to suppress the activity of CDK2 and the results were represented as the IC_50_ in µM and compared with staurosporine as a standard (Table 4).

**Table 4:** Inhibitory activity of the oxindole–benzothiazole conjugates **9b**, **9f** and **9o** on CDK2


Compound ID	R^1^	CDK2 (IC_50_ in µM)^a^
**9b**	Me	0.70 ± 0.013
**9f**	Br	0.20 ± 0.035
**9o**	–	0.21 ± 0.015
**Staurosporine**	–	0.022 ± 0.002

^a^Results are mean of two independent experiments ± standard deviation (SD)

From the obtained results it is obvious that compounds **9b**, **9f** and **9o** are potential inhibitors of CDK2 with IC_50_ = 0.70, 0.20 and 0.21 µM. Compounds **9f** and **9o** revealed the most potent inhibitors followed by **9b** (Table 4).

#### Inhibitory activity of 9o on diverse kinases

Subsequently, the conjugate **9o** was examined for its inhibitory activity on CDK1 and CDK5 isoforms as well as for its inhibitory activity on VEGFR-2 and FGFR-1 and the outcomes were presented in Table 5.

**Table 5:** Inhibitory activities of the oxindole–benzothiazole conjugate **9o** on different kinases

Compound ID	(IC_50_ in µM)^a^			
	CDK1	CDK5	VEGFR-2	FGFR-1
**9o**	1.19 ± 0.10	0.34 ± 0.02	> 10	> 10
**Staurosporine**	0.002 ± 0.0001	0.001 ± 0.0001	nd^b^	nd^b^
**Sorafenib**	nd^b^	nd^b^	0.10 ± 0.01	0.58 ± 0.10

^a^Results are mean of two independent experiments ± standard deviation (SD)

^b^Not detected

It was found that **9o** exhibited IC_50_ = 1.19 and 0.34 µM, respectively on CDK1 and CDK5 respectively. Meanwhile, IC_50_ > 10 µM was detected against VEGFR-2 and FGFR-1 (Table 5). The results presented in Tables 4 and 5 showed that **9o** exhibit higher selectivity toward CDK2 and CDK5 over CDK1, VEGFR-2 and FGFR-1.

### Molecular docking simulation

**Fig. 9.**
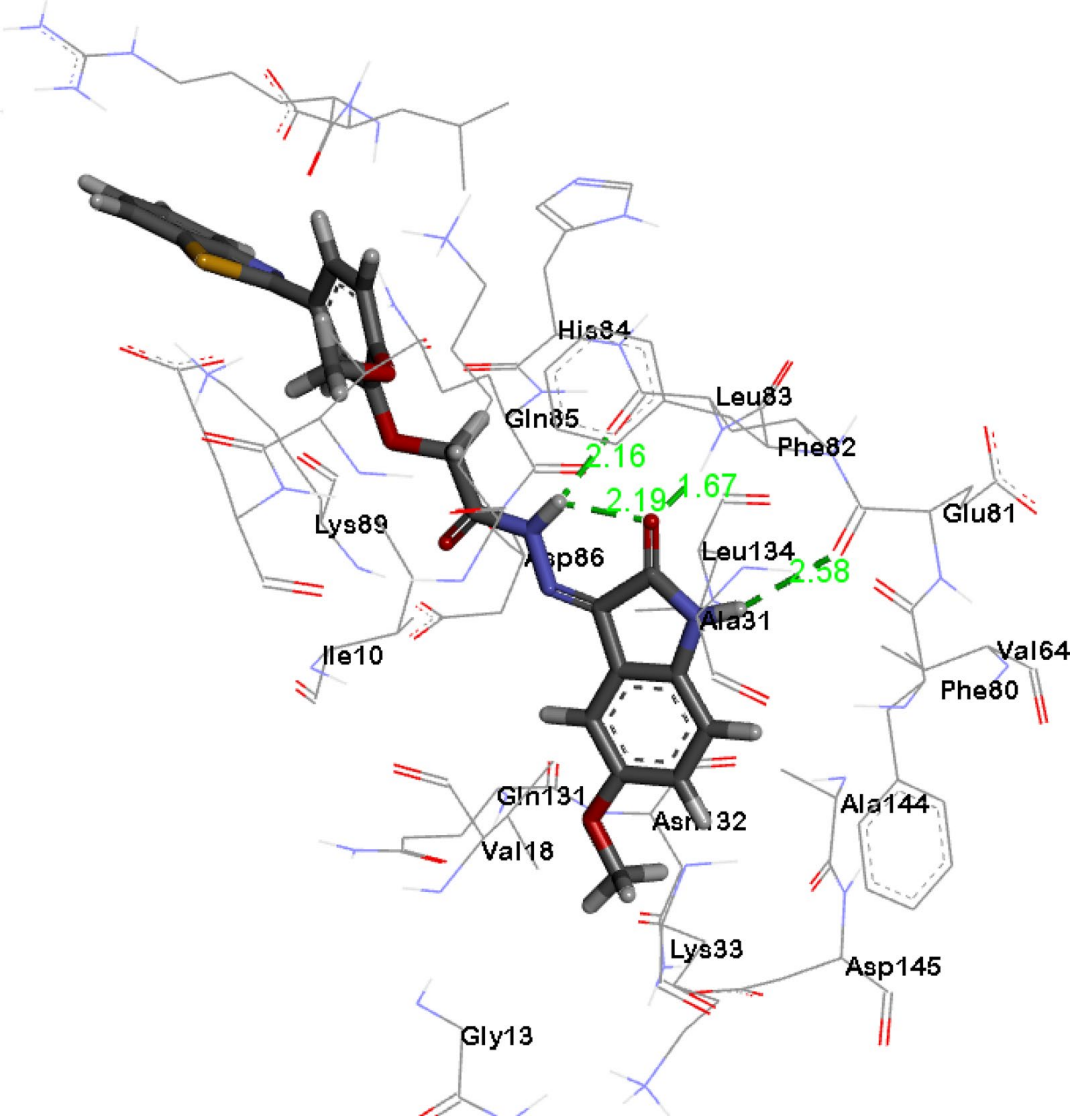
3D Diagram of **9o** showing its interaction with CDK2 active site

To confirm the expected mode of binding of the oxindole–benzothiazole hybrids **9a**–**r** to CDK2, compound **9o** was selected to be docked into the binding pocket of CDK2 using Autodock Vina [33] and the results were visualized using BIOVIA Discovery Studio Visualizer https://discover.3ds.com/discovery-studio-visualizer. First, the crystal structure of CDK2 (PDB ID: 1FVT) [34] was retrieved from the protein data bank and the protein was prepared followed by re-docking of the native ligand to validate the protocol that will be employed for the docking study (for further details see Additional file 1: docking of the co-crystalized ligand in the binding site of CDK2). Afterward, the oxindole–benzothiazole hybrid **9o** was docked into CDK2’s binding pocket and the results were analysed [16]. The synthesized oxindole–benzothiazole hybrid **9o** expressed higher affinity to the active site of CDK2 with docking energy scores (*S*) − 10.8 kcal/mol in relevance to the native ligand docking energy score (*S*) of − 9.1 kcal/mol. As shown in Fig. 9, the oxindole part of the oxindole–benzothiazole scaffold **9o** is settled in the ATP binding pocket where the lactam ring performs hydrogen bonding with the key amino acids Glu81 and Leu83, and the NH group of the acetohydrazide is involved in hydrogen bonding with Leu83, while the fused phenyl ring participates in hydrophobic interactions with the adjacent amino acid residues Val18, Ala31, Leu134, Ala144 and Asp145. Meanwhile, the 2-phenyl benzothiazole moiety is directed toward the solvent region where it creates hydrophobic interactions with the amino acids Ile10, Lys20, Lys89, Arg297 and Leu298 at the binding pocket’s entrance (Fig. 9).

### ADME properties prediction

**Fig. 10:**
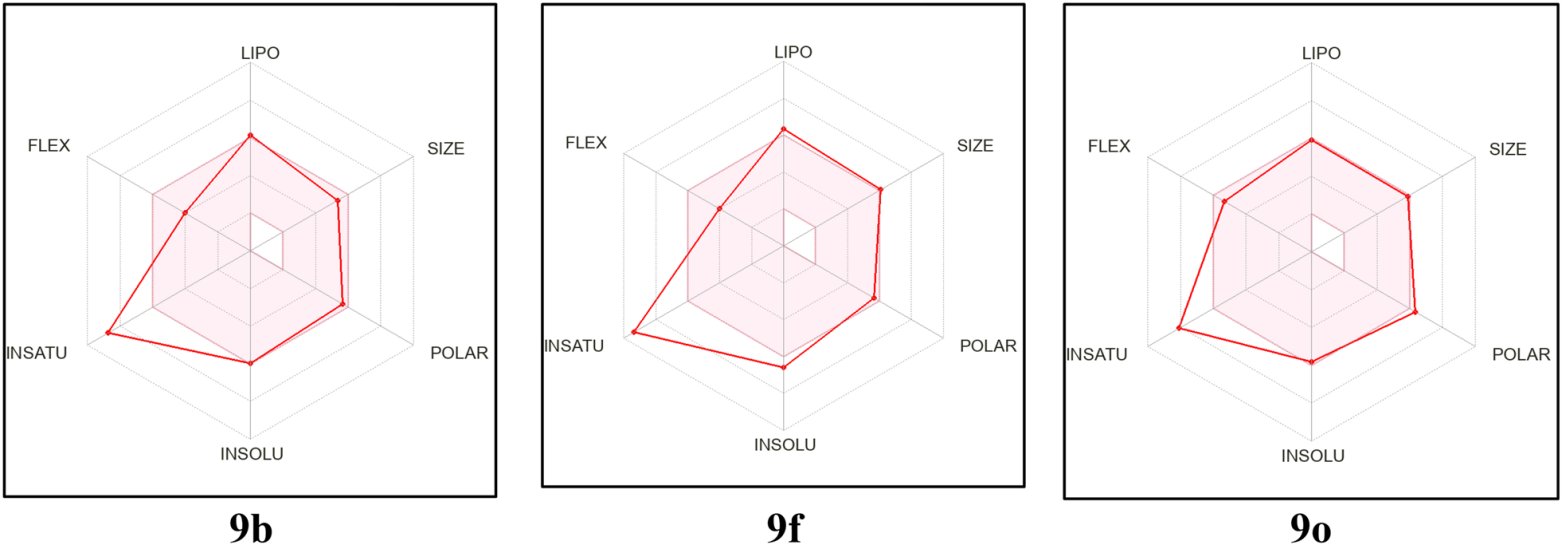
Bioavailability radar Chart for compounds **9b**, **9f** and **9o** from SwissADME webtool [35]

The synthesized oxindole–benzothiazole hybrids **9a**–**r** were tested using the SwissADME online tool to determine their drug similarity and ADME characteristics [35]. Table 6 demonstrates some selected findings. The majority of the hybrids **9a**–**r** satisfy Lipinski’s criterion of 5 [36–38], the derivatives **9f**, **9j**, **9l**, **9p** and **9r** are the only instances that exhibit one or two violations. It is anticipated that none of the submitted oxindole–benzothiazole hybrids **9a**–**r** are sufficiently lipophilic to cross the blood–brain barrier, highlighting the absence of any anticipated central effects [39]. All the synthesized candidates are not substrates to P-glycoprotein (P-gp) which is the primary transporter of xenobiotics to the outside of the cells [40]. The majority of the provided hits have a bioavailability score of 0.55, indicating that they are mostly orally bioavailable. Furthermore, the bioavailability radar charts of oxindole–benzothiazoles **9b**, **9f** and **9o** are shown in Fig. 10 (for further details see additional file 1: bioavailability radar charts for **9a**–**r** from SwissADME free webtool). They highlight ideal size, polarity, flexibility, and solubility for oral bioavailability. The only characteristic that deviates slightly from its ideal value is the degree of saturation. As a conclusion, we can summarize that in addition to the potential CDK2 inhibitory action as targeted anticancer agents, the oxindole–benzothiazole hybrids **9a**–**r** displayed acceptable ADME qualities that can be further optimized as anticancer agents.

**Table 6:** Physicochemical properties of oxindole–benzothiazole hybrids **9a**–**r** from SwissADME [35]

Compound ID	MW	#Rotatable bonds	#H-bond acceptors	#H-bond donors	MR	TPSA	iLOGP	BBB permeant	Pgp substrate	Lipinski #violations	Bioavailability score	Synthetic accessibility
**9a**	428.46	6	5	2	122.66	120.92	3.08	No	No	0	0.55	3.42
**9b**	442.49	6	5	2	127.63	120.92	3.29	No	No	0	0.55	3.55
**9c**	458.49	7	6	2	129.15	130.15	2.81	No	No	0	0.55	3.6
**9d**	473.46	7	7	2	131.48	166.74	2.42	No	No	0	0.55	3.57
**9e**	462.91	6	5	2	127.67	120.92	2.93	No	No	0	0.55	3.4
**9f**	507.36	6	5	2	130.36	120.92	2.88	No	No	1	0.55	3.49
**9g**	458.49	7	6	2	129.15	130.15	2.69	No	No	0	0.55	3.57
**9h**	472.52	7	6	2	134.12	130.15	3.02	No	No	0	0.55	3.7
**9i**	488.52	8	7	2	135.65	139.38	3.23	No	No	0	0.55	3.77
**9j**	503.49	8	8	2	137.98	175.97	2.36	No	No	2	0.17	3.72
**9k**	492.93	7	6	2	134.16	130.15	3.09	No	No	0	0.55	3.56
**9l**	537.39	7	6	2	136.85	130.15	3.34	No	No	1	0.55	3.64
**9m**	458.49	7	6	2	129.15	130.15	3.39	No	No	0	0.55	3.52
**9n**	472.52	7	6	2	134.12	130.15	3.52	No	No	0	0.55	3.65
**9o**	488.52	8	7	2	135.65	139.38	3.26	No	No	0	0.55	3.71
**9p**	503.49	8	8	2	137.98	175.97	2.5	No	No	2	0.17	3.67
**9q**	492.93	7	6	2	134.16	130.15	3.25	No	No	0	0.55	3.51
**9r**	537.39	7	6	2	136.85	130.15	3.42	No	No	1	0.55	3.59

## Conclusion

The construction of a new scaffold of oxindole–benzothiazole conjugates **9a**–**r** as CDK2 inhibitors and anticancer drugs was accomplished through the use of the molecular hybridization technique. The scaffold was synthesized using conventional organic synthesis techniques. Various spectral data were utilized to verify the structures of the afforded candidates. Examining the produced candidates’ growth inhibitory activity on NCI cancer cell lines demonstrated their weak to strong growth inhibitory effect. Specifically, **9o** displayed a strong GI_50_ that reached 2.02 µM. DU145 cell line from prostate cancer was examined for how **9o** affected its cell cycle, and it was found that **9o** stopped the cell cycle at the G1 phase. Additionally, **9o** demonstrated its capacity to induce late apoptosis and necrosis, which accelerate the cell death of the DU145 cell line. Additionally, the oxindole–benzothiazole conjugates **9b**, **9f** and **9o** showed potent CDK2 inhibitory activity with IC_50_ = 0.70, 0.20 and 0.21 µM, respectively. Moreover, **9o** was found to have higher selectivity toward CDK2 and CDK5 over CDK1, VEGFR-2 and FGFR-1. In silico docking of **9o** into CDK2 active site proved the predicted binding mode in which the oxindole moiety is settled in the ATP binding pocket and is involved in hydrogen bonding interactions with the key amino acids Glu81 and Leu83 as well as hydrophobic interaction with the amino acid residues lining the hinge region, while the benzothiazole moiety is directed towards the solvent region. Additionally, the proposed oxindole–benzothiazole hybrids **9a**–**r** exhibit acceptable physicochemical and pharmacokinetics qualities that can be further optimized as anticancer agents.

## Experimental

### Chemistry

#### General remarks

Chemicals that were used in organic synthesis and for biological screening were picked up from commercial companies. The chemical reactions were followed up employing pre-coated silica gel 60 F_245_ aluminium plates (Merck). Melting points of the synthesized molecules were recorded on a Stuart SMP30 melting point instrument. Spectroscopic measurements and elemental analysis of the synthesized organic derivatives were afforded in the Micro analytical labs, National Research Centre, Cairo, Egypt. A Jasco FT/IR 300 E Fourier transform infrared spectrophotometer was used for measuring the IR spectra (4000–400 cm^−1^). Bruker instruments 500 (125) MHz and 400 (100) MHz were used for recording the ^1^H NMR and ^13^C NMR (DMSO-*d*_6_).

#### General procedure for the synthesis of 9a–r

Equimolar amounts of 2-phenylbenzothiazole aceto hydrazides **7a**–**c** (0.50 mmol) and **8a**–**f** (0.5 mmol) were reacted together in ethanol (20 mL) containing glacial acetic acid (1 mL) at 80 °C for 4h. Then filtration of the precipitated products **9a**–**r** followed by drying and crystallization from ethanol was performed to afford analytically pure derivatives **9a**–**r** in good yields (Additional file 1: NMR spectra of oxindole–benzothiazole hybrids **9a**–**r**; IR charts of the synthesized oxindole–benzothiazoles).

##### 2-(2-(Benzo[*d*]thiazol-2-yl)phenoxy)-*N*′-(2-oxoindolin-3-ylidene)acetohydrazide (**9a**)

Pale brown powder; yield = 73%; mp 238–240 °C; IR (KBr) *ṽ* 3221, 3148, 3059, 3036, 2959, 1721, 1694, 1493, 1462 cm^−1^; ^1^H NMR (400 MHz; DMSO-*d*_6_) *δ*_H_ 5.25 (s, 1H), 5.64 (s, 1H), 6.92 (s, 1H), 7.09 (t, ^3^*J* = 7.6 Hz, 1H), 7.22 (t like, ^3^*J* = 6.4 Hz, 1H), 7.32 (d, ^3^*J* = 8.4 Hz, 1H), 7.38 (t, ^3^*J* = 7.6 Hz, 1H), 7.43 (t, ^3^*J* = 7.2 Hz, 1H), 7.54 (t, ^3^*J* = 7.2 Hz, 2H), 7.58 (d, ^3^*J* = 7.6 Hz, 1H), 8.07 (d, ^3^*J* = 8.0 Hz, 1H), 8.12 (d, ^3^*J* = 7.6 Hz, 1H), 8.47 (dd, ^3^*J* = 7.6 Hz, ^4^*J* = 1.2 Hz, 1H), 11.23 (br., 1H), 13.52 ppm (br., 1H); ^13^C NMR (100 MHz; DMSO-*d*_6_) *δ*_C_ 68.50, 111.26, 113.71, 113.99, 119.63, 121.09, 121.86, 122.55, 122.72, 125.05, 126.32, 129.03, 132.01, 132.33, 135.64, 142.68, 151.62, 155.61, 162.48 ppm; Anal. Calcd for C_23_H_16_N_4_O_3_S: C, 64.47; H, 3.76; N, 13.08. Found: C, 64.15; H, 4.00; N, 13.31.

##### 2-(2-(Benzo[*d*]thiazol-2-yl)phenoxy)-*N*′-(5-methyl-2-oxoindolin-3-ylidene)acetohydrazide (**9b**)

Pale brown powder; yield = 65%; mp 251–253 °C; IR (KBr) *ṽ* 3206, 3075, 2936, 1720, 1697, 1628, 1601, 1489, 1454 cm^−1^; ^1^H NMR (400 MHz; DMSO-*d*_6_) *δ*_H_ 2.29 (s, 3H), 5.24 (s, 1H), 5.64 (s, 1H), 6.81 (s, 1H), 7.18–7.22 (m, 2H), 7.32 (d, ^3^*J* = 7.6 Hz, 1H), 7.40 (s, 1H), 7.44 (t, ^3^*J* = 7.2 Hz, 1H), 7.54 (t, ^3^*J* = 6.8 Hz, 2H), 8.07 (d, ^3^*J* = 7.6 Hz, 1H), 8.12 (d, ^3^*J* = 7.6 Hz, 1H), 8.47 (d, ^3^*J* = 7.2 Hz, 1H), 11.17 (s, 1H), 13.50 ppm (br., 1H); ^13^C NMR (100 MHz; DMSO-*d*_6_) *δ*_C_ 20.51, 65.48, 110.98, 113.79, 119.12, 119.60, 121.36, 121.81, 122.50, 123.15, 125.00, 126.26, 131.77, 132.29, 132.37, 135.60, 139.49, 140.39, 151.58, 155.35, 162.53 ppm; Anal. Calcd for C_24_H_18_N_4_O_3_S: C, 65.15; H, 4.10; N, 12.66. Found: C, 65.37; H, 4.32; N, 12.31.

##### 2-(2-(Benzo[*d*]thiazol-2-yl)phenoxy)-*N*′-(5-methoxy-2-oxoindolin-3-ylidene)acetohydrazide (**9c**)

Yellowish red powder; yield = 69%; mp 240–242 °C; IR (KBr) *ṽ* 3183, 3067, 3005, 2970, 1728, 1686, 1593, 1485 cm^−1^; ^1^H NMR (400 MHz; DMSO-*d*_6_) *δ*_H_ 3.76 (s, 3H), 5.25 (s, 1H), 5.66 (s, 1H), 6.84 (s, 1H), 6.95 (d, ^3^*J* = 7.2 Hz, 1H), 7.15 (br., 1H), 7.22 (br., 1H), 7.32 (d, ^3^*J* = 8.4 Hz, 1H), 7.43 (t, ^3^*J* = 7.2 Hz, 1H), 7.54 (t, ^3^*J* = 7.6 Hz, 2H), 8.07 (d, ^3^*J* = 8.0 Hz, 1H), 8.12 (d, ^3^*J* = 8.0 Hz, 1H), 8.47 (d, ^3^*J* = 7.2 Hz, 1H), 11.07 (s, 1H), 13.53 ppm (br., 1H); ^13^C NMR (100 MHz; DMSO-*d*_6_) *δ*_C_ 55.66, 67.77, 106.04, 112.03, 113.86, 118.16, 120.33, 121.80, 122.50, 124.98, 126.25, 128.98, 132.27, 135.59, 136.29, 151.57, 155.40, 162.56 ppm; Anal. Calcd for C_24_H_18_N_4_O_4_S: C, 62.87; H, 3.96; N, 12.22. Found: C, 62.50; H, 3.71; N, 12.51.

##### 2-(2-(Benzo[*d*]thiazol-2-yl)phenoxy)-*N*′-(5-nitro-2-oxoindolin-3-ylidene)acetohydrazide (**9d**)

Yellowish brown powder; yield = 62%; mp 275–277 °C; IR (KBr) *ṽ* 3229, 3159, 3086, 2920, 1721, 1624, 1605, 1520, 1497 cm^−1^; ^1^H NMR (400 MHz; DMSO-*d*_6_) *δ*_H_ 5.64 (s, 2H), 7.12 (d, ^3^*J* = 8.4 Hz, 1H), 7.22 (t, ^3^*J* = 7.2 Hz, 1H), 7.34 (d, ^3^*J* = 8.4 Hz, 1H), 7.43 (t, ^3^*J* = 7.6 Hz, 1H), 7.52–7.57 (m, 2H), 8.07 (d, ^3^*J* = 8.4 Hz, 1H), 8.12 (d, ^3^*J* = 8.0 Hz, 1H), 8.29 (dd, ^3^*J* = 8.8 Hz, ^4^*J* = 2.0 Hz, 1H), 8.35 (br., 1H), 8.46 (dd, ^3^*J* = 8.0 Hz, ^4^*J* = 1.2 Hz, 1H), 11.85 (s, 1H), 12.51 ppm (br., 1H); ^13^C NMR (100 MHz; DMSO-*d*_6_) *δ*_C_ 68.10, 111.47, 113.83, 116.16, 120.45, 121.81, 122.50, 125.00, 126.27, 127.67, 128.99, 132.28, 135.60, 142.85, 147.76, 151.57, 155.60, 162.21, 162.70 ppm; Anal. Calcd for C_23_H_15_N_5_O_5_S: C, 58.35; H, 3.19; N, 14.79. Found: C, 58.61; H, 3.44; N, 14.50.

##### 2-(2-(Benzo[*d*]thiazol-2-yl)phenoxy)-*N*′-(5-chloro-2-oxoindolin-3-ylidene)acetohydrazide (**9e**)

Yellow powder; yield = 72%; mp 265–267 °C; IR (KBr) *ṽ* 3217, 3171, 3136, 3082, 3024, 2990, 1709, 1624, 1600, 1581, 1497 cm^−1^; ^1^H NMR (400 MHz; DMSO-*d*_6_) *δ*_H_ 5.34 (s, 1H), 5.64 (s, 1H), 6.94 (d, ^3^*J* = 8.0 Hz, 1H), 7.23 (t, ^3^*J* = 7.6 Hz, 1H), 7.33 (d, ^3^*J* = 8.4 Hz, 1H), 7.41–7.46 (m, 2H), 7.52–7.56 (m, 2H), 7.62 (br., 1H), 8.07 (d, ^3^*J* = 8.0 Hz, 1H), 8.12 (d, ^3^*J* = 7.6 Hz, 1H), 8.47 (dd, ^3^*J* = 7.6 Hz, ^4^*J* = 1.6 Hz, 1H), 11.34 (s, 1H), 12.57 ppm (br., 1H); ^13^C NMR (100 MHz; DMSO-*d*_6_) *δ*_C_ 68.74, 112.79, 113.85, 120.71, 121.41, 121.85, 122.54, 125.06, 126.32, 126.86, 129.05, 131.31, 132.35, 135.63, 141.35, 151.61, 155.58, 162.25, 166.51 ppm; Anal. Calcd for C_23_H_15_ClN_4_O_3_S: C, 59.68; H, 3.27; N, 12.10. Found: C, 59.90; H, 3.06; N, 12.36.

##### 2-(2-(Benzo[*d*]thiazol-2-yl)phenoxy)-*N*′-(5-bromo-2-oxoindolin-3-ylidene)acetohydrazide (**9f**)

Yellow powder; yield = 75%; mp 261–263 °C; IR (KBr) *ṽ* 3364, 3302, 3221, 3183, 3132, 3067, 2920, 2851, 1719, 1709, 1578, 1497 cm^−1^; ^1^H NMR (400 MHz; DMSO-*d*_6_) *δ*_H_ 5.32 (s, 1H), 5.64 (s, 1H), 6.89 (d, ^3^*J* = 7.6 Hz, 1H), 7.22 (t like, ^3^*J* = 7.2 Hz, 1H), 7.33 (d, ^3^*J* = 8.0 Hz, 1H), 7.44 (t, ^3^*J* = 7.6 Hz, 1H), 7.53–7.55 (m, 3H), 7.74 (br., 1H), 8.07 (d, ^3^*J* = 8.0 Hz, 1H), 8.12 (d, ^3^*J* = 8.0 Hz, 1H), 8.47 (d, ^3^*J* = 7.6 Hz, 1H), 11.35 (s, 1H), 12.54 ppm (br., 1H); ^13^C NMR (100 MHz; DMSO-*d*_6_) *δ*_C_ 67.71, 113.18, 113.77, 114.39, 121.82, 122.51, 123.40, 125.00, 126.27, 128.98, 132.28, 134.05, 135.61, 141.68, 151.58, 155.51, 162.06 ppm; Anal. Calcd for C_23_H_15_BrN_4_O_3_S: C, 54.45; H, 2.98; N, 11.04. Found: C, 54.23; H, 2.65; N, 11.37.

##### 2-(2-(Benzo[*d*]thiazol-2-yl)-6-methoxyphenoxy)-*N*′-(2-oxoindolin-3-ylidene)acetohydrazide (**9g**)

Pale brown powder; yield = 75%; mp 246–248 °C; IR (KBr) *ṽ* 3179, 3148, 3067, 3021, 2974, 2901, 1732, 1690, 1620, 1582, 1516, 1466 cm^−1^; ^1^H NMR (400 MHz; DMSO-*d*_6_) *δ*_H_ 3.87 (s, 3H), 4.87 (s, 2H), 6.93 (d, ^3^*J* = 7.6 Hz, 1H), 7.12 (t like, ^3^*J* = 7.0 Hz, 1H), 7.33 (br., 2H), 7.38 (d like, ^3^*J* = 8.0 Hz, 1H), 7.43 (t, ^3^*J* = 8.0 Hz, 1H), 7.52 (t, ^3^*J* = 8.0 Hz, 1H), 7.62 (d like, ^3^*J* = 5.6 Hz, 1H), 7.98 (d, ^3^*J* = 6.0 Hz, 1H), 8.05 (d, ^3^*J* = 8.0 Hz, 1H), 8.12 (d, ^3^*J* = 8.0 Hz, 1H), 11.19 (s, 1H), 13.84 ppm (br., 1H); ^13^C NMR (100 MHz; DMSO-*d*_6_) *δ*_C_ 56.31, 71.13, 111.16, 115.38, 119.82, 120.07, 121.11, 122.06, 122.72, 125.37, 126.21, 126.42, 131.98, 135.38, 138.54, 142.73, 144.80, 151.77, 152.33, 161.81, 162.38, 165.61 ppm; Anal. Calcd for C_24_H_18_N_4_O_4_S: C, 62.87; H, 3.96; N, 12.22. Found: C, 62.75; H, 3.71; N, 12.47.

##### 2-(2-(Benzo[*d*]thiazol-2-yl)-6-methoxyphenoxy)-*N*′-(5-methyl-2-oxoindolin-3-ylidene)acetohydrazide (**9h**)

Yellow powder; yield = 80%; mp 244–246 °C; IR (KBr) *ṽ* 3233, 3055, 3020, 2974, 2916, 1740, 1701, 1628, 1582, 1474 cm^−1^; ^1^H NMR (400 MHz; DMSO-*d*_6_) *δ*_H_ 2.31 (s, 3H), 3.87 (s, 3H), 4.86 (s, 2H), 6.81 (d, ^3^*J* = 7.6 Hz, 1H), 7.19 (d, ^3^*J* = 7.2 Hz, 1H), 7.32 (br., 2H), 7.43 (t like, ^3^*J* = 7.2 Hz, 2H), 7.52 (dt, ^3^*J* = 7.8 Hz, ^4^*J* = 1.2 Hz, 1H), 7.97 (d, ^3^*J* = 6.0 Hz, 1H), 8.05 (d, ^3^*J* = 8.0 Hz, 1H), 8.12 (d, ^3^*J* = 7.6 Hz, 1H), 11.08 (s, 1H), 13.83 ppm (br., 1H); ^13^C NMR (100 MHz; DMSO-*d*_6_) *δ*_C_ 20.53, 56.32, 71.15, 110.95, 115.37, 119.84, 120.11, 121.49, 122.05, 122.75, 125.41, 126.24, 126.45, 131.80, 132.40, 135.40, 138.68, 140.49, 144.81, 151.81, 152.35, 161.83, 162.48, 165.63 ppm; Anal. Calcd for C_25_H_20_N_4_O_4_S: C, 63.55; H, 4.27; N, 11.86. Found: C, 63.21; H, 4.49; N, 11.61.

##### 2-(2-(Benzo[*d*]thiazol-2-yl)-6-methoxyphenoxy)-*N*′-(5-methoxy-2-oxoindolin-3-ylidene)acetohydrazide (**9i**)

Pale brown powder; yield = 71%; mp 144–146 °C; IR (KBr) *ṽ* 3183, 3071, 3048, 3009, 2920, 2839, 1686, 1636, 1597, 1485 cm^−1^; ^1^H NMR (400 MHz; DMSO-*d*_6_) *δ*_H_ 3.79 (s, 3H), 3.87 (s, 3H), 4.87 (s, 2H), 6.85 (d, ^3^*J* = 8.4 Hz, 1H), 6.98 (d like, ^3^*J* = 6.8 Hz, 1H), 7.16 (s, 1H), 7.33 (br., 2H), 7.44 (dt, ^3^*J* = 7.4 Hz, ^4^* J* = 1.2 Hz 1H), 7.53 (dt, ^3^*J* = 7.8 Hz, ^4^* J* = 1.2 Hz, 1H), 7.98 (d, ^3^*J* = 5.6 Hz, 1H), 8.06 (d, ^3^*J* = 8.0 Hz, 1H), 8.13 (d, ^3^*J* = 7.6 Hz, 1H), 11.01 (s, 1H), 13.87 ppm (br., 1H); ^13^C NMR (100 MHz; DMSO-*d*_6_) *δ*_C_ 55.70, 56.36, 71.18, 105.96, 112.10, 115.45, 118.39, 120.11, 120.55, 122.10, 122.76, 125.45, 126.23, 126.50, 135.40, 136.42, 138.89, 144.82, 151.81, 152.35, 155.45, 162.55, 165.74 ppm; Anal. Calcd for C_25_H_20_N_4_O_5_S: C, 61.47; H, 4.13; N, 11.47. Found: C, 61.80; H, 4.38; N, 11.72.

##### 2-(2-(Benzo[*d*]thiazol-2-yl)-6-methoxyphenoxy)-*N*′-(5-nitro-2-oxoindolin-3-ylidene)acetohydrazide (**9j**)

Pale brown powder; yield = 72%; mp 276–278 °C; ^1^H NMR (400 MHz; DMSO-*d*_6_) *δ*_H_ 3.88 (s, 3H), 4.92 (s, 2H), 7.10 (d, ^3^*J* = 8.8 Hz, 1H), 7.32 (br., 2H), 7.42 (t, ^3^*J* = 7.6 Hz, 1H), 7.51 (t, ^3^*J* = 7.6 Hz, 1H), 7.94–7.97 (m, 1H), 8.03 (d, ^3^*J* = 8.0 Hz, 1H), 8.10 (d, ^3^*J* = 7.6 Hz, 1H), 8.27 (br., 2H), 11.81 (s, 1H), 13.66 ppm (br., 1H); ^13^C NMR (100 MHz; DMSO-*d*_6_) *δ*_C_ 56.34, 71.61, 111.43, 115.36, 116.04, 120.12, 122.02, 122.75, 125.41, 126.21, 126.45, 127.67, 135.43, 142.81, 147.81, 151.79, 152.30, 161.93, 162.70 ppm; Anal. Calcd for C_24_H_17_N_5_O_6_S: C, 57.25; H, 3.40; N, 13.91. Found: C, 57.51; H, 3.68; N, 13.69.

##### 2-(2-(Benzo[*d*]thiazol-2-yl)-6-methoxyphenoxy)-*N*′-(5-chloro-2-oxoindolin-3-ylidene)acetohydrazide (**9k**)

Pale brown powder; yield = 65%; mp 263–265 °C; IR (KBr) *ṽ* 3213, 3183, 3136, 3071, 3013, 2974, 2928, 1748, 1701, 1620, 1586, 1374 cm^−1^; ^1^H NMR (400 MHz; DMSO-*d*_6_) *δ*_H_ 3.87 (s, 3H), 4.89 (s, 1H), 5.39 (s, 1H), 6.94 (d, ^3^*J* = 8.4 Hz, 1H), 7.33 (br., 2H), 7.43 (dt, ^3^*J* = 7.6 Hz, ^4^*J* = 0.8 Hz, 2H), 7.52 (dt, ^3^*J* = 7.6 Hz, ^4^*J* = 1.2 Hz, 1H), 7.58 (br., 1H), 7.97 (d, ^3^*J* = 5.6 Hz, 1H), 8.05 (d, ^3^*J* = 8.0 Hz, 1H), 8.12 (d, ^3^*J* = 8.0 Hz, 1H), 11.31 (s, 1H), 13.79 ppm (br., 1H); ^13^C NMR (100 MHz; DMSO-*d*_6_) *δ*_C_ 56.36, 71.21, 109.14, 112.74, 115.44, 118.07, 120.12, 120.69, 122.08, 122.76, 125.43, 126.23, 126.48, 126.84, 131.31, 135.40, 141.44, 144.81, 151.84, 152.33, 162.24 ppm; Anal. Calcd for C_24_H_17_ClN_4_O_4_S: C, 58.48; H, 3.48; N, 11.37. Found: C, 58.23; H, 3.76; N, 11.51.

##### 2-(2-(Benzo[*d*]thiazol-2-yl)-6-methoxyphenoxy)-*N*′-(5-bromo-2-oxoindolin-3-ylidene)acetohydrazide (**9l**)

Yellow powder; yield = 78%; mp 266–268 °C; IR (KBr) *ṽ* 3213, 3179, 3136, 3071, 2974, 2932, 1744, 1701, 1616, 1586 cm^−1^; ^1^H NMR (400 MHz; DMSO-*d*_6_) *δ*_H_ 3.87 (s, 3H), 4.89 (s, 1H), 5.40 (s, 1H), 6.89 (d, ^3^*J* = 8.0 Hz, 1H), 7.32 (br., 2H), 7.43 (t, ^3^*J* = 7.2 Hz, 1H), 7.52 (t, ^3^*J* = 7.2 Hz, 1H), 7.54 (br., 1H), 7.69 (br., 1H), 7.97–7.98 (m, 1H), 8.05 (d, ^3^*J* = 8.4 Hz, 1H), 8.12 (d, ^3^*J* = 7.6 Hz, 1H), 11.31 (s, 1H), 13.78 ppm (s, 1H); ^13^C NMR (100 MHz; DMSO-*d*_6_) *δ*_C_ 56.33, 71.21, 113.14, 114.38, 115.39, 120.10, 122.04, 122.73, 123.37, 125.39, 126.21, 126.43, 134.15, 135.40, 141.77, 144.82, 151.78, 152.30, 162.05, 165.78 ppm; Anal. Calcd for C_24_H_17_BrN_4_O_4_S: C, 53.64; H, 3.19; N, 10.43. Found: C, 53.32; H, 3.42; N, 10.73.

##### 2-(5-(Benzo[*d*]thiazol-2-yl)-2-methoxyphenoxy)-*N*′-(2-oxoindolin-3-ylidene) acetohydrazide (**9m**)

Yellow powder; yield = 77%; mp 192–194 °C; IR (KBr) *ṽ* 3348, 3264, 3233, 3063, 3028, 2994, 2928, 1694, 1605, 1513, 1485, 1466 cm^−1^; ^1^H NMR (400 MHz; DMSO-*d*_6_) *δ*_H_ 3.93 (s, 3H), 5.00 (s, 2H), 6.95 (d, ^3^*J* = 7.6 Hz, 1H), 7.10 (t, ^3^*J* = 7.6 Hz, 1H), 7.22 (d, ^3^*J* = 7.6 Hz, 1H), 7.38–7.44 (m, 2H), 7.51 (t, ^3^*J* = 7.6 Hz, 1H), 7.59 (d, ^3^*J* = 6.8 Hz, 1H), 7.74 (br., 2H), 8.00 (d, ^3^*J* = 6.0 Hz, 1H), 8.10 (d, ^3^*J* = 7.2 Hz, 1H), 11.28 (s, 1H), 13.61 ppm (s, 1H); ^13^C NMR (100 MHz; DMSO-*d*_6_) *δ*_C_ 55.98, 71.12, 111.19, 112.73, 119.73, 121.08, 122.21, 122.52, 122.67, 125.21, 125.50, 126.57, 131.99, 134.33, 142.73, 153.56, 162.49, 166.96 ppm; Anal. Calcd for C_24_H_18_N_4_O_4_S: C, 62.87; H, 3.96; N, 12.22. Found: C, 62.51; H, 4.21; N, 12.54.

##### 2-(5-(Benzo[*d*]thiazol-2-yl)-2-methoxyphenoxy)-*N*′-(5-methyl-2-oxoindolin-3-ylidene)acetohydrazide (**9n**)

Pale brown powder; yield = 72%; mp 224–226 °C; ^1^H NMR (400 MHz; DMSO-*d*_6_) *δ*_H_ 2.29 (s, 3H), 3.92 (s, 3H), 4.99 (s, 2H), 6.83 (d, ^3^*J* = 7.6 Hz, 1H), 7.20 (t, ^3^*J* = 8.4 Hz, 2H), 7.40 (s, 1H), 7.43 (d, ^3^*J* = 7.6 Hz, 1H), 7.51 (t like, ^3^*J* = 6.8 Hz, 1H), 7.73 (br., 2H), 8.00 (br., 1H), 8.09 (d, ^3^*J* = 7.2 Hz, 1H), 11.17 (s, 1H), 13.60 ppm (s, 1H); ^13^C NMR (100 MHz; DMSO-*d*_6_) *δ*_C_ 20.48, 55.93, 68.16, 110.90, 112.63, 119.71, 121.37, 122.14, 122.49, 125.16, 125.48, 126.51, 131.71, 132.33, 134.33, 140.44, 153.55, 162.52, 166.90 ppm; Anal. Calcd for C_25_H_20_N_4_O_4_S: C, 63.55; H, 4.27; N, 11.86. Found: C, 63.76; H, 4.54; N, 11.61.

##### 2-(5-(Benzo[*d*]thiazol-2-yl)-2-methoxyphenoxy)-*N*′-(5-methoxy-2-oxoindolin-3-ylidene)acetohydrazide (**9o**)

Yellowish red powder; yield = 68%; mp 199–201 °C; IR (KBr) *ṽ* 3244, 3202, 3063, 2997, 2963, 1697, 1605, 1485, 1439 cm^−1^; ^1^H NMR (400 MHz; DMSO-*d*_6_) *δ*_H_ 3.76 (s, 3H), 3.92 (s, 3H), 5.00 (s, 1H), 5.43 (s, 1H), 6.85 (d, ^3^*J* = 8.0 Hz, 1H), 6.96 (d, ^3^*J* = 7.6 Hz, 1H), 7.12 (s, 1H), 7.21 (d, ^3^*J* = 8.4 Hz, 1H), 7.41 (t, ^3^*J* = 6.8 Hz, 1H), 7.50 (t, ^3^*J* = 6.8 Hz, 1H), 7.71 (br., 2H), 7.98–7.99 (m, 1H), 8.08 (d, ^3^*J* = 6.8 Hz, 1H), 11.08 (s, 1H), 13.66 ppm (br., 1H); ^13^C NMR (100 MHz; DMSO-*d*_6_) *δ*_C_ 55.69, 56.02, 71.19, 106.05, 112.11, 112.76, 118.43, 120.48, 122.23, 122.56, 125.28, 125.54, 126.64, 134.40, 136.47, 153.60, 155.45, 162.66, 167.01 ppm; Anal. Calcd for C_25_H_20_N_4_O_5_S: C, 61.47; H, 4.13; N, 11.47. Found: C, 61.69; H, 4.41; N, 11.29.

##### 2-(5-(Benzo[*d*]thiazol-2-yl)-2-methoxyphenoxy)-*N*′-(5-nitro-2-oxoindolin-3-ylidene)acetohydrazide (**9p**)

Pale brown powder; yield = 74%; mp 288–290 °C; ^1^H NMR (400 MHz; DMSO-*d*_6_) *δ*_H_ 3.93 (s, 3H), 5.09 (s, 1H), 5.50 (s, 1H), 7.12–7.22 (m, 2H), 7.42–7.51 (m, 2H), 7.71 (s, 2H), 7.99–8.09 (m, 2H), 8.29 (s, 2H), 11.90 (s, 1H), 13.43 ppm (s, 1H); Anal. Calcd for C_24_H_17_N_5_O_6_S: C, 57.25; H, 3.40; N, 13.91. Found: C, 57.53; H, 3.19; N, 13.65.

##### 2-(5-(Benzo[*d*]thiazol-2-yl)-2-methoxyphenoxy)-*N*′-(5-chloro-2-oxoindolin-3-ylidene)acetohydrazide (**9q**)

Yellowish brown powder; yield = 65%; mp 260–262 °C; ^1^H NMR (400 MHz; DMSO-*d*_6_) *δ*_H_ 3.92 (s, 3H), 5.02 (s, 1H), 5.44 (s, 1H), 6.94 (d, ^3^*J* = 6.8 Hz, 1H), 7.20 (d, ^3^*J* = 6.8 Hz, 1H), 7.42–7.54 (m, 4H), 7.71 (s, 2H), 7.99 (s, 1H), 8.08 (d, ^3^*J* = 6.0 Hz, 1H), 11.38 (s, 1H), 13.56 ppm (br., 1H); Anal. Calcd for C_24_H_17_ClN_4_O_4_S: C, 58.48; H, 3.48; N, 11.37. Found: C, 58.30; H, 3.71; N, 11.60.

##### 2-(5-(Benzo[*d*]thiazol-2-yl)-2-methoxyphenoxy)-*N*′-(5-bromo-2-oxoindolin-3-ylidene)acetohydrazide (**9r**)

Pale brown powder; yield = 72%; mp 271–273 °C; ^1^H NMR (400 MHz; DMSO-*d*_6_) *δ*_H_ 3.92 (s, 3H), 5.01 (s, 1H), 5.44 (s, 1H), 6.88 (d, ^3^*J* = 6.4 Hz, 1H), 7.20 (d, ^3^*J* = 6.4 Hz, 1H), 7.41 (t like, ^3^*J* = 5.6 Hz, 1H), 7.52 (t like, ^3^*J* = 7.2 Hz, 2H), 7.69–7.71 (m, 3H), 7.98 (s, 1H), 8.07 (d, ^3^*J* = 5.6 Hz, 1H), 11.38 (s, 1H), 13.54 ppm (br., 1H); Anal. Calcd for C_24_H_17_BrN_4_O_4_S: C, 53.64; H, 3.19; N, 10.43. Found: C, 53.91; H, 3.01; N, 10.77.

### Biology

#### Cell cycle analysis and apoptosis assay on DU145 from prostate cancer

DU145 cancer cell line derived from prostate cancer was treated with the oxindole–benzothiazole conjugate **9o** at its GI_50_. This was followed by treatment of cells according to the reported procedure and the percentage of cells in each stage of the cell cycle was identified and the percentage of cells in the apoptotic and necrotic stages were detected [41, 42] (for further details see additional file 1: analysis of cell cycle distribution; apoptosis assay).

#### Screening of the inhibitory activity of oxindole–benzothiazole hybrids 9b, 9f and 9o on CDK2

The oxindole–benzothiazole hybrids **9b, 9f** and **9o** were investigated for their potency to suppress the activity of CDK2 employing CDK2 assay kit (BPS Biosciences—San Diego—CA—US) following the protocol of the manufacturer (for further details see Additional file 1: biochemical kinase assay procedure).

## Supplementary Information

Supplementary Material 1. (1) NMR Spectra of oxindole–benzothiazole hybrids **9a–r**. (2) IR charts of the synthesized oxindole–benzothiazoles. (3) Screening of cytotoxic activity against a panel of sixty human tumor cell lines. (4) One dose mean graphs of the oxindole–benzothiazoles. (5) Dose response curve of **9o** on NCI cancer cell lines. (6) Analysis of cell cycle distribution. (7) Apoptosis assay. (8) Biochemical kinase assay procedure. (9) Docking of the co-crystalized ligand in the binding site of CDK2. (10) Bioavailability radar charts for **9a–r** from SwissADME free webtool. (11) References.

## Data Availability

The datasets used and/or analyzed during the current study are available from the corresponding author on reasonable request.
