# Supplementary material for: Oxindole–benzothiazole hybrids as CDK2 inhibitors and anticancer agents: design, synthesis and biological evaluation
Source: BMC Chem. 2024 Sep 13;18(1):169. doi: 10.1186/s13065-024-01277-1 (PMC11396129; doi:10.1186/s13065-024-01277-1)
Supplement: Supplementary file 1 — Supplementary Material 1. (1) NMR Spectra of oxindole–benzothiazole hybrids 9a–r. (2) IR charts of the synthesized oxindole–benzothiazoles. (3) Screening of cytotoxic activity against a panel of sixty human tumor cell lines. (4) One dose mean graphs of the oxindole–benzothiazoles. (5) Dose response curve of 9o on NCI cancer cell lines. (6) Analysis of cell cycle distribution. (7) Apoptosis assay. (8) Biochemical kinase assay procedure. (9) Docking of the co-crystalized ligand in the binding site of CDK2. (10) Bioavailability radar charts for 9a–r from SwissADME free webtool. (11) References. [file 13065_2024_1277_MOESM1_ESM.pdf]

Supporting Information

Oxindole-benzothiazole hybrids as CDK2 inhibitors and anticancer agents: design, synthesis and biological evaluation

Heba T. Abdel-Mohsen[1]*

[1]Chemistry of Natural and Microbial Products Department, Pharmaceutical and Drug Industries Research Institute, National Research Centre, Dokki, P.O. 12622, Cairo, Egypt.

* Corresponding author. Email address: ht.abdel-mohsen@nrc.sci.eg and hebabdelmohsen@gmail.com (Heba T. Abdel-Mohsen)

Contents **Page**

1. **NMR Spectra of oxindole-benzothiazole hybrids 9a-r** 2
2. **IR charts of the synthesized oxindole-benzothiazoles** 35
3. **Screening of cytotoxic activity against a panel of sixty human tumor cell lines** 48
4. **One-dose mean graphs of the oxindole-benzothiazoles** 50
5. **Dose response curve of 9o on NCI cancer cell lines** 67
6. **Analysis of cell cycle distribution** 68
7. **Apoptosis assay** 68
8. **Biochemical kinase assay procedure** 69
9. **Docking of the co-crystalized ligand in the binding site of CDK2** 70
10. **Bioavailability radar charts for 9a-r from SwissADME free webtool** 72
11. **References** 73

1. NMR Spectra of oxindole-benzothiazole hybrids 9a-r

2-(2-(Benzo[*d*]thiazol-2-yl)phenoxy)-*N'*-(2-oxoindolin-3-ylidene)acetohydrazide (**9a**)

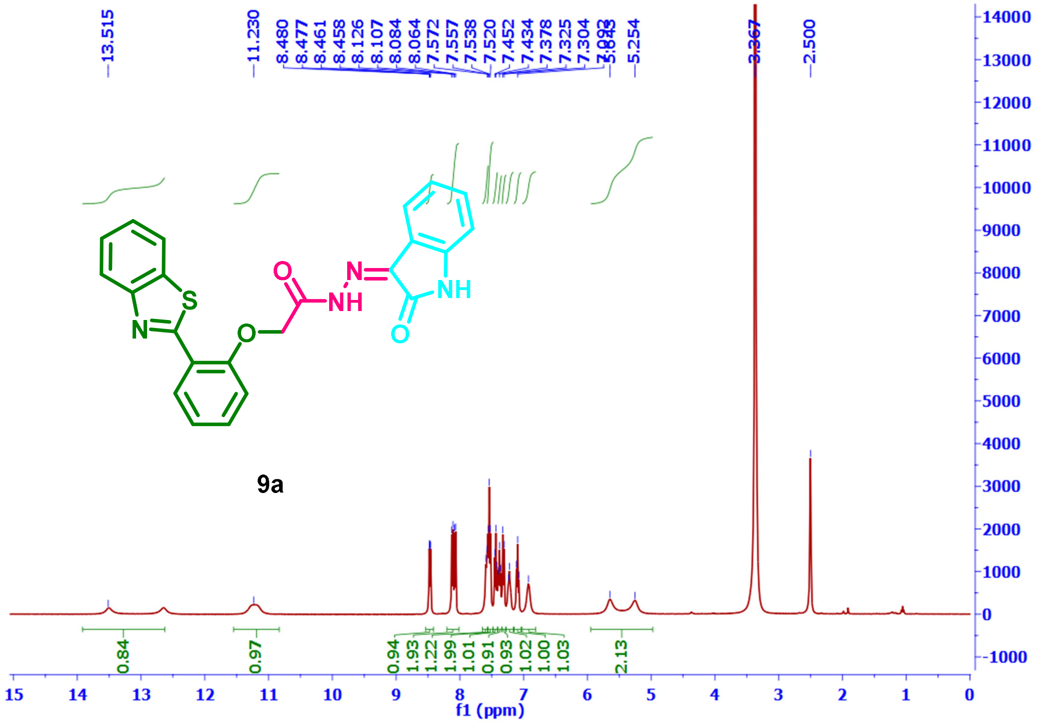

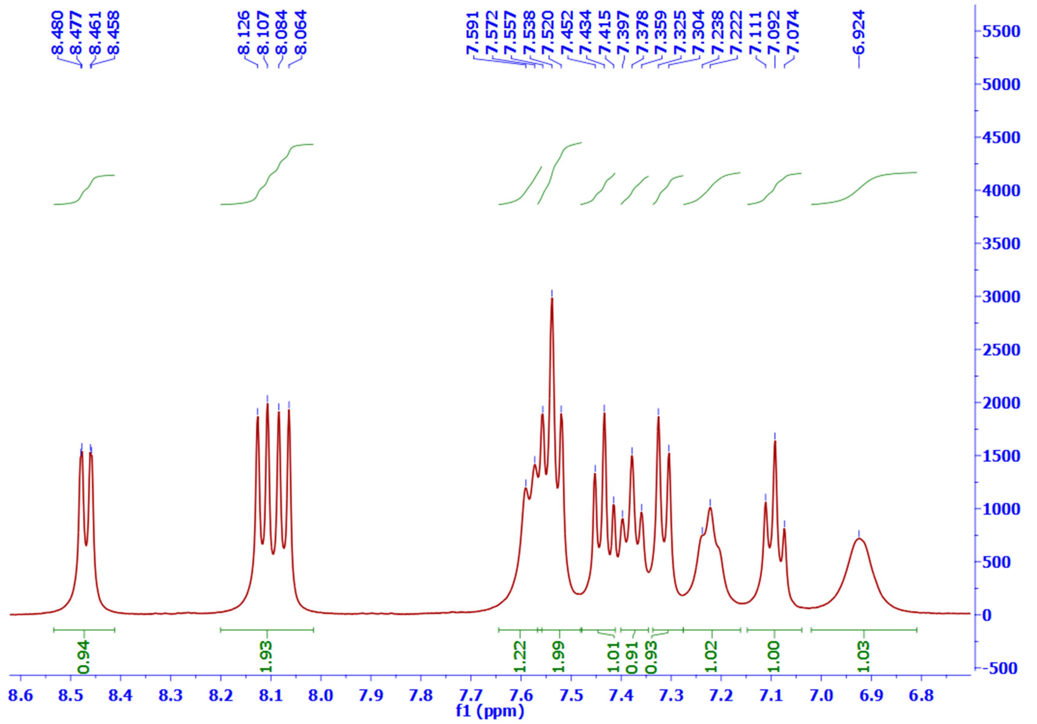

Figure 1. ^{1}H (400 MHz) NMR spectrum of **9a** in DMSO-d_6

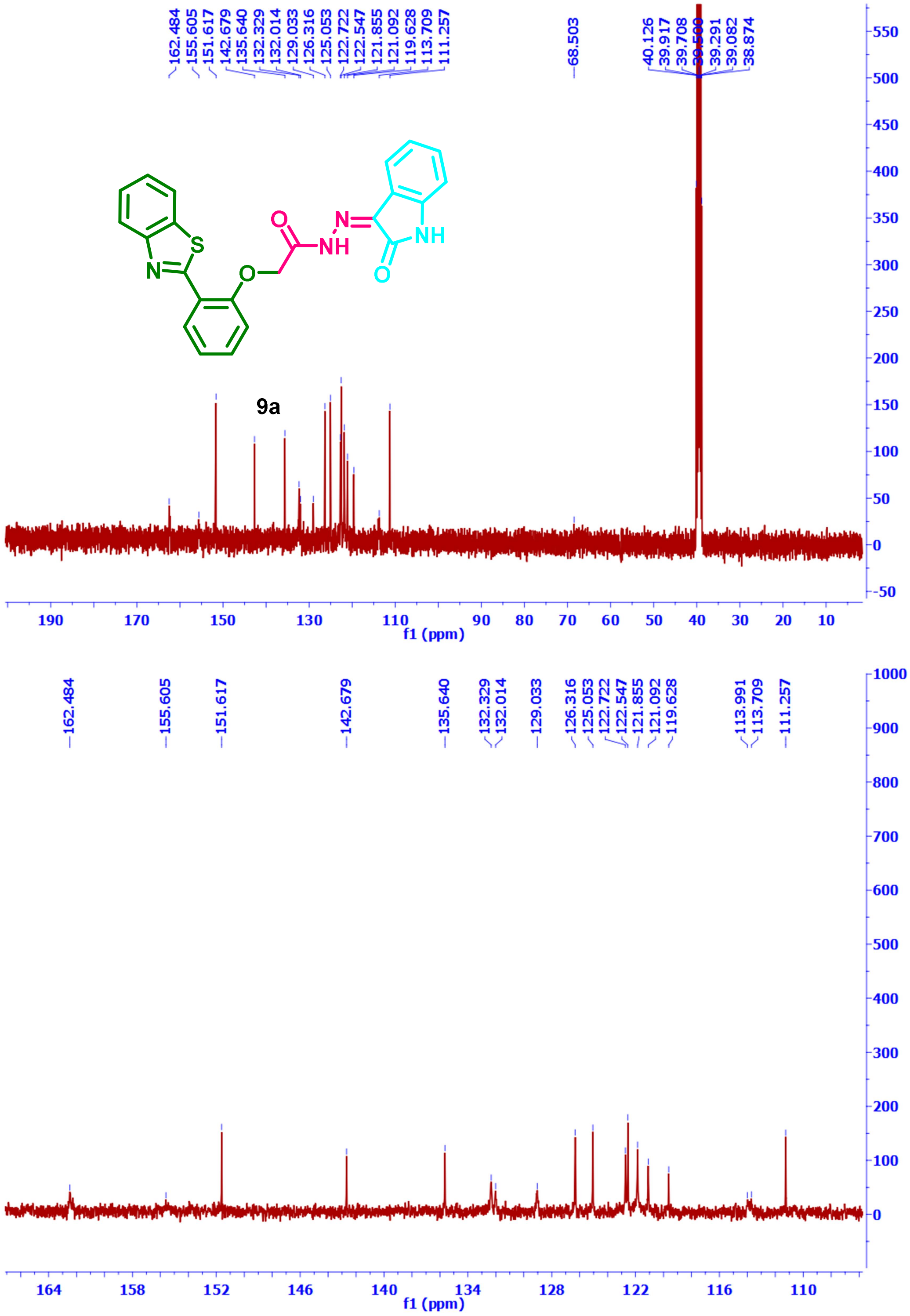

Figure 2. ^{13}C (100 MHz) NMR spectrum of **9a** in DMSO-d_6

2-(2-(Benzo[*d*]thiazol-2-yl)phenoxy)-*N*'-(5-methyl-2-oxoindolin-3-ylidene)acetohydrazide (**9b**)

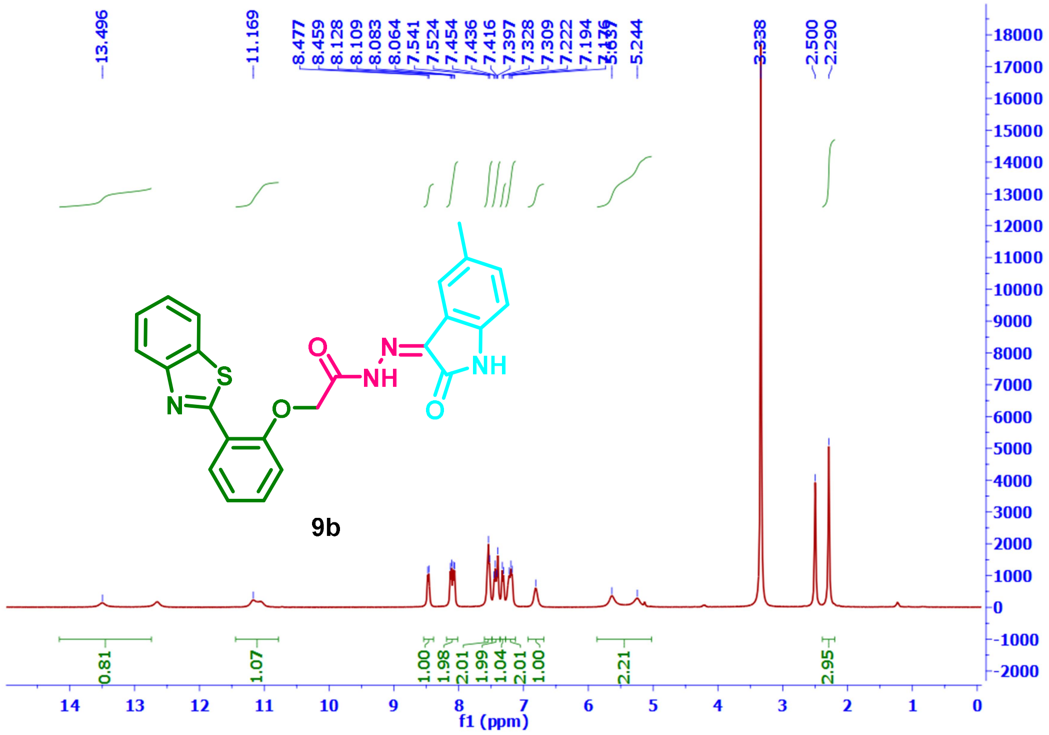

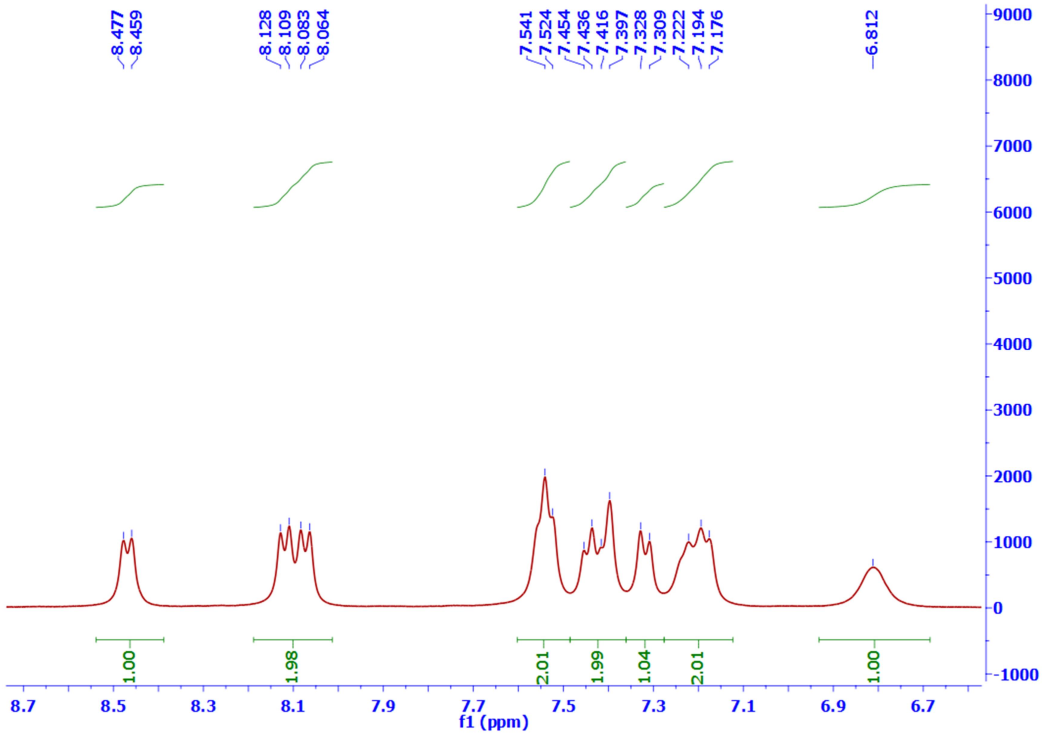

Figure 3. ^{1}H (400 MHz) NMR spectrum of **9b** in DMSO-d_6

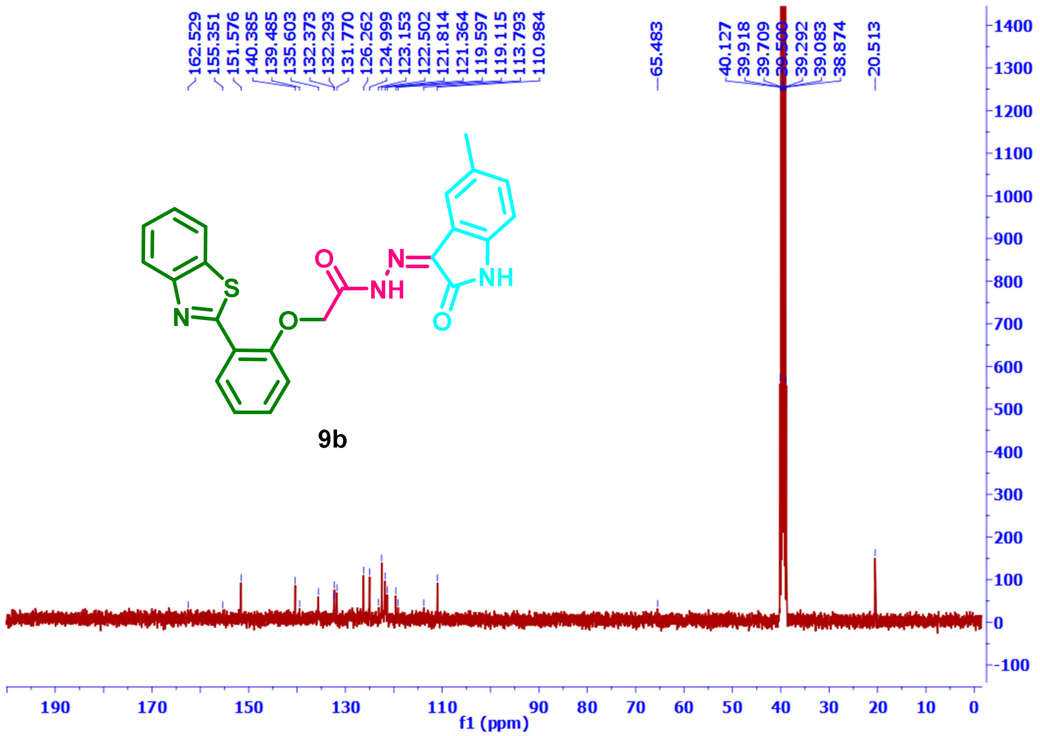

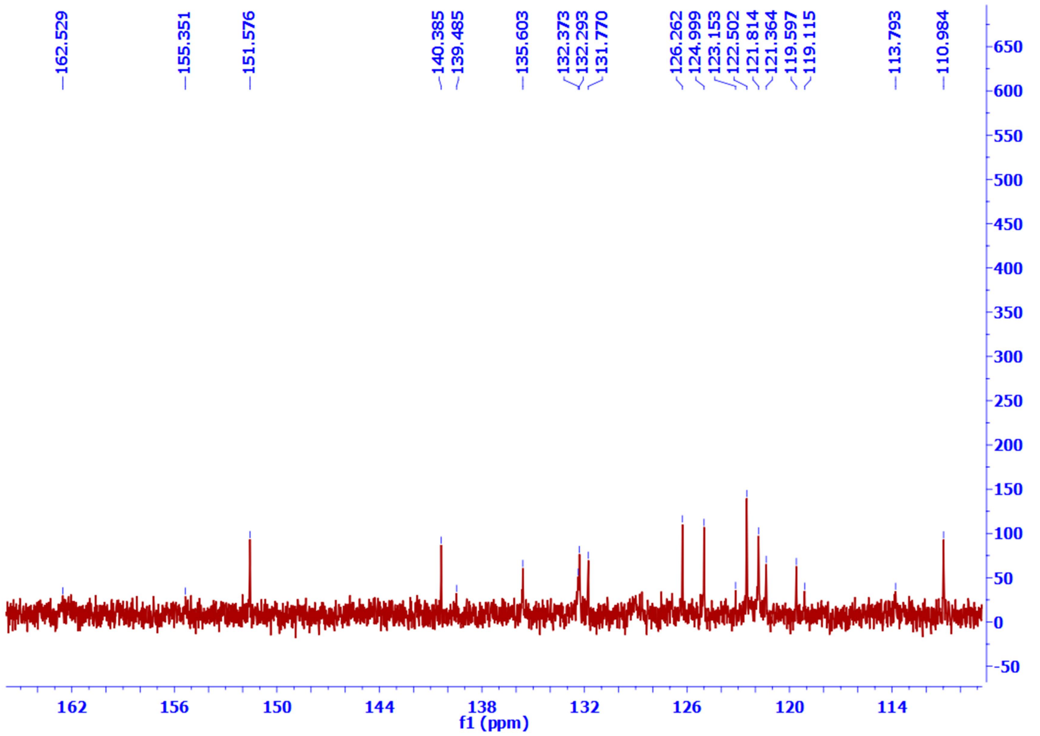

Figure 4. ^{13}C (100 MHz) NMR spectrum of **9b** in DMSO-d_6

2-(2-(Benzo[*d*]thiazol-2-yl)phenoxy)-*N*'-(5-methoxy-2-oxoindolin-3-ylidene)acetohydrazide (**9c**)

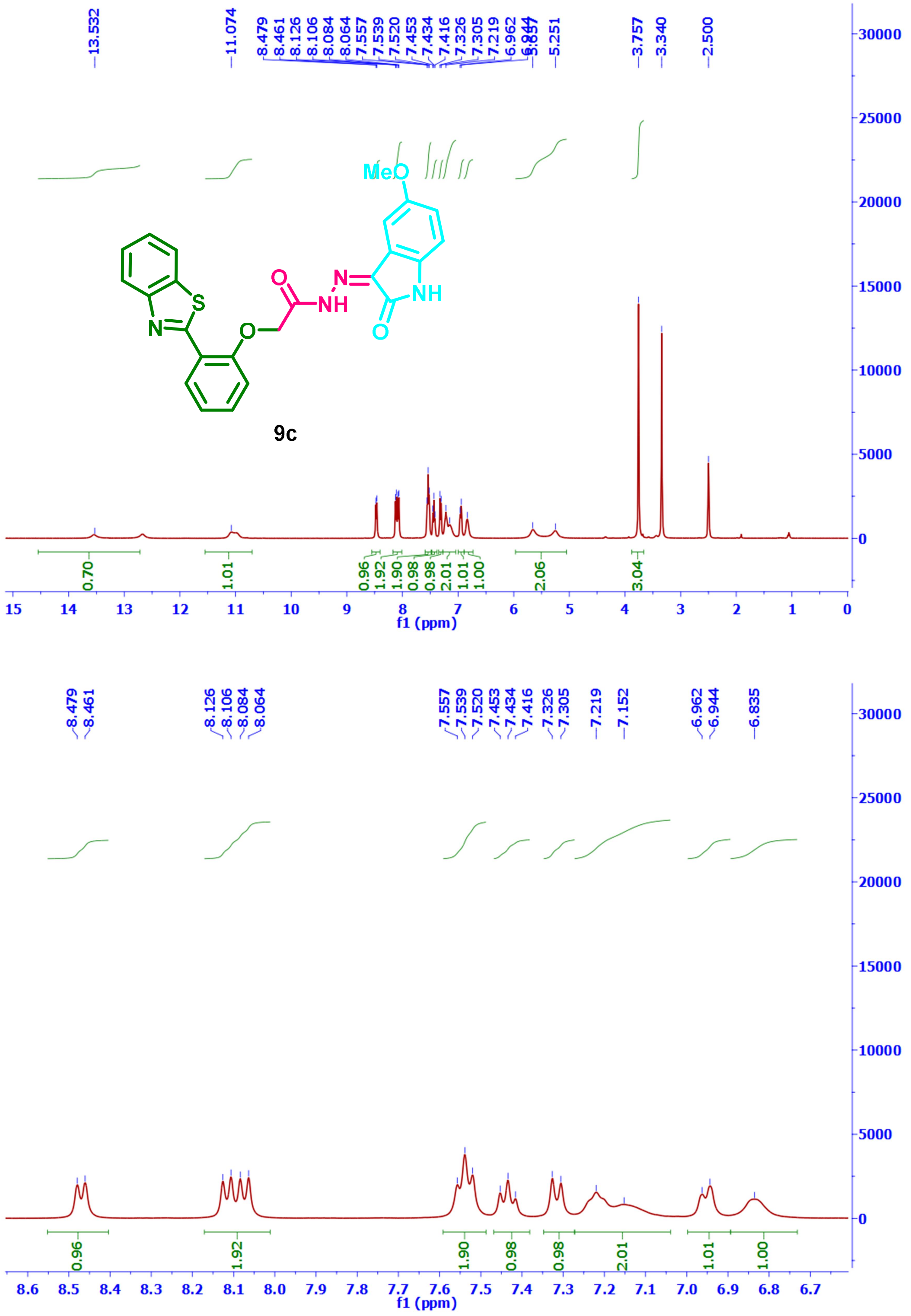

Figure 5. ^{1}H (400 MHz) NMR spectrum of **9c** in DMSO-d_6

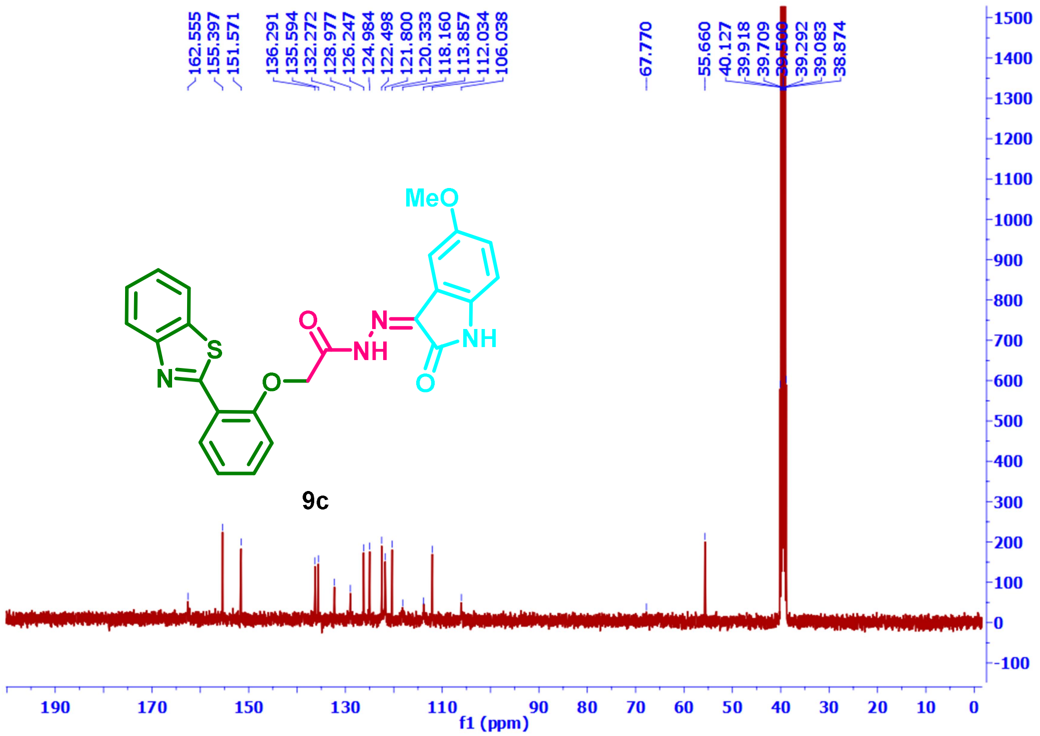

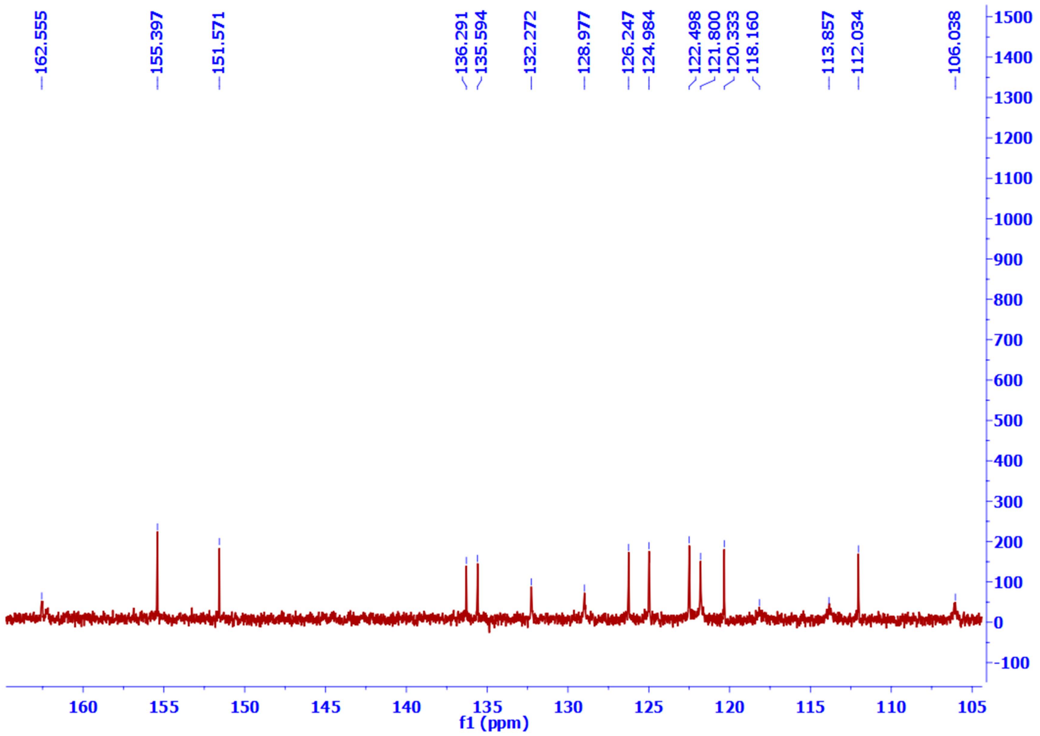

Figure 6. ^{13}C (100 MHz) NMR spectrum of **9c** in DMSO-d_6

2-(2-(Benzo[*d*]thiazol-2-yl)phenoxy)-*N*'-(5-nitro-2-oxoindolin-3-ylidene)acetohydrazide (**9d**)

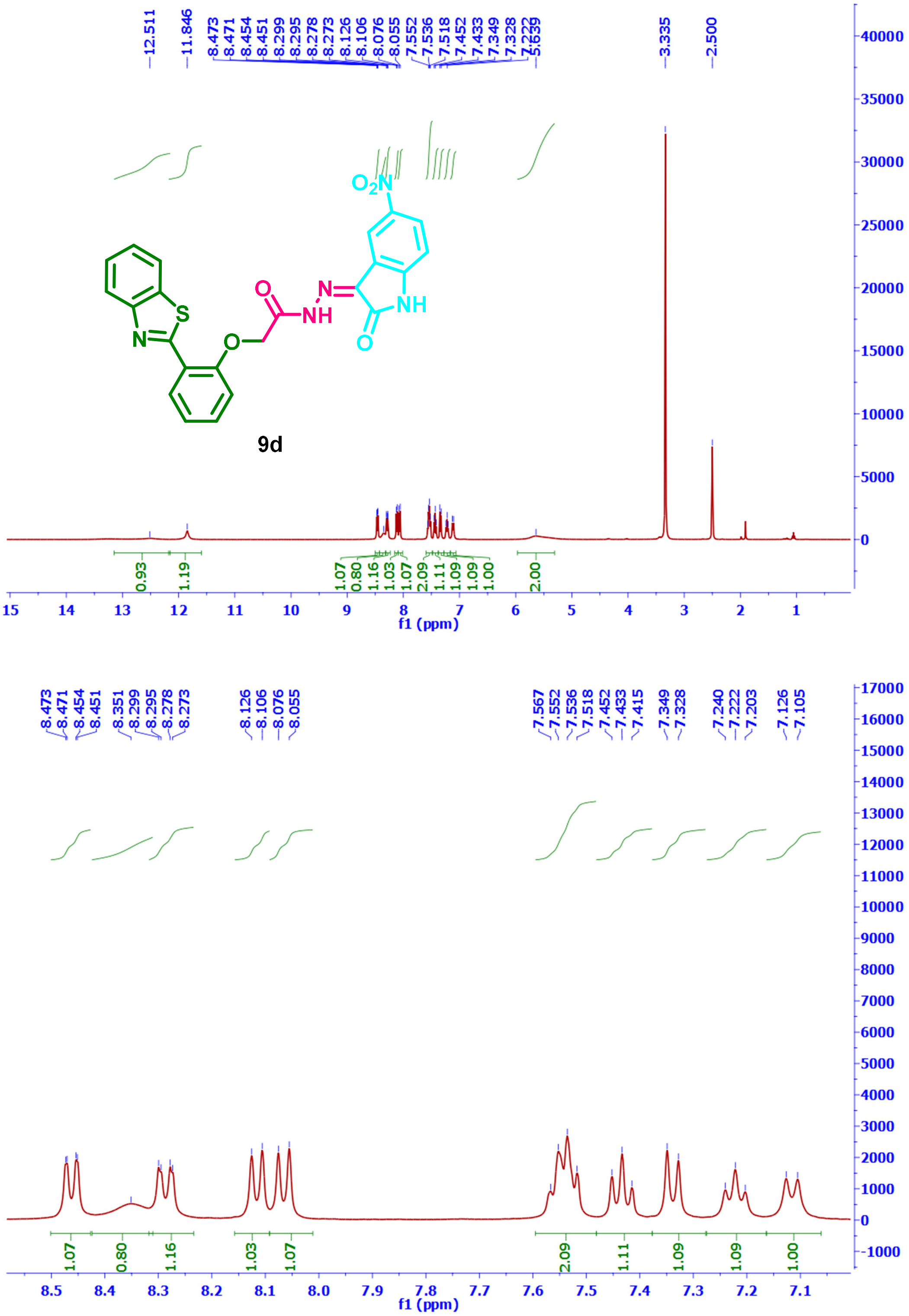

Figure 7. ^{1}H (400 MHz) NMR spectrum of **9d** in DMSO-d_6

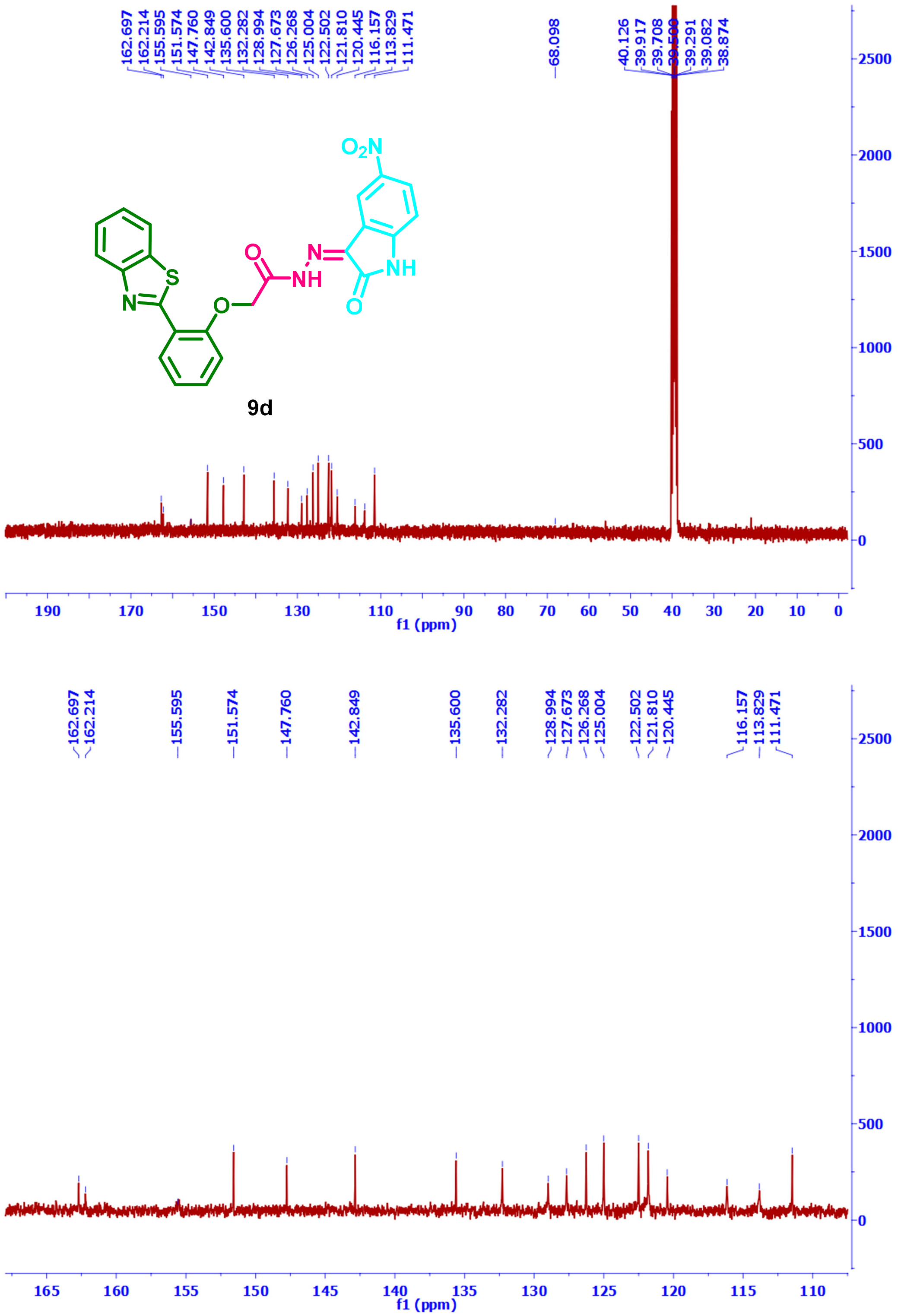

Figure 8. ^{13}C (100 MHz) NMR spectrum of **9d** in DMSO-d_6

2-(2-(Benzo[*d*]thiazol-2-yl)phenoxy)-*N*'-(5-chloro-2-oxoindolin-3-ylidene)acetohydrazide (**9e**)

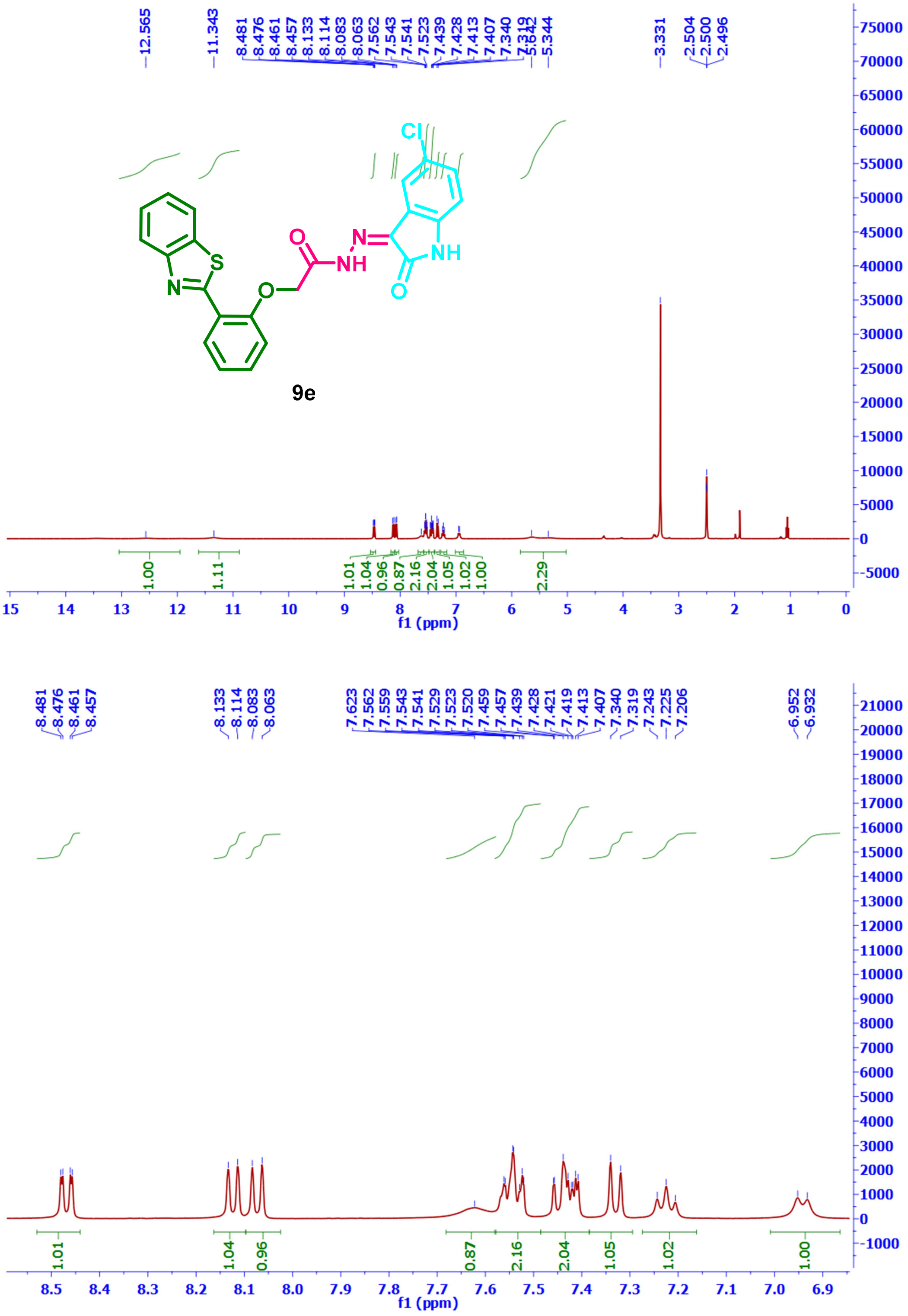

Figure 9. ^{1}H (400 MHz) NMR spectrum of **9e** in DMSO-d_6

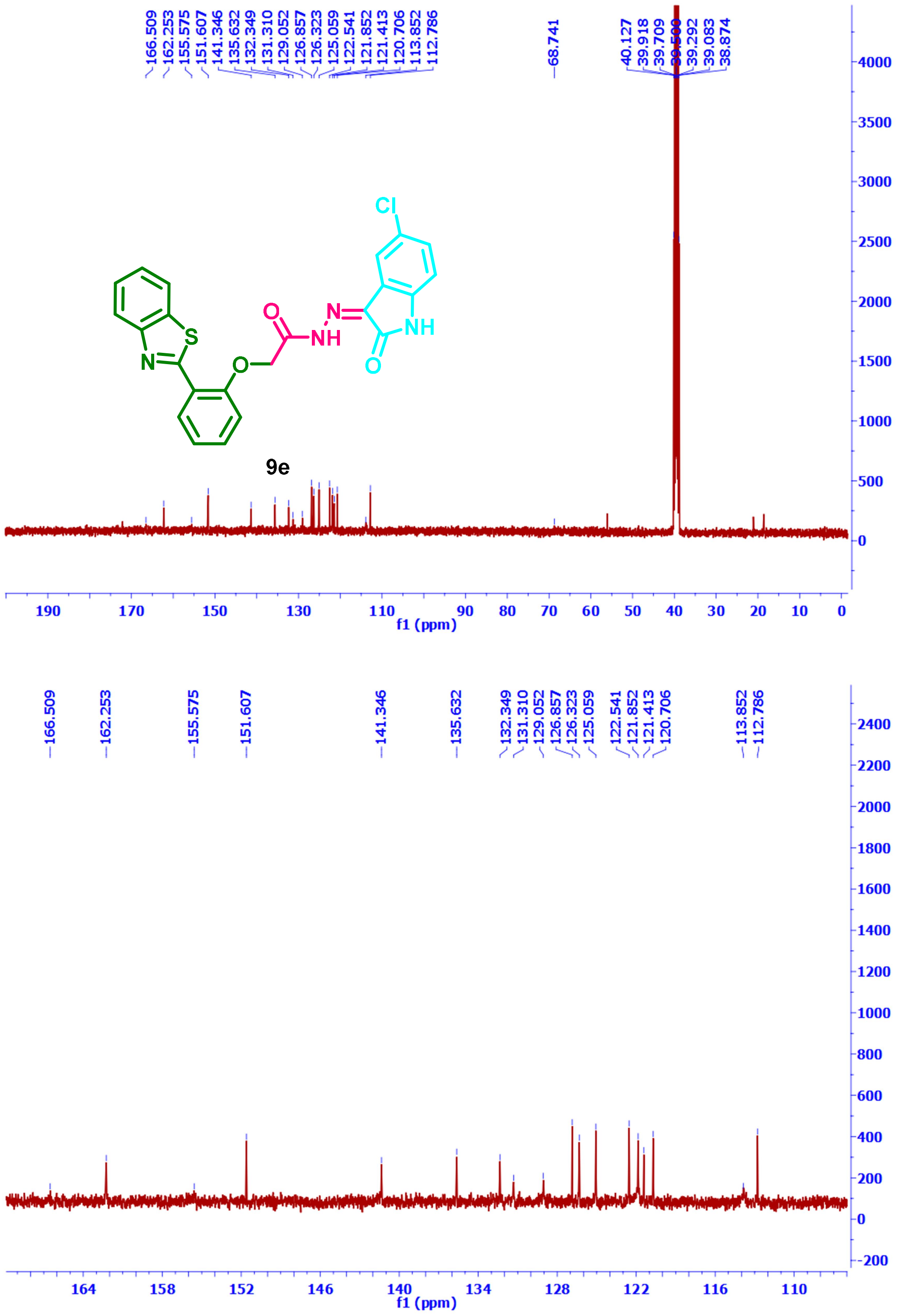

Figure 10. ^{13}C (100 MHz) NMR spectrum of **9e** in DMSO-d_6

2-(2-(Benzo[*d*]thiazol-2-yl)phenoxy)-*N*'-(5-bromo-2-oxoindolin-3-ylidene)acetohydrazide (**9f**)

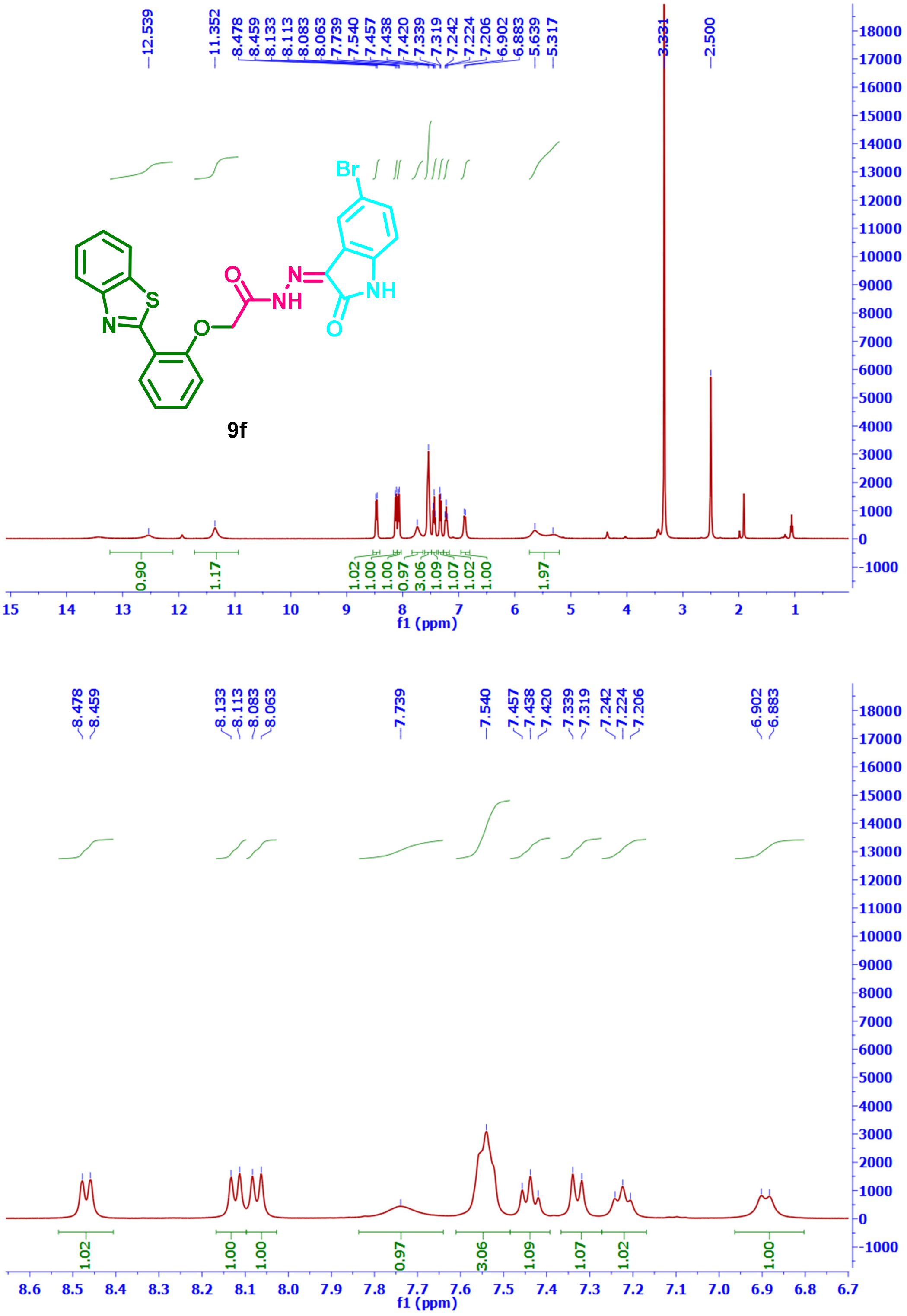

Figure 11. ^{1}H (400 MHz) NMR spectrum of **9f** in DMSO-d_6

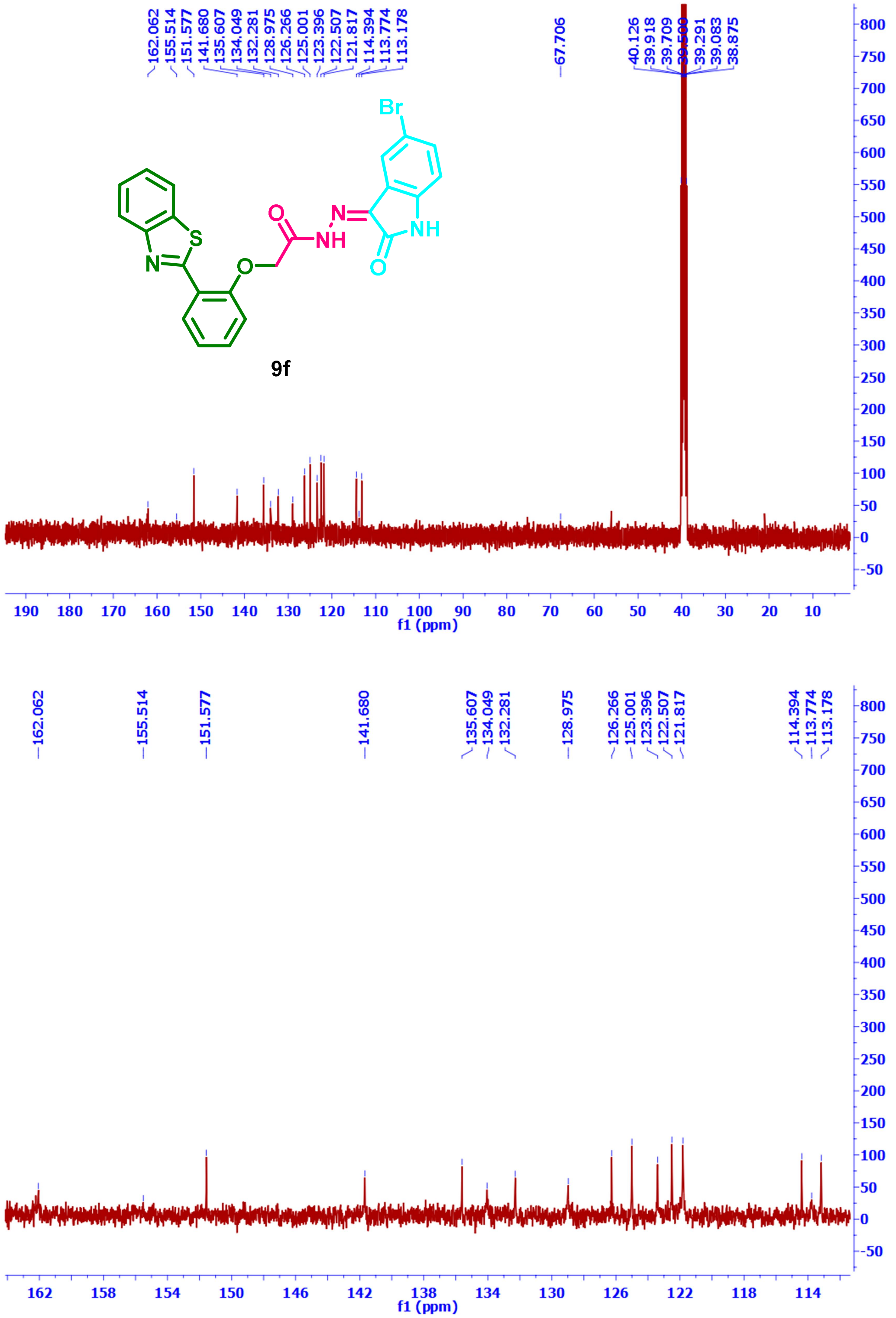

Figure 12. ^{13}C (100 MHz) NMR spectrum of **9f** in DMSO-d_6

2-(2-(Benzo[*d*]thiazol-2-yl)-6-methoxyphenoxy)-*N*'-(2-oxoindolin-3-ylidene)acetohydrazide (**9g**)

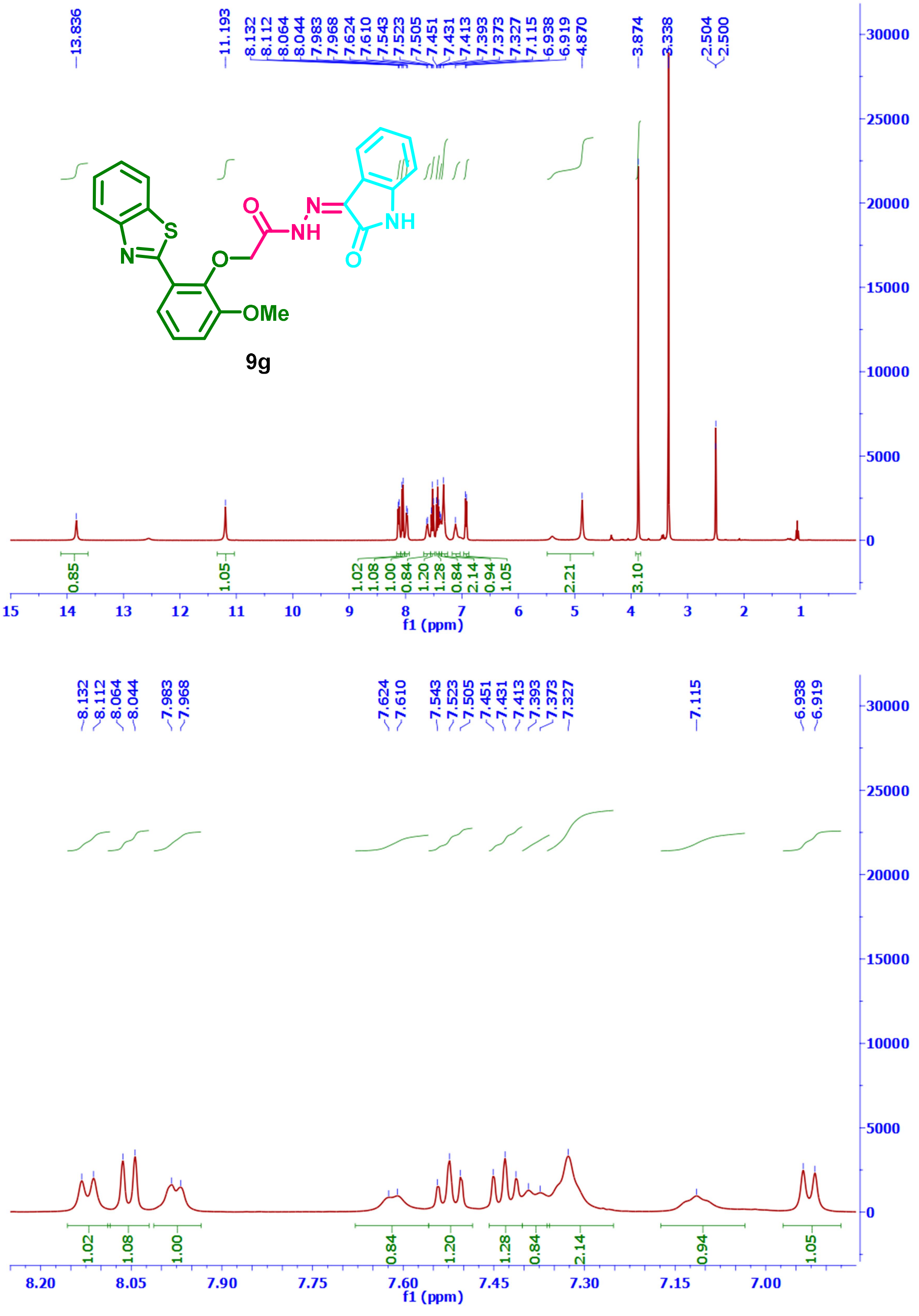

Figure 13. ^{1}H (400 MHz) NMR spectrum of **9g** in DMSO-d_6

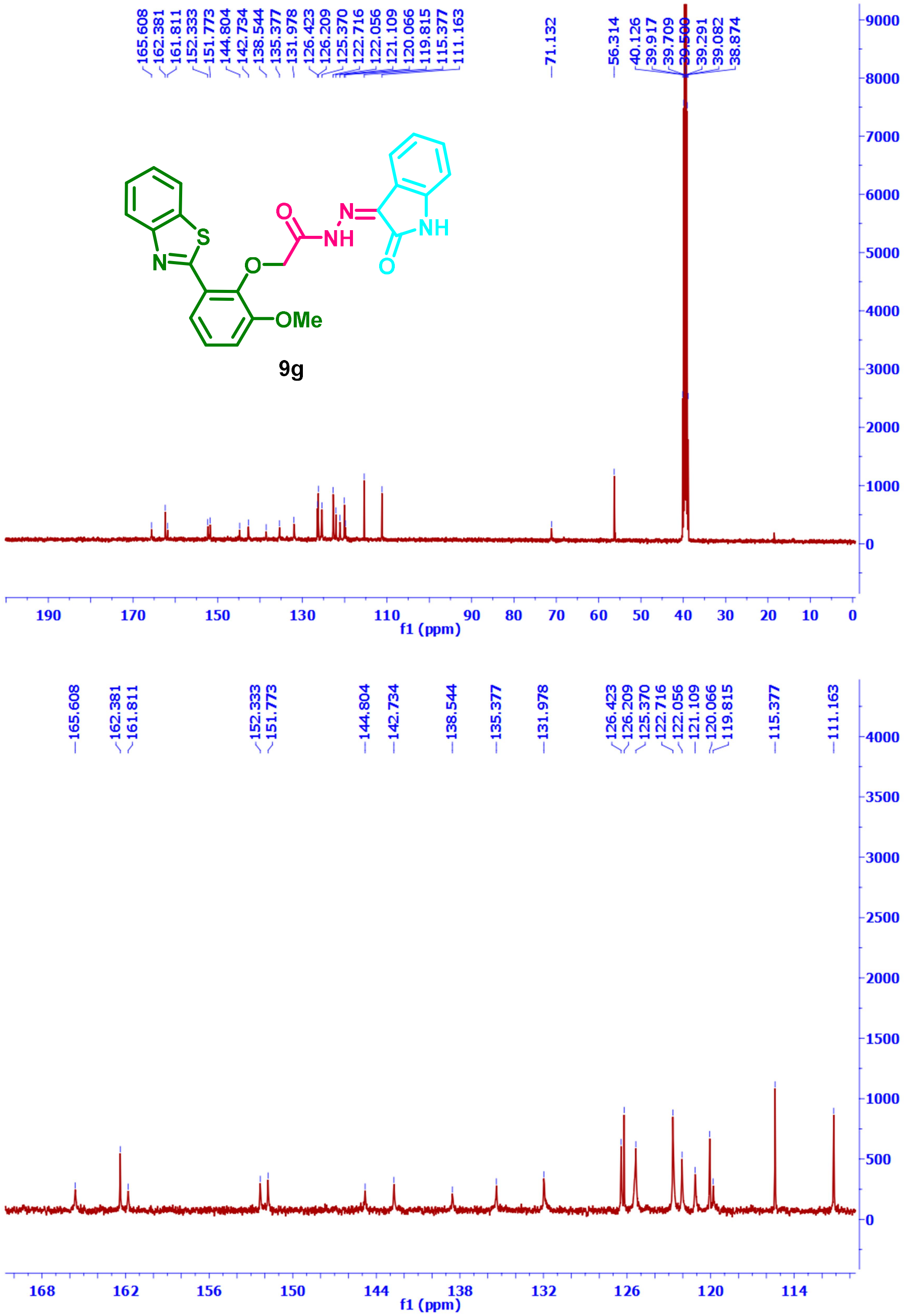

Figure 14. ^{13}C (100 MHz) NMR spectrum of **9g** in DMSO-d_6

2-(2-(Benzo[*d*]thiazol-2-yl)-6-methoxyphenoxy)-*N'*-(5-methyl-2-oxoindolin-3-ylidene)acetohydrazide (**9h**)

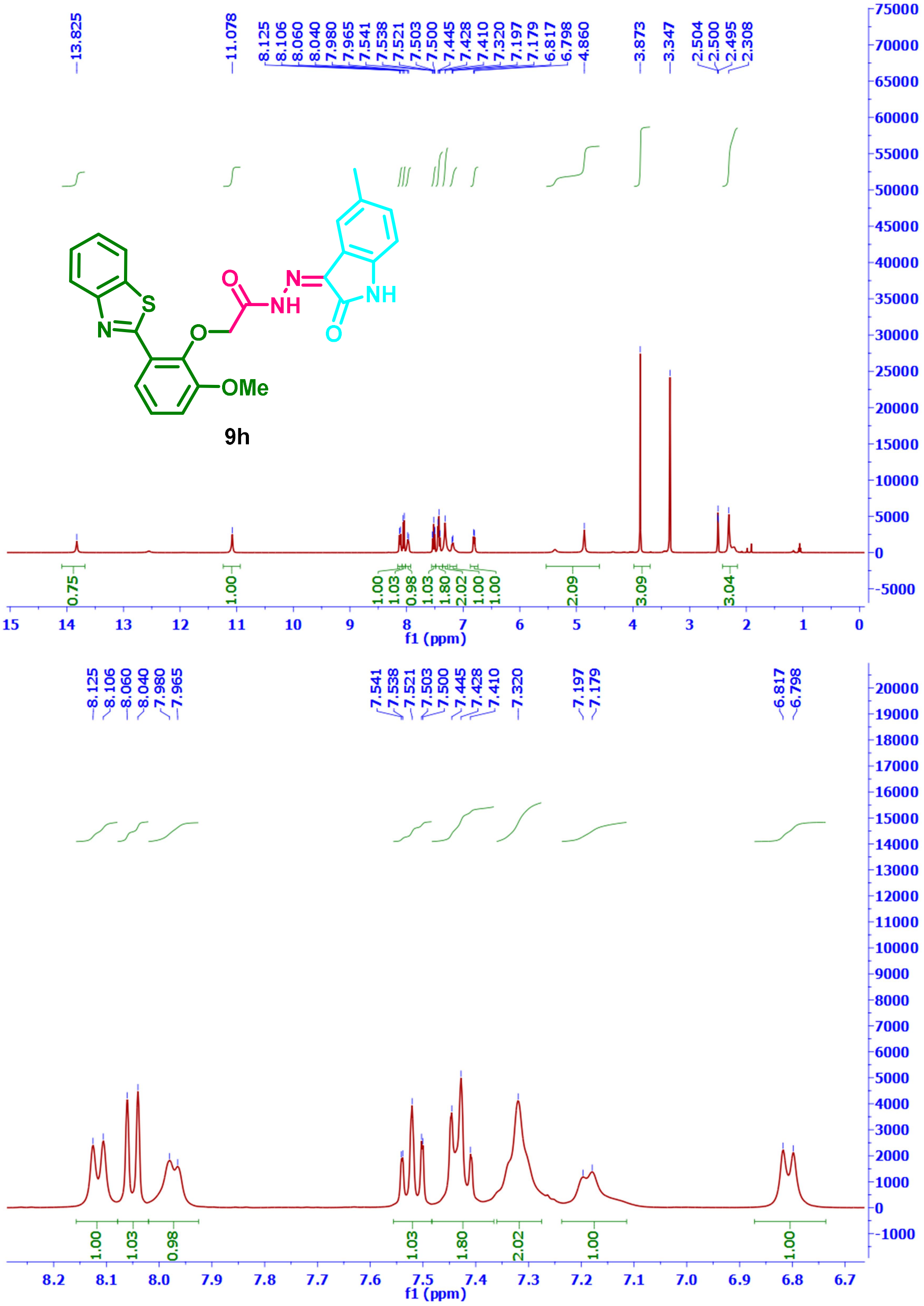

Figure 15. ^{1}H (400 MHz) NMR spectrum of **9h** in DMSO-d_6

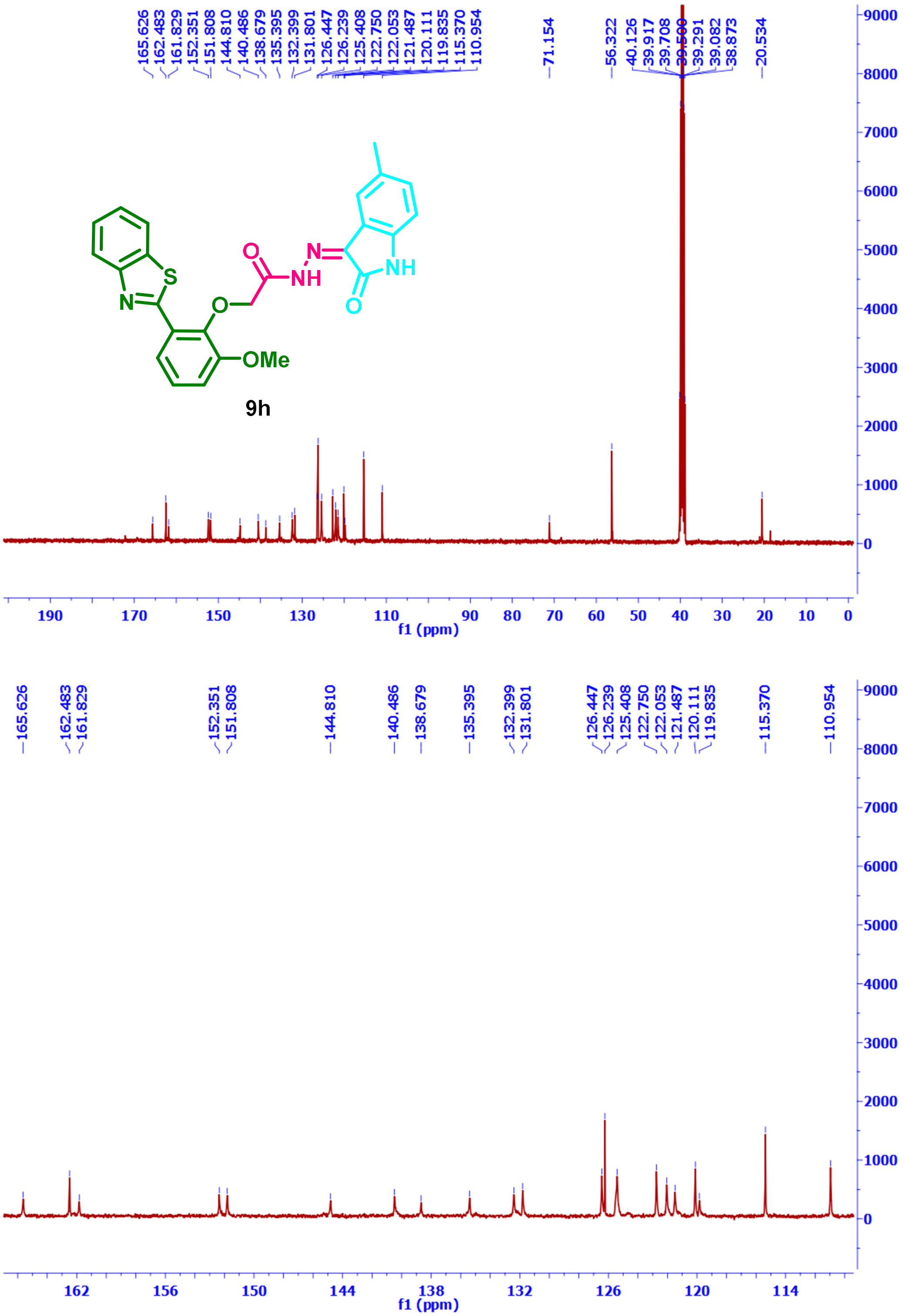

Figure 16. ^{13}C (100 MHz) NMR spectrum of **9h** in DMSO-d_6

2-(2-(Benzo[*d*]thiazol-2-yl)-6-methoxyphenoxy)-*N*'-(5-methoxy-2-oxoindolin-3-ylidene)acetohydrazide (**9i**)

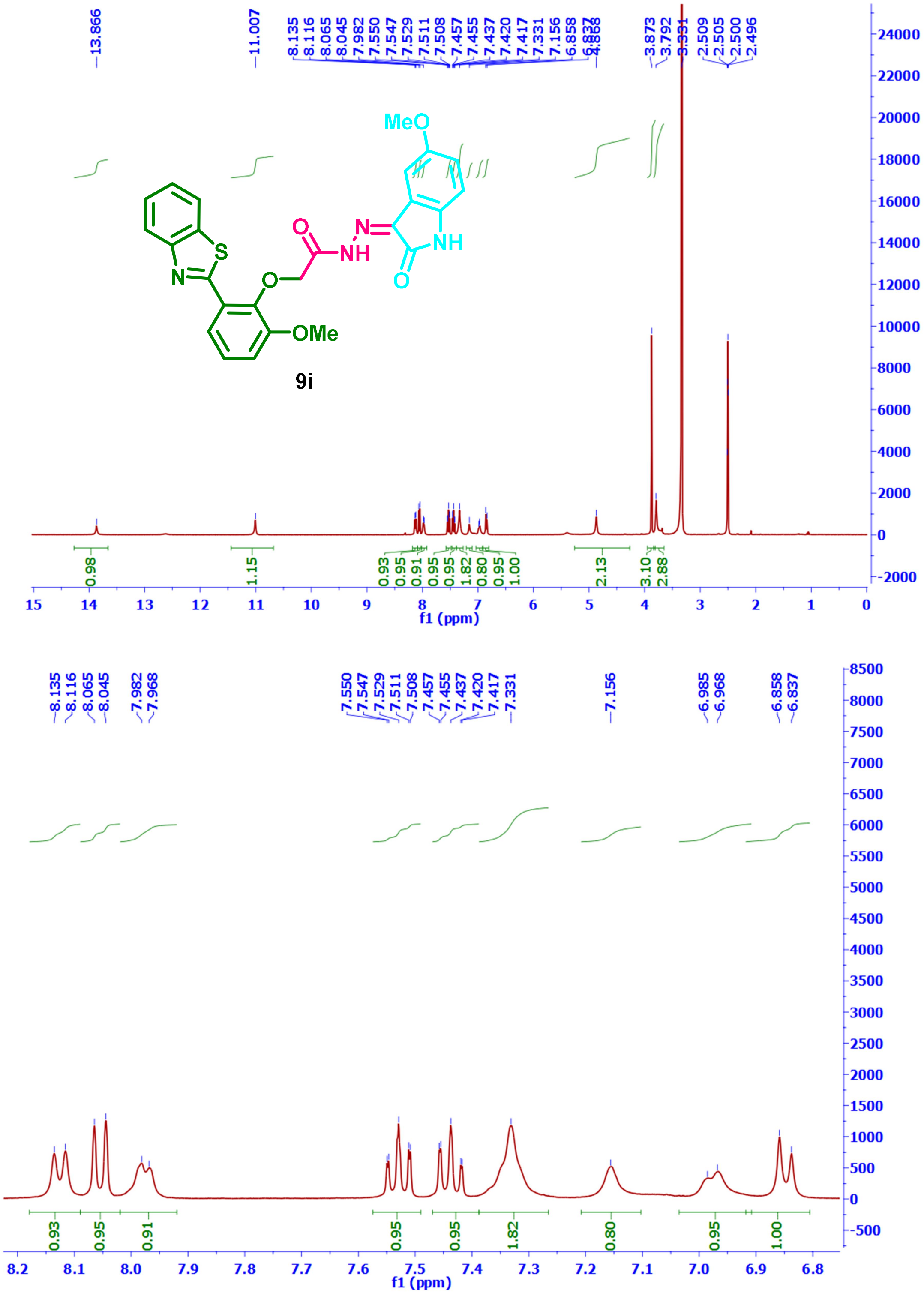

Figure 17. ^{1}H (400 MHz) NMR spectrum of **9i** in DMSO-d_6

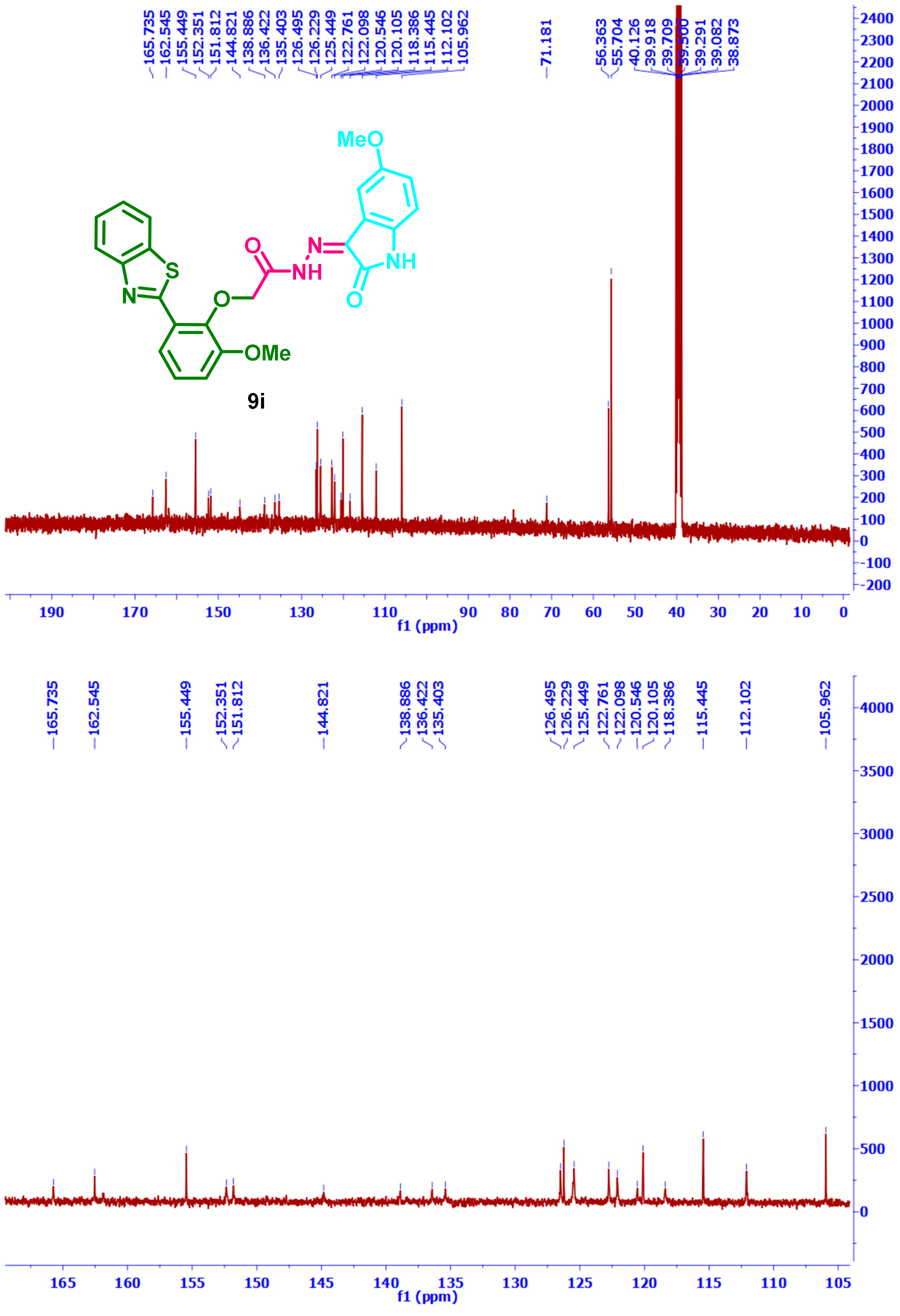

Figure 18. ^{13}C (100 MHz) NMR spectrum of **9i** in DMSO-d_6

2-(2-(Benzo[*d*]thiazol-2-yl)-6-methoxyphenoxy)-*N*'-(5-nitro-2-oxoindolin-3-ylidene)acetohydrazide (**9j**)

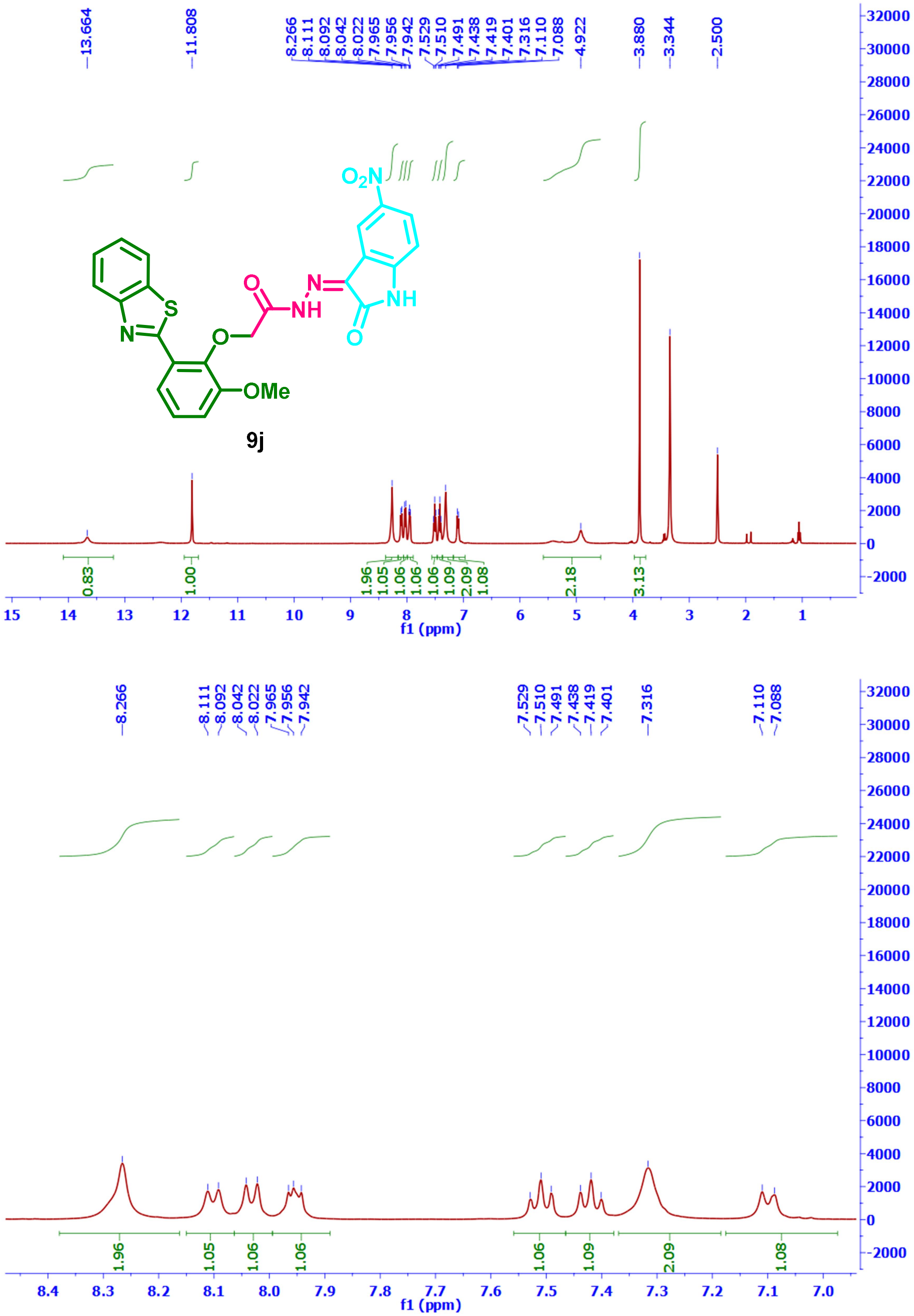

Figure 19. ^{1}H (400 MHz) NMR spectrum of **9j** in DMSO-d_6

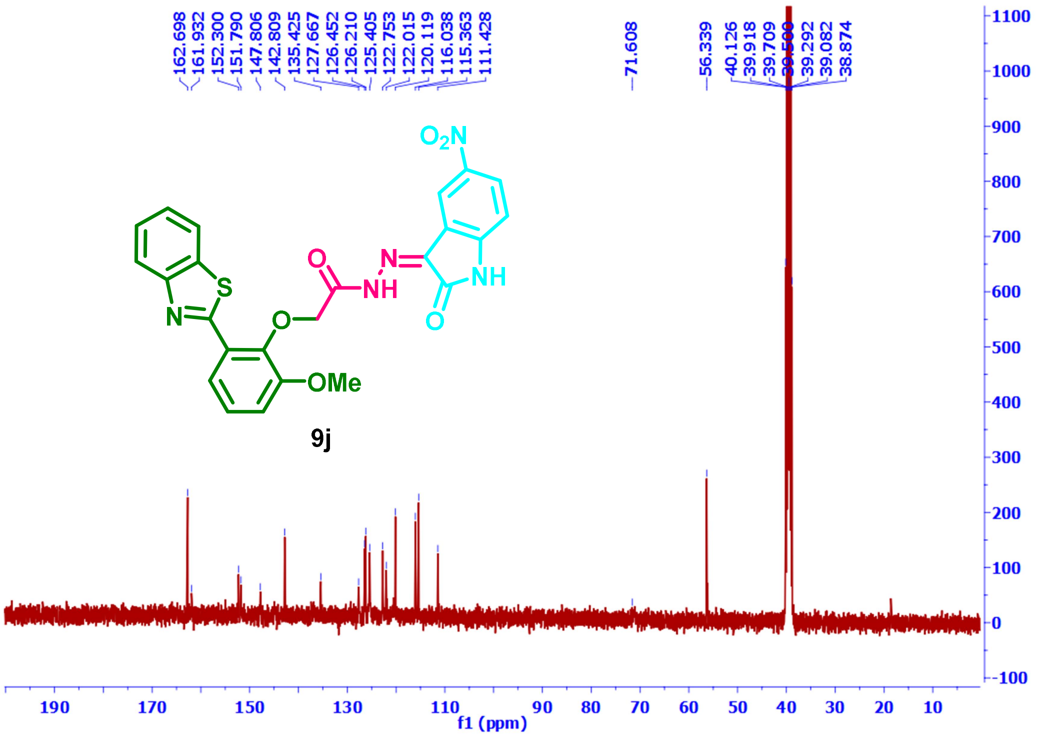

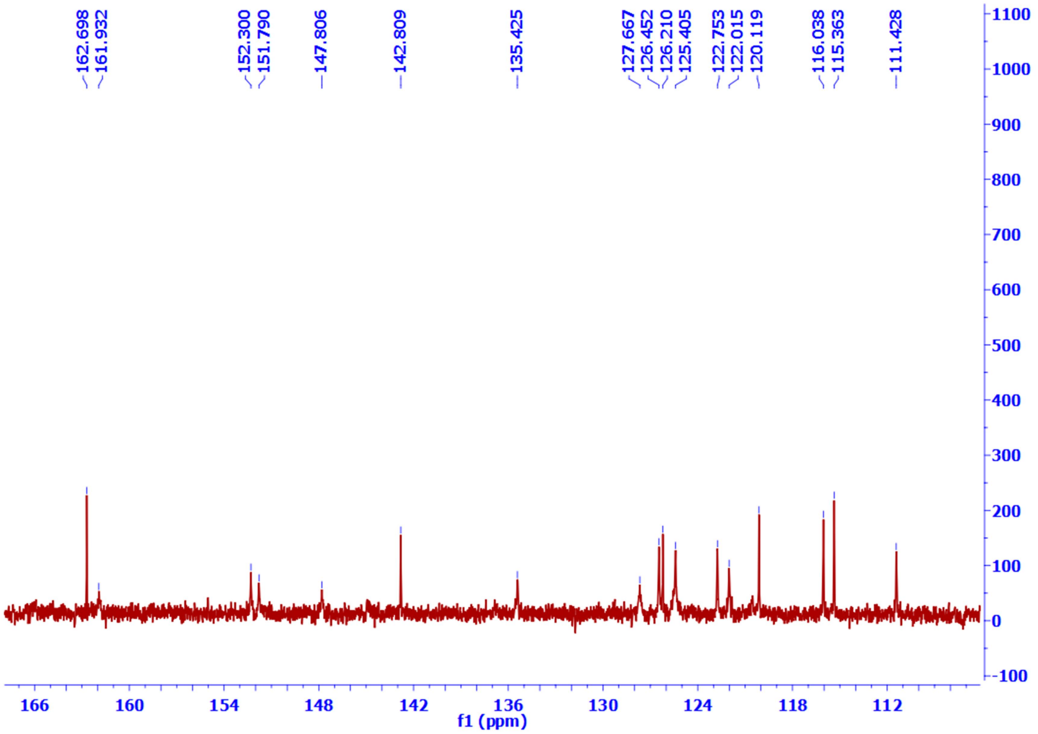

Figure 20. ^{13}C (100 MHz) NMR spectrum of **9j** in DMSO-d_6

2-(2-(Benzo[*d*]thiazol-2-yl)-6-methoxyphenoxy)-*N'*-(5-chloro-2-oxoindolin-3-ylidene)acetohydrazide (**9k**)

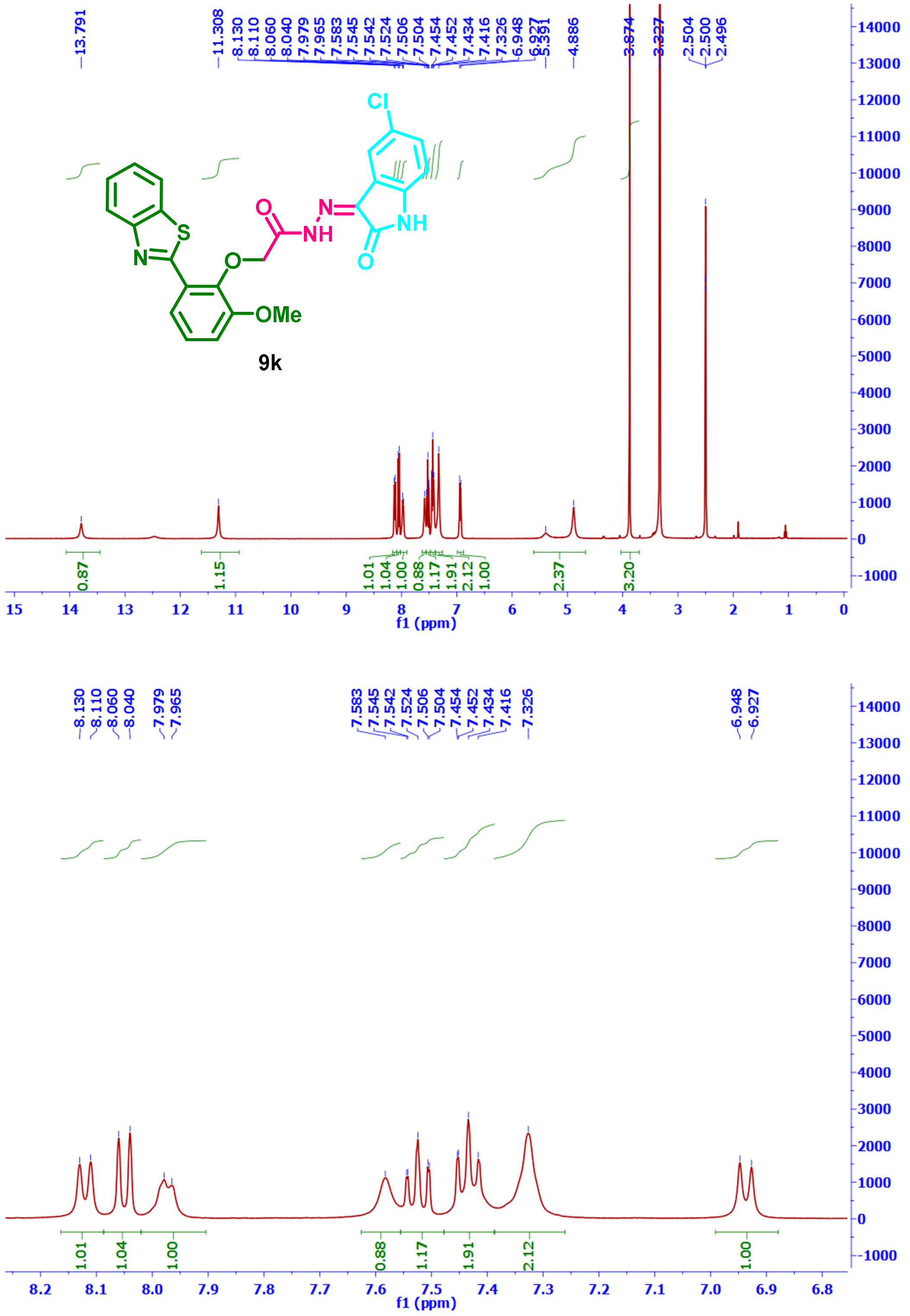

Figure 21. ^{1}H (400 MHz) NMR spectrum of **9k** in DMSO-d_6

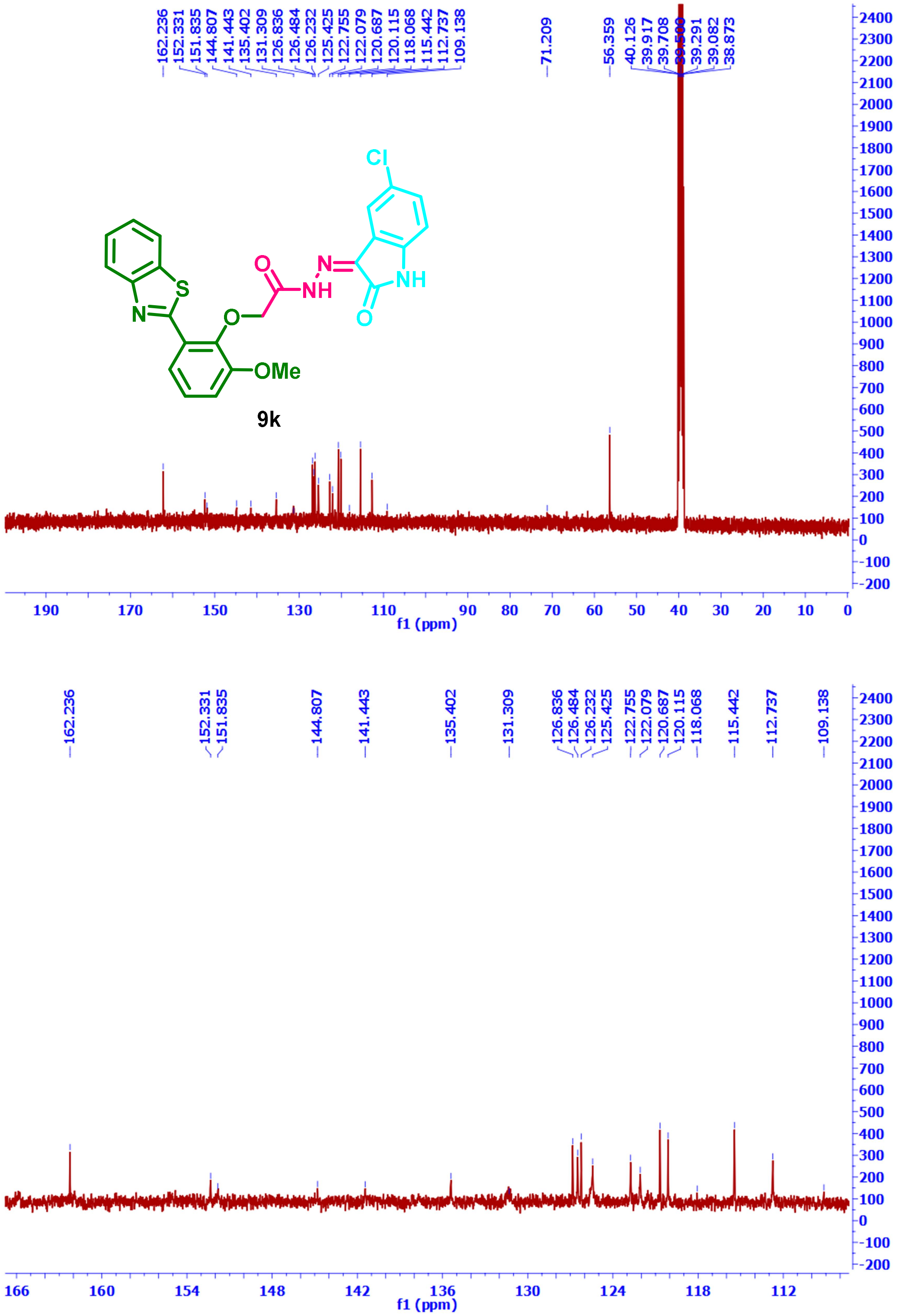

Figure 22. ^{13}C (100 MHz) NMR spectrum of **9k** in DMSO-d_6

2-(2-(Benzo[*d*]thiazol-2-yl)-6-methoxyphenoxy)-*N*'-(5-bromo-2-oxoindolin-3-ylidene)acetohydrazide (**9l**)

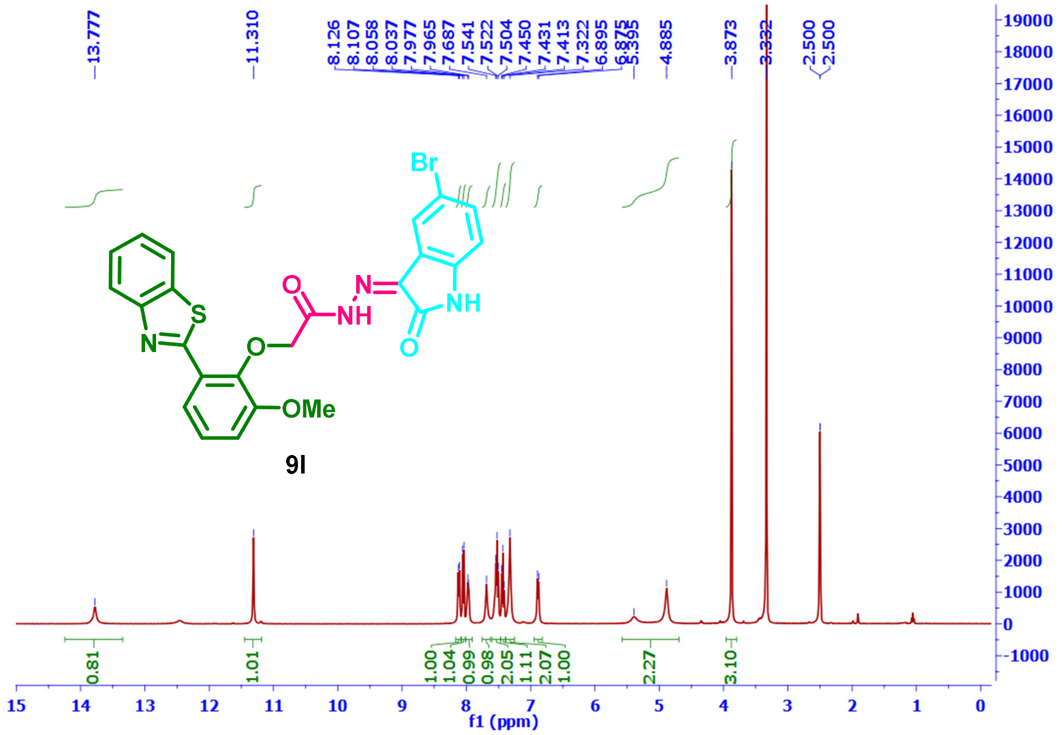

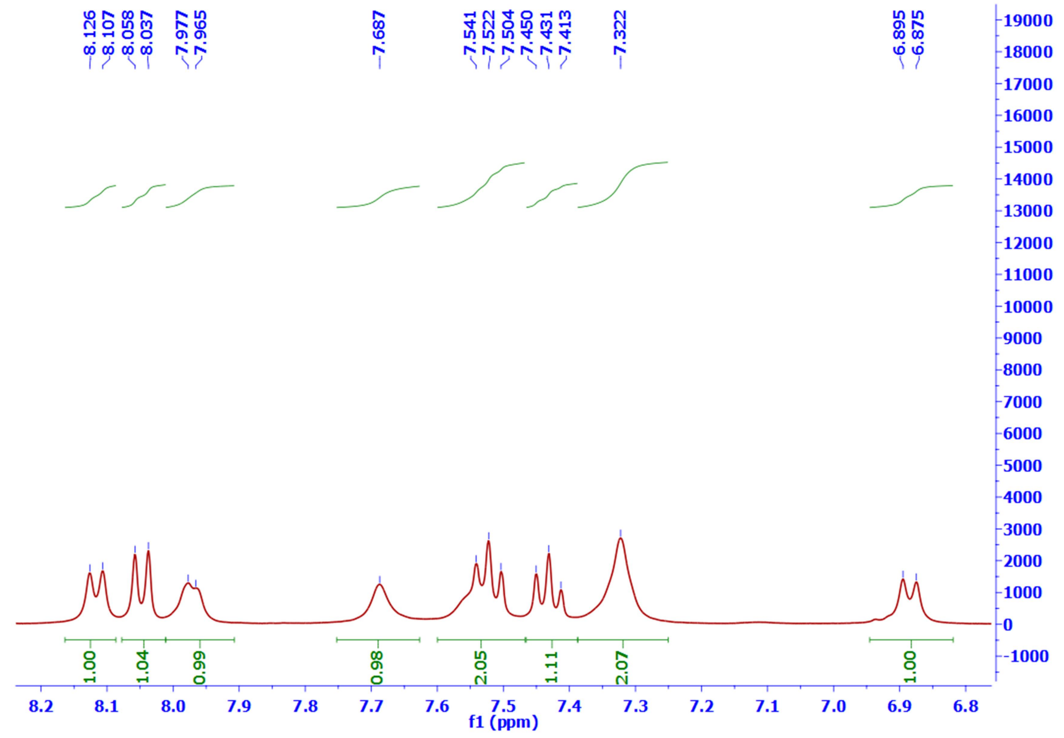

Figure 23. ^{1}H (400 MHz) NMR spectrum of **9l** in DMSO-d_6

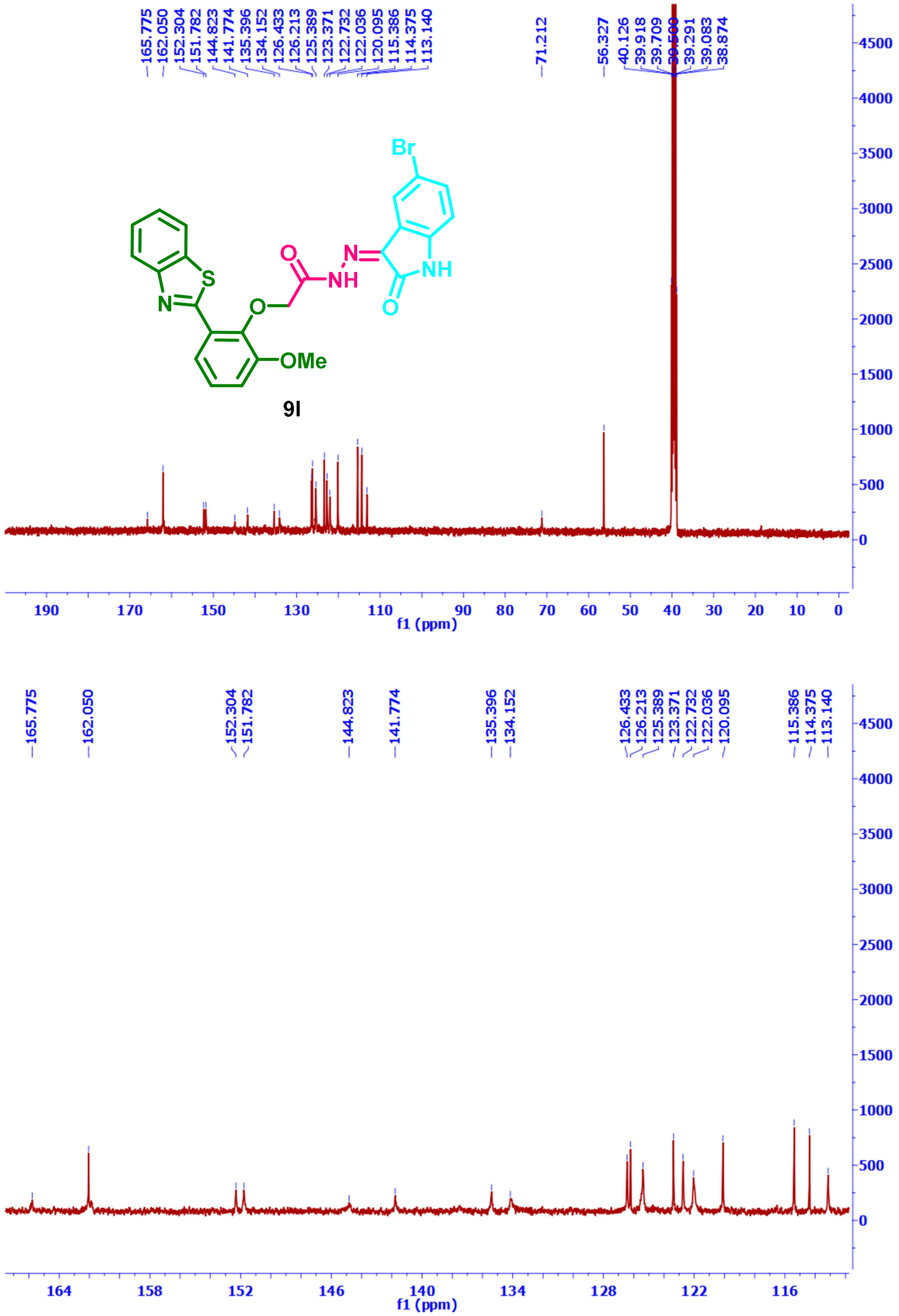

Figure 24. ^{13}C (100 MHz) NMR spectrum of **9l** in DMSO-d_6

2-(5-(Benzo[*d*]thiazol-2-yl)-2-methoxyphenoxy)-*N*'-(2-oxoindolin-3-ylidene)acetohydrazide (**9m**)

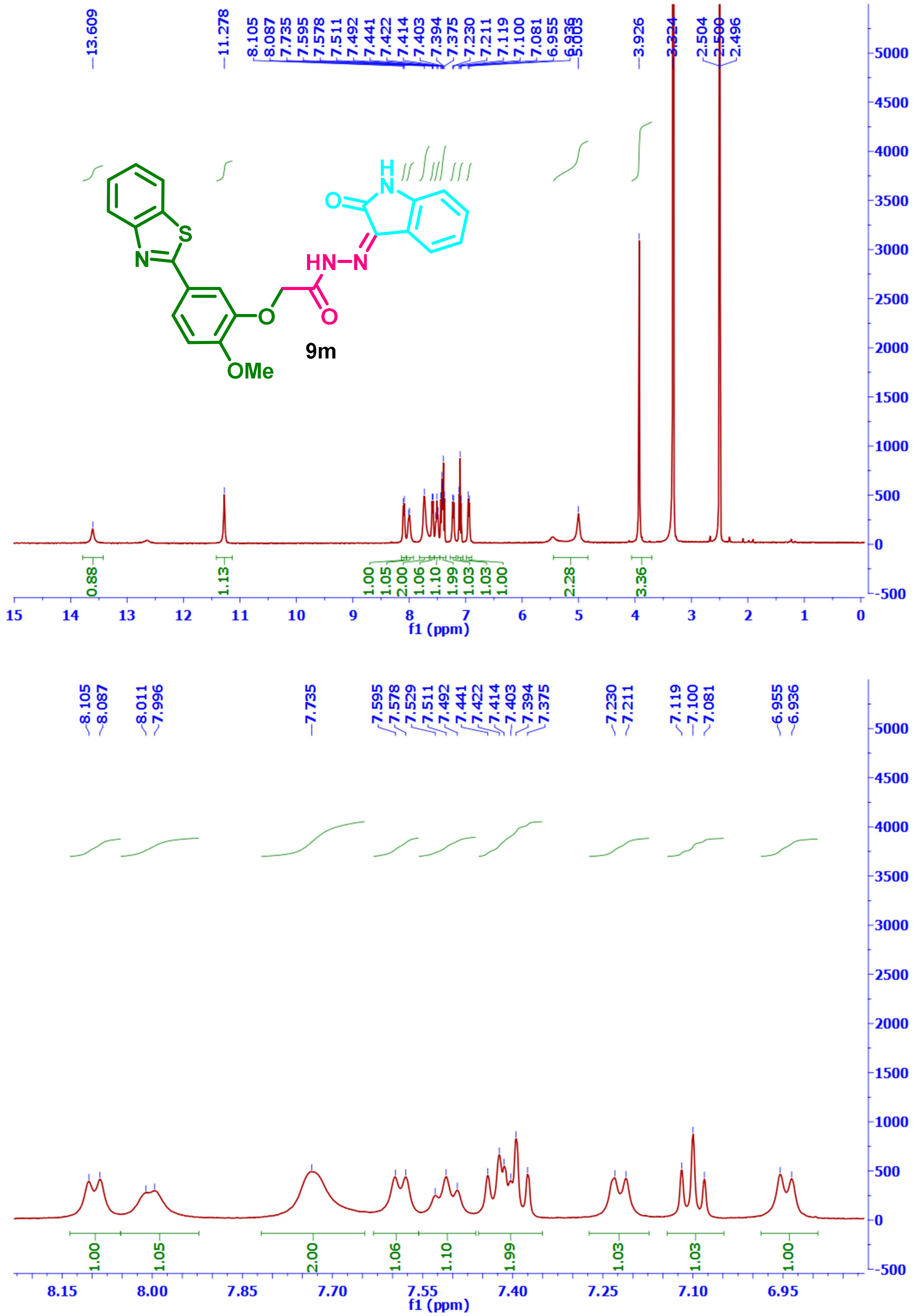

Figure 25. ^{1}H (400 MHz) NMR spectrum of **9m** in DMSO-d_6

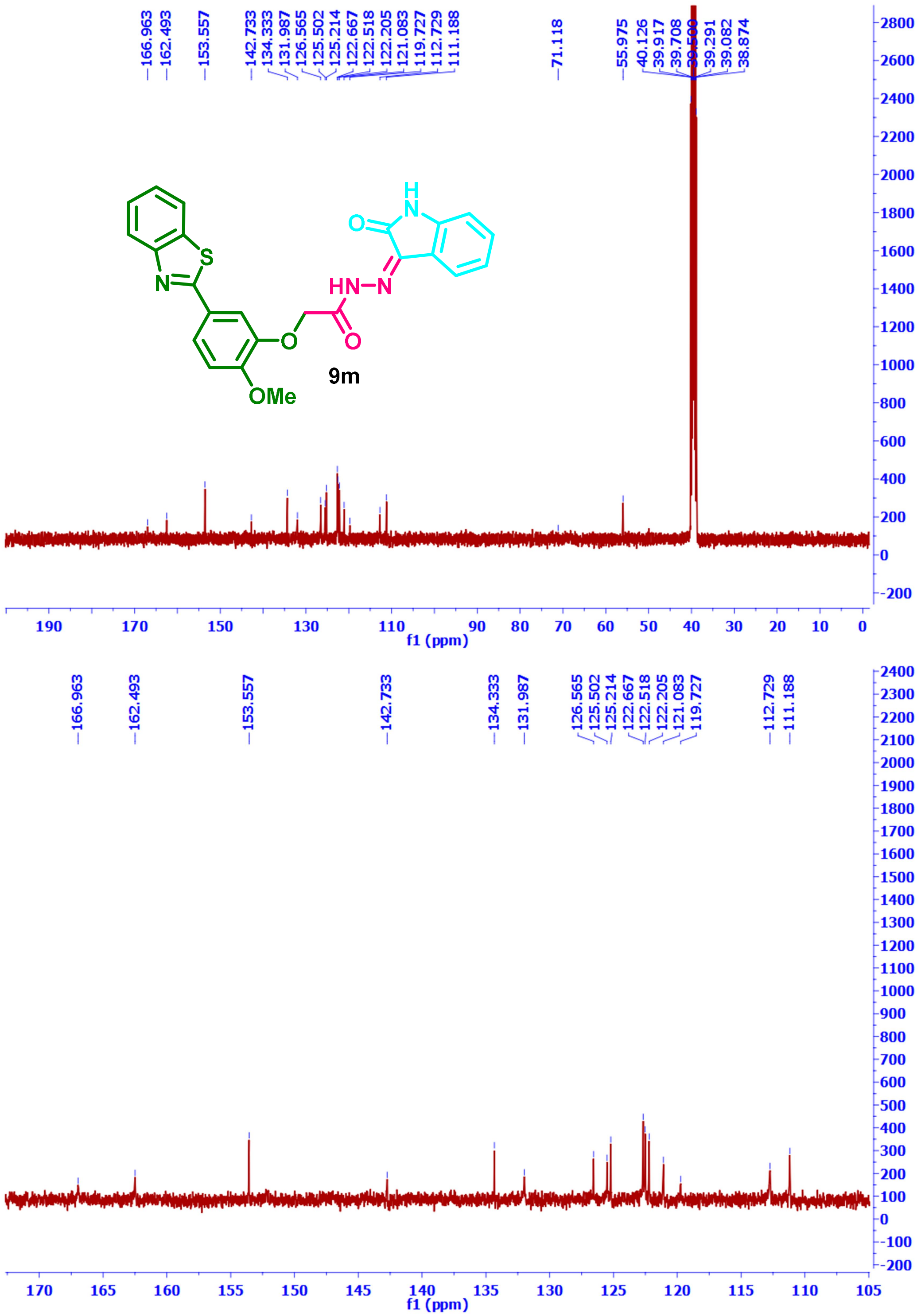

Figure 26. ^{13}C (100 MHz) NMR spectrum of **9m** in DMSO-d_6

2-(5-(Benzo[*d*]thiazol-2-yl)-2-methoxyphenoxy)-*N*'-(5-methyl-2-oxoindolin-3-ylidene)acetohydrazide (**9n**)

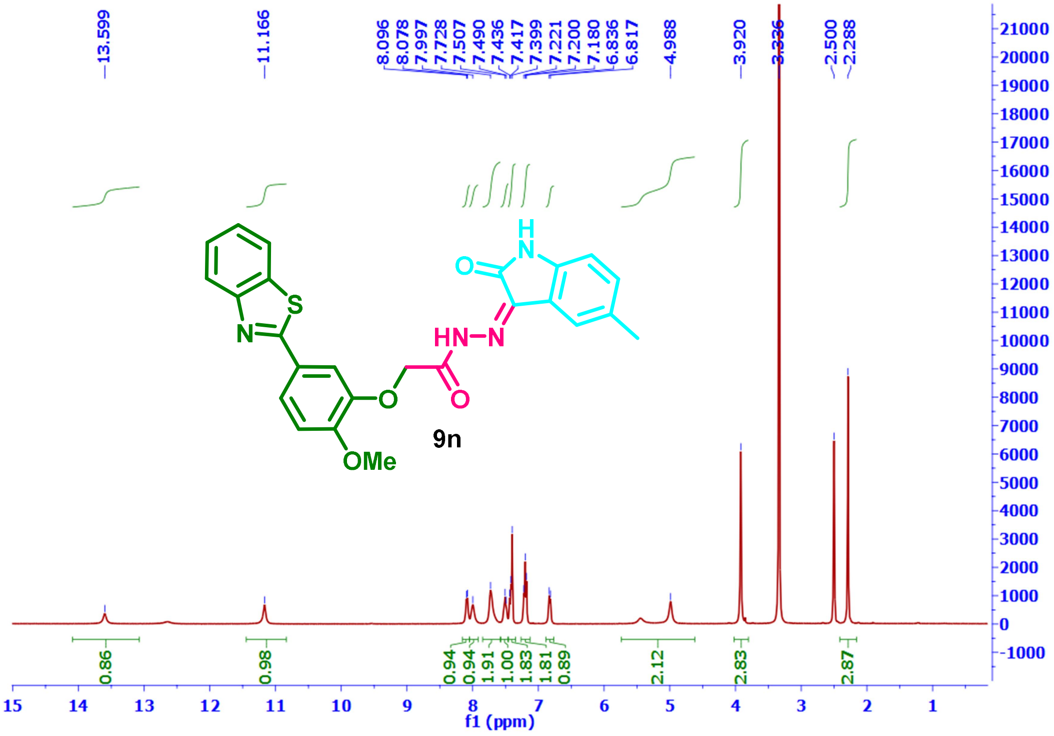

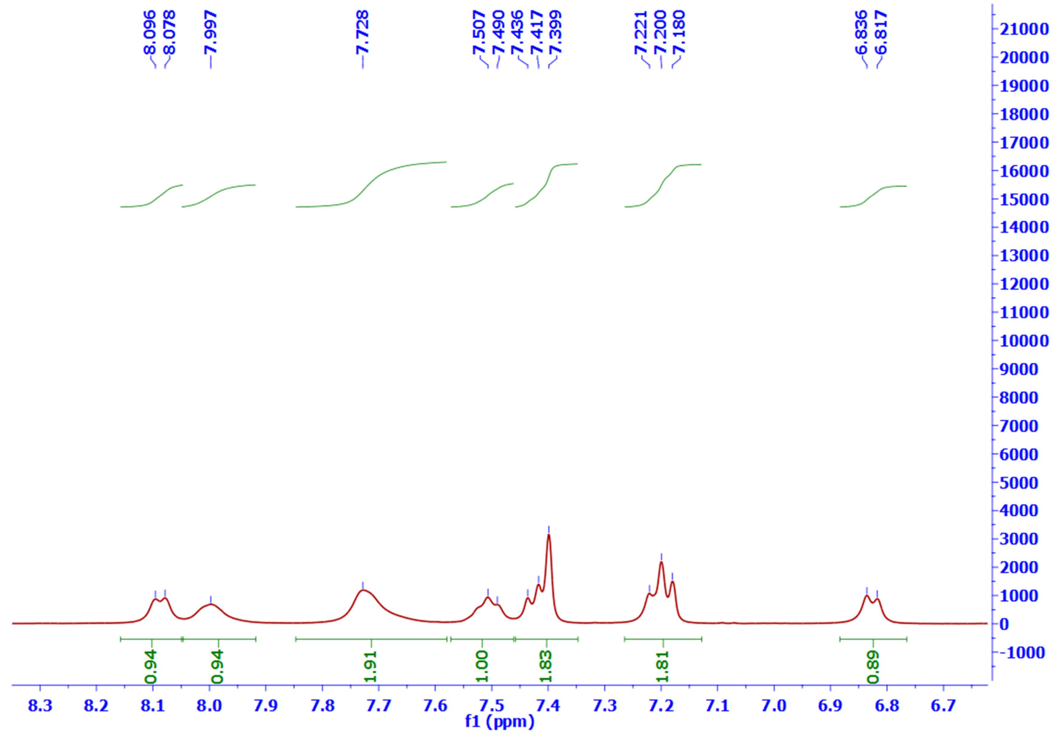

Figure 27. ^{1}H (400 MHz) NMR spectrum of **9n** in DMSO-d_6

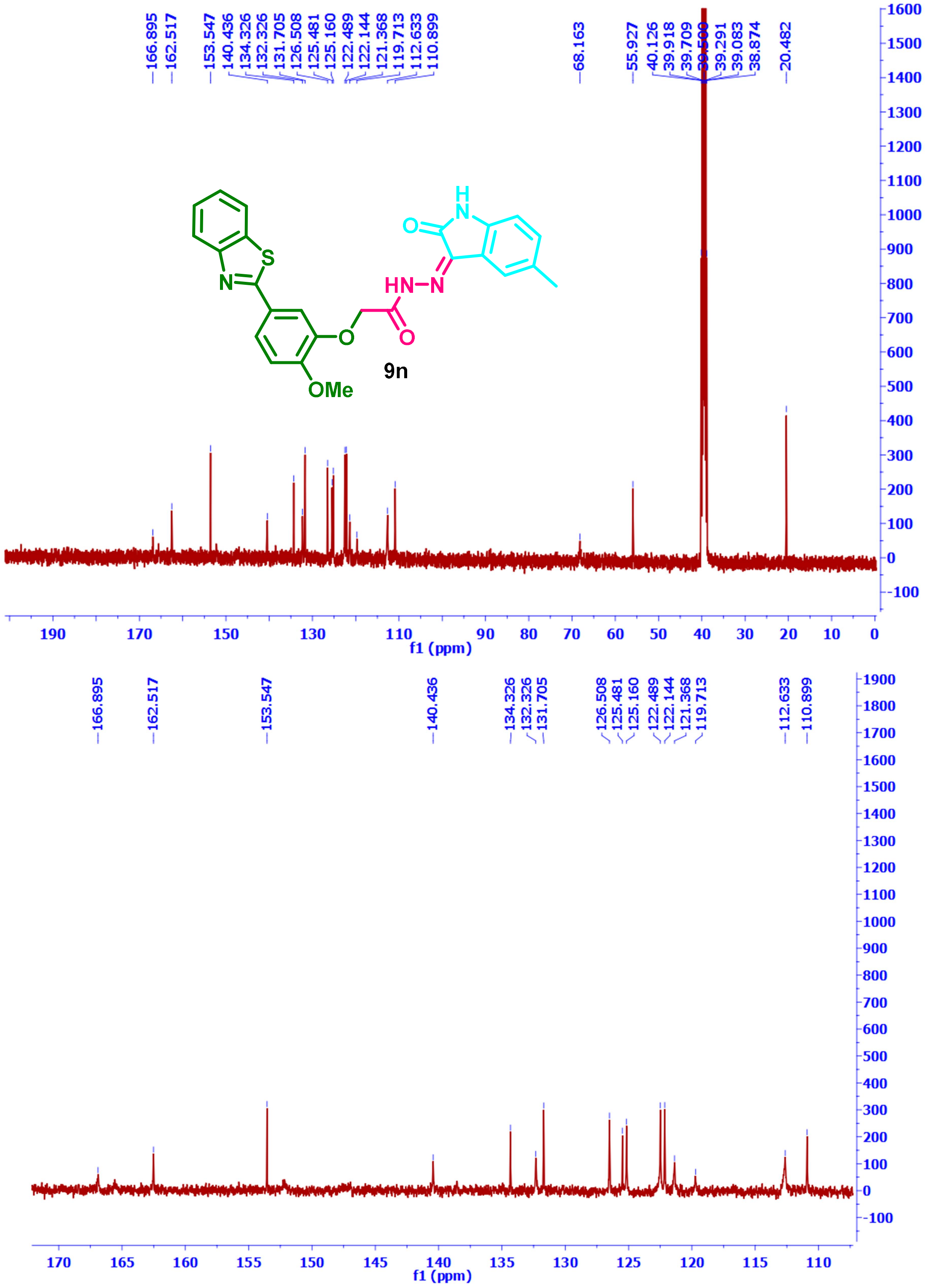

Figure 28. ^{13}C (100 MHz) NMR spectrum of **9n** in DMSO-d_6

2-(5-(Benzo[*d*]thiazol-2-yl)-2-methoxyphenoxy)-*N'*-(5-methoxy-2-oxoindolin-3-ylidene)acetohydrazide (**9o**)

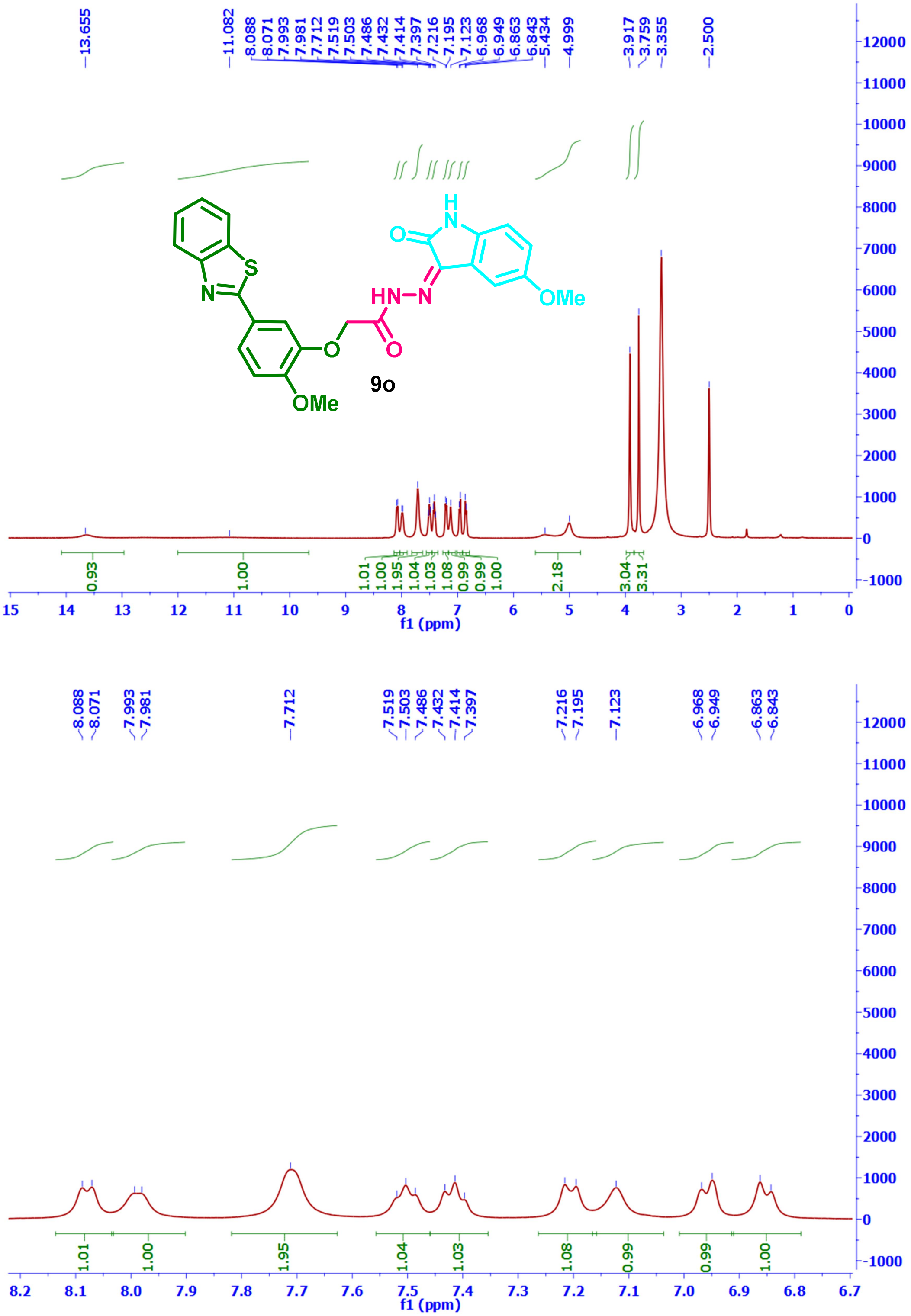

Figure 29. ^{1}H (400 MHz) NMR spectrum of **9o** in DMSO-d_6

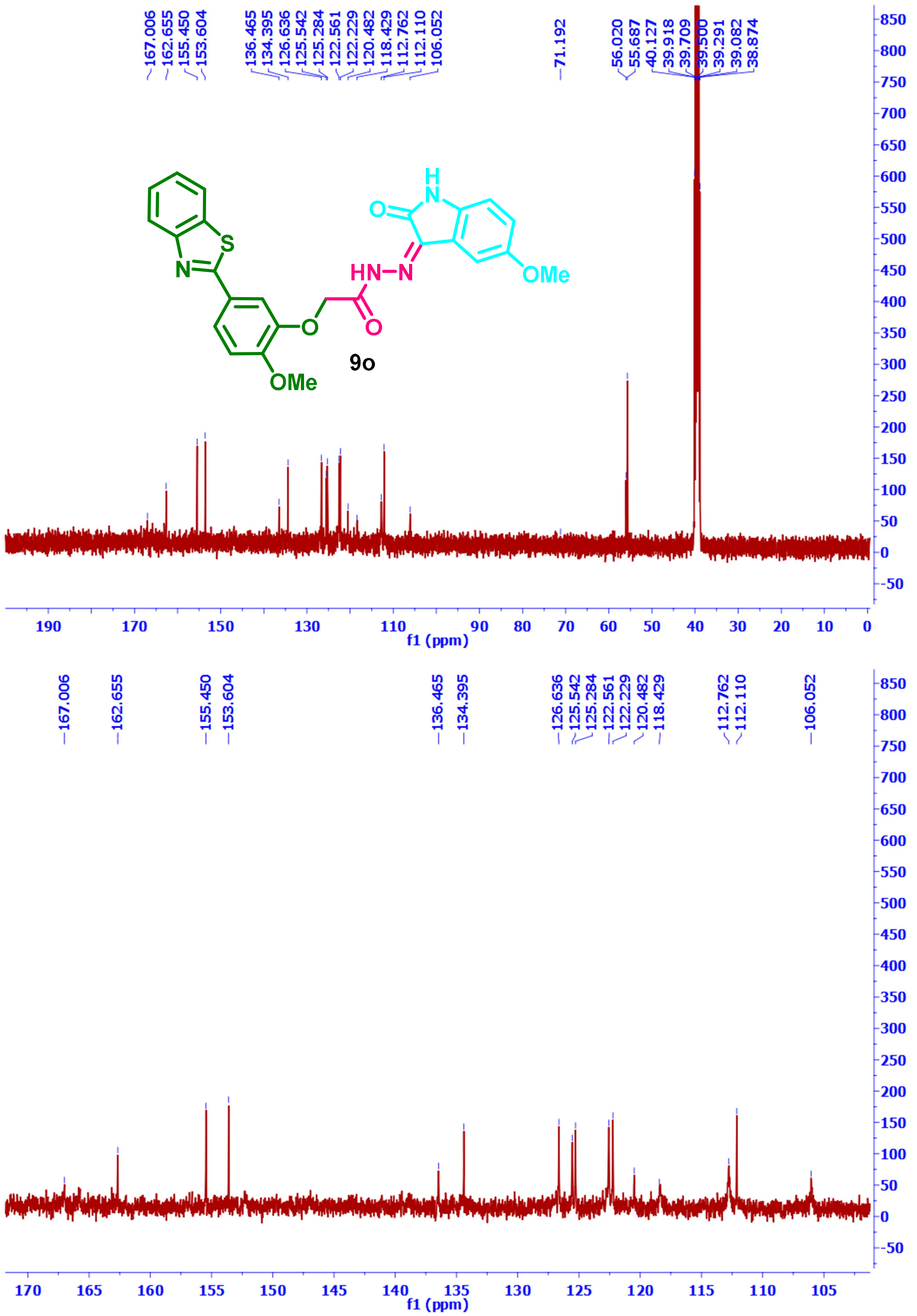

Figure 30. ^{13}C (100 MHz) NMR spectrum of **9o** in DMSO-d_6

2-(5-(Benzo[*d*]thiazol-2-yl)-2-methoxyphenoxy)-*N*'-(5-nitro-2-oxoindolin-3-ylidene)acetohydrazide (**9p**)

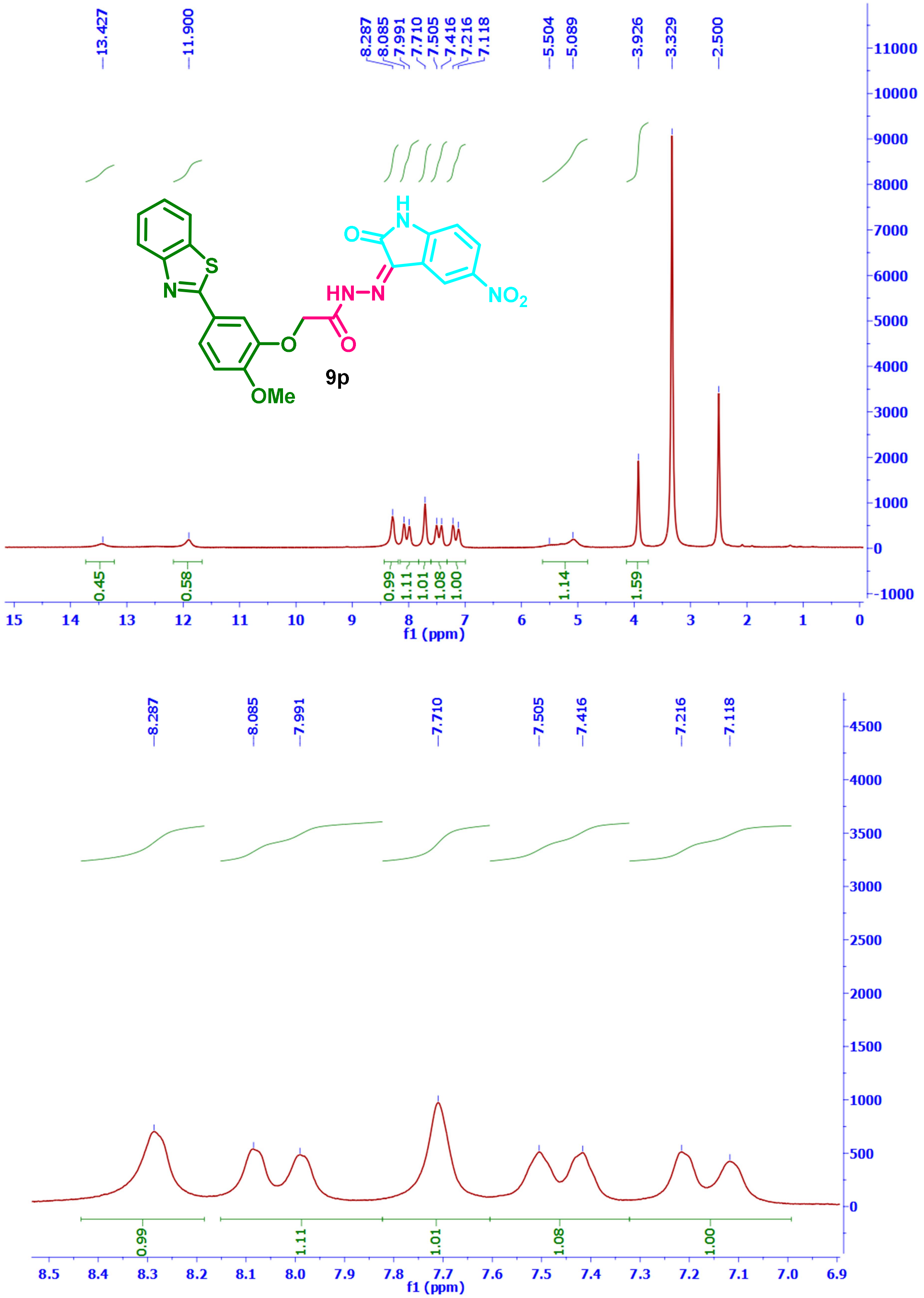

Figure 31. ^{1}H (400 MHz) NMR spectrum of **9p** in DMSO-d_6

2-(5-(Benzo[*d*]thiazol-2-yl)-2-methoxyphenoxy)-*N*'-(5-chloro-2-oxoindolin-3-ylidene)acetohydrazide (**9q**)

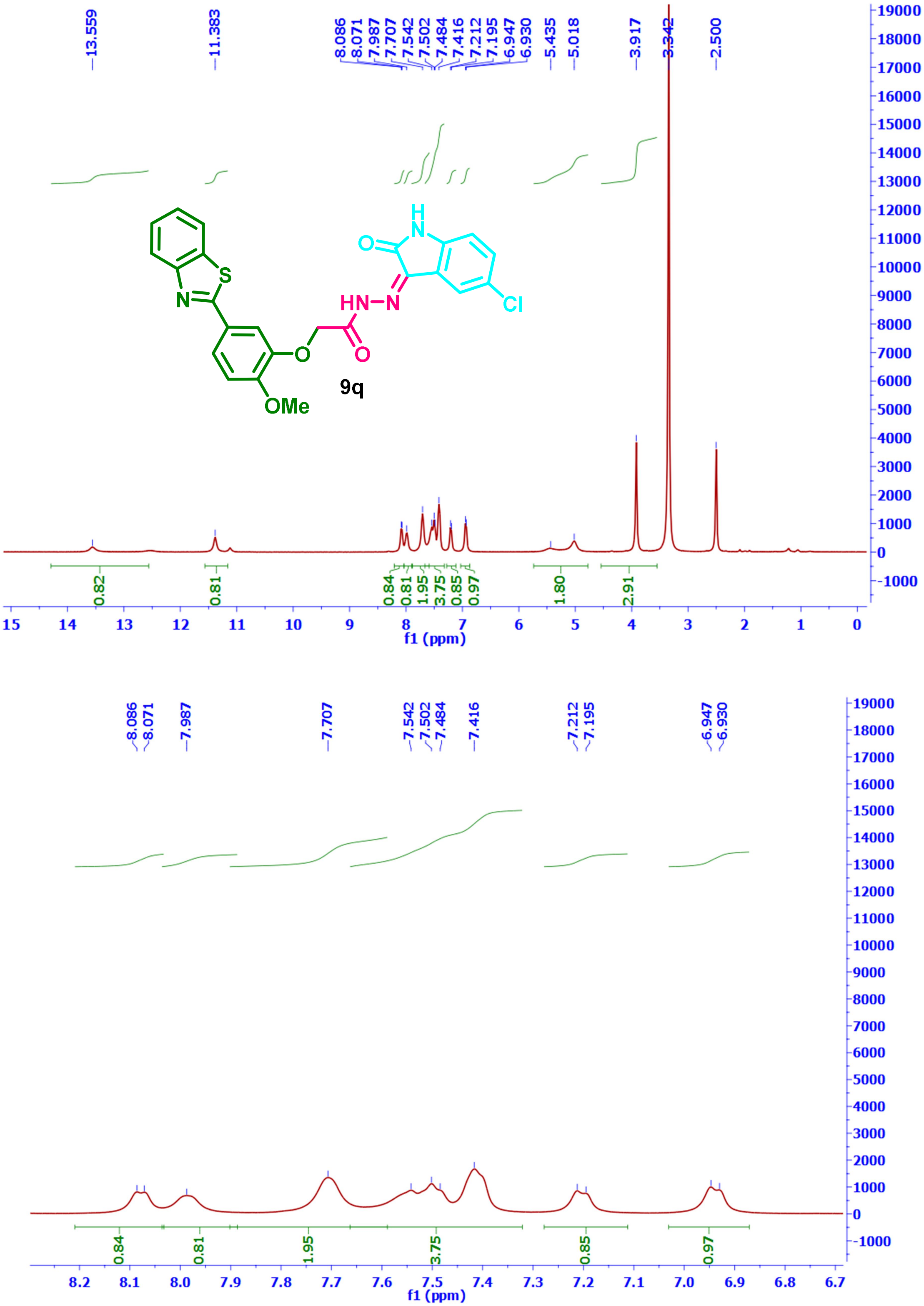

Figure 32. ^{1}H (400 MHz) NMR spectrum of **9q** in DMSO-d_6

2-(5-(Benzo[*d*]thiazol-2-yl)-2-methoxyphenoxy)-*N'*-(5-bromo-2-oxoindolin-3-ylidene)acetohydrazide (**9r**)

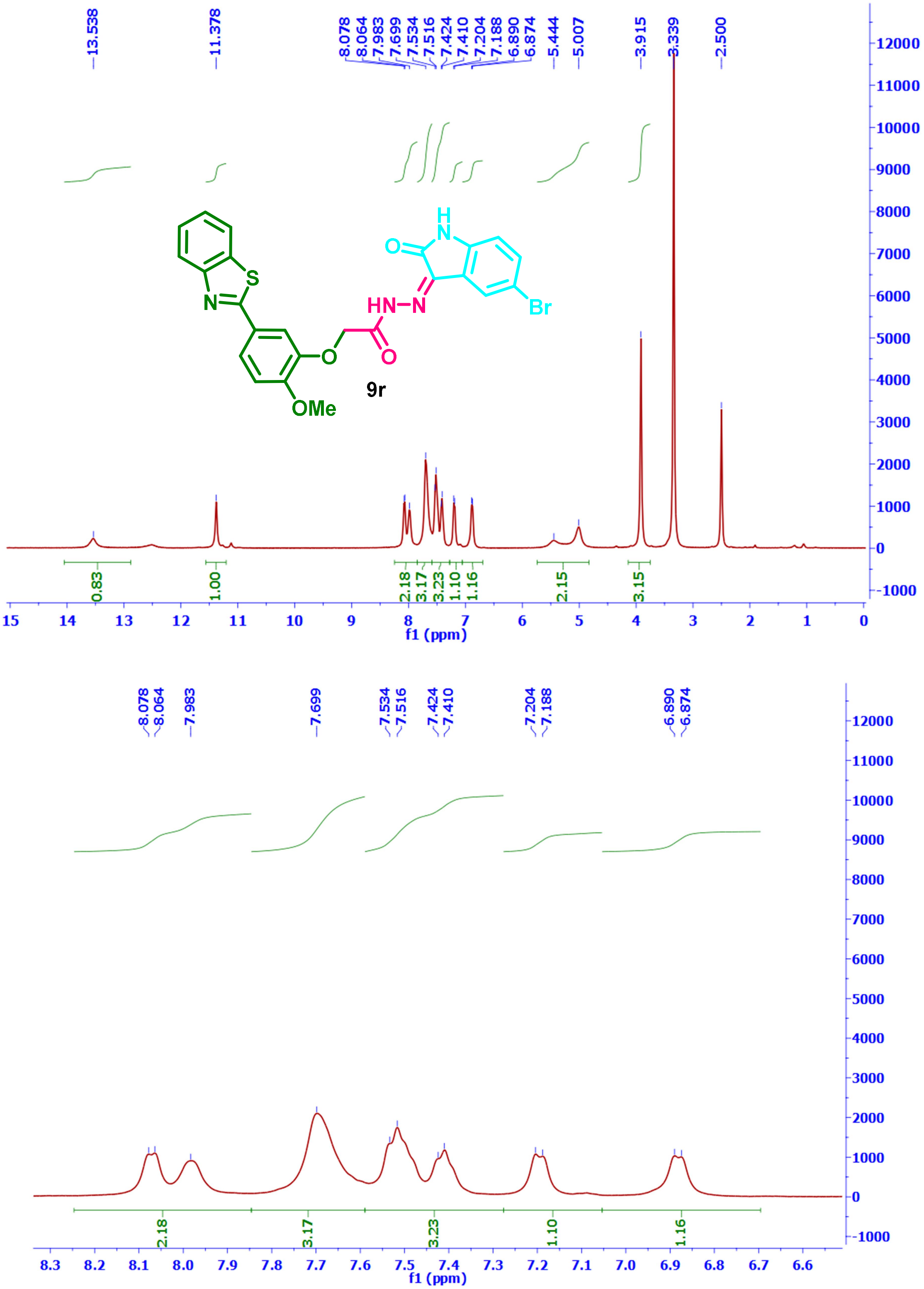

Figure 33. ^{1}H (400 MHz) NMR spectrum of **9r** in DMSO-d_6

2. IR charts of the synthesized oxindole-benzothiazoles

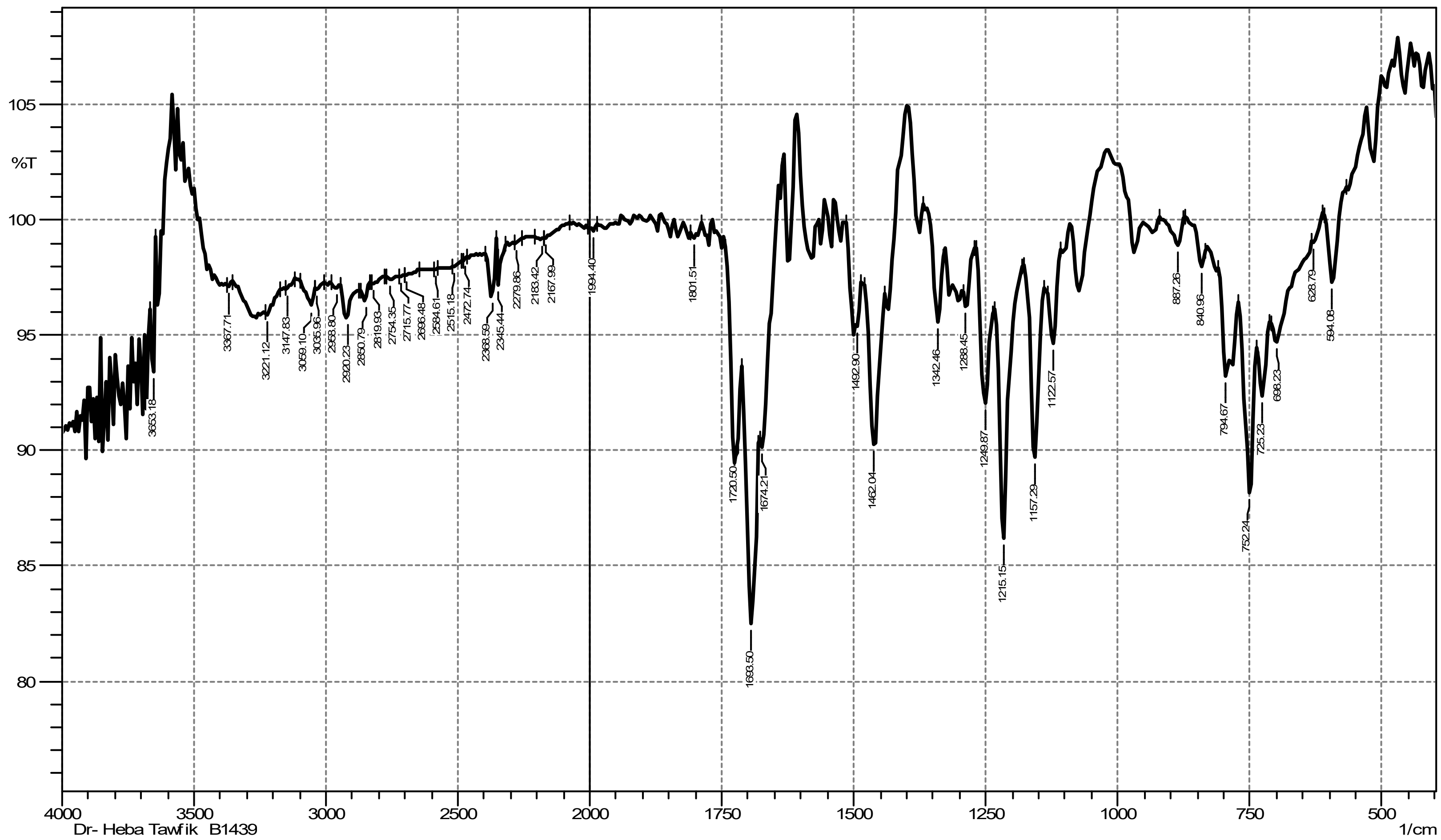

Figure 34. IR chart of **9a**

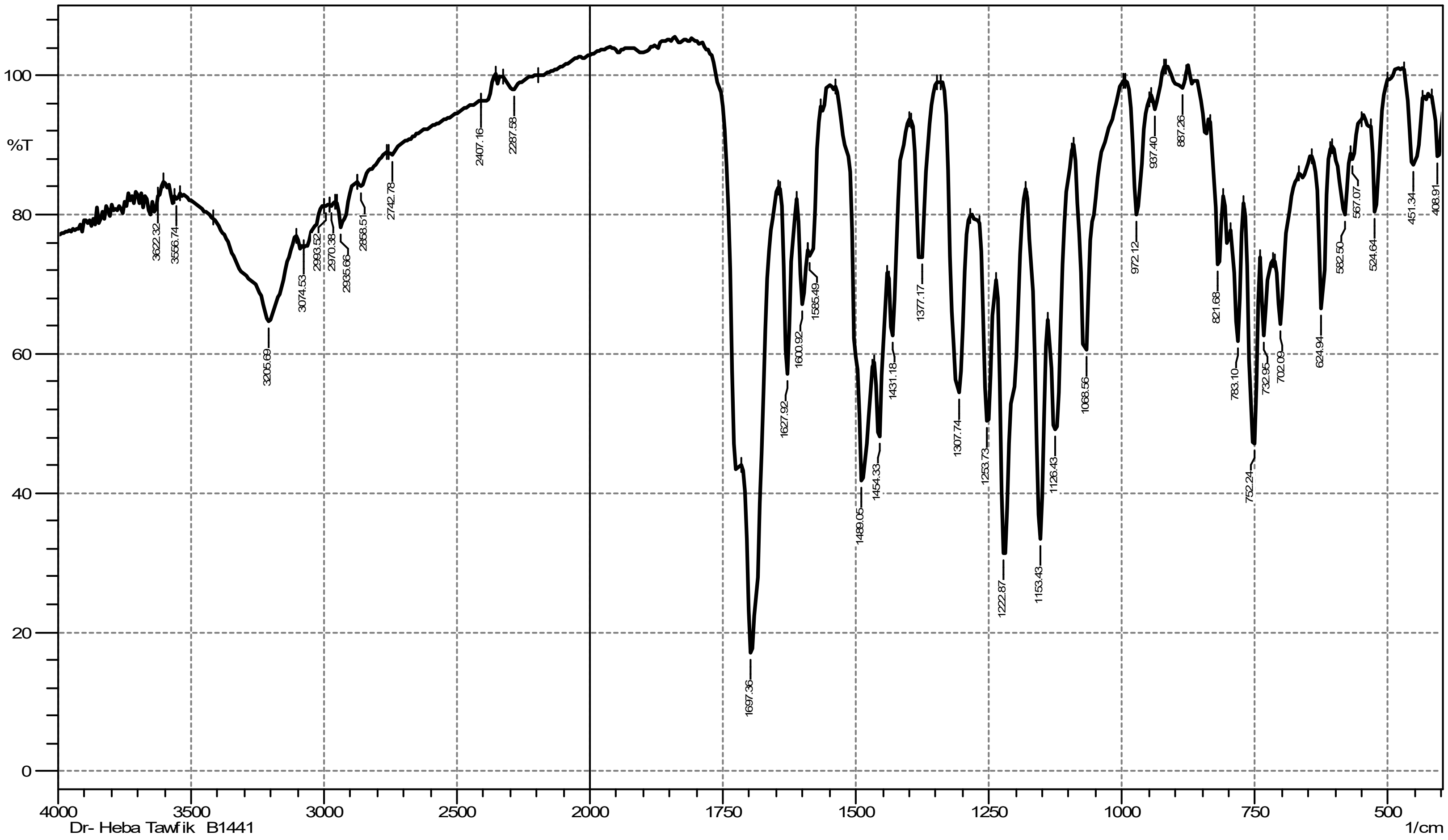

Figure 35. IR chart of **9b**

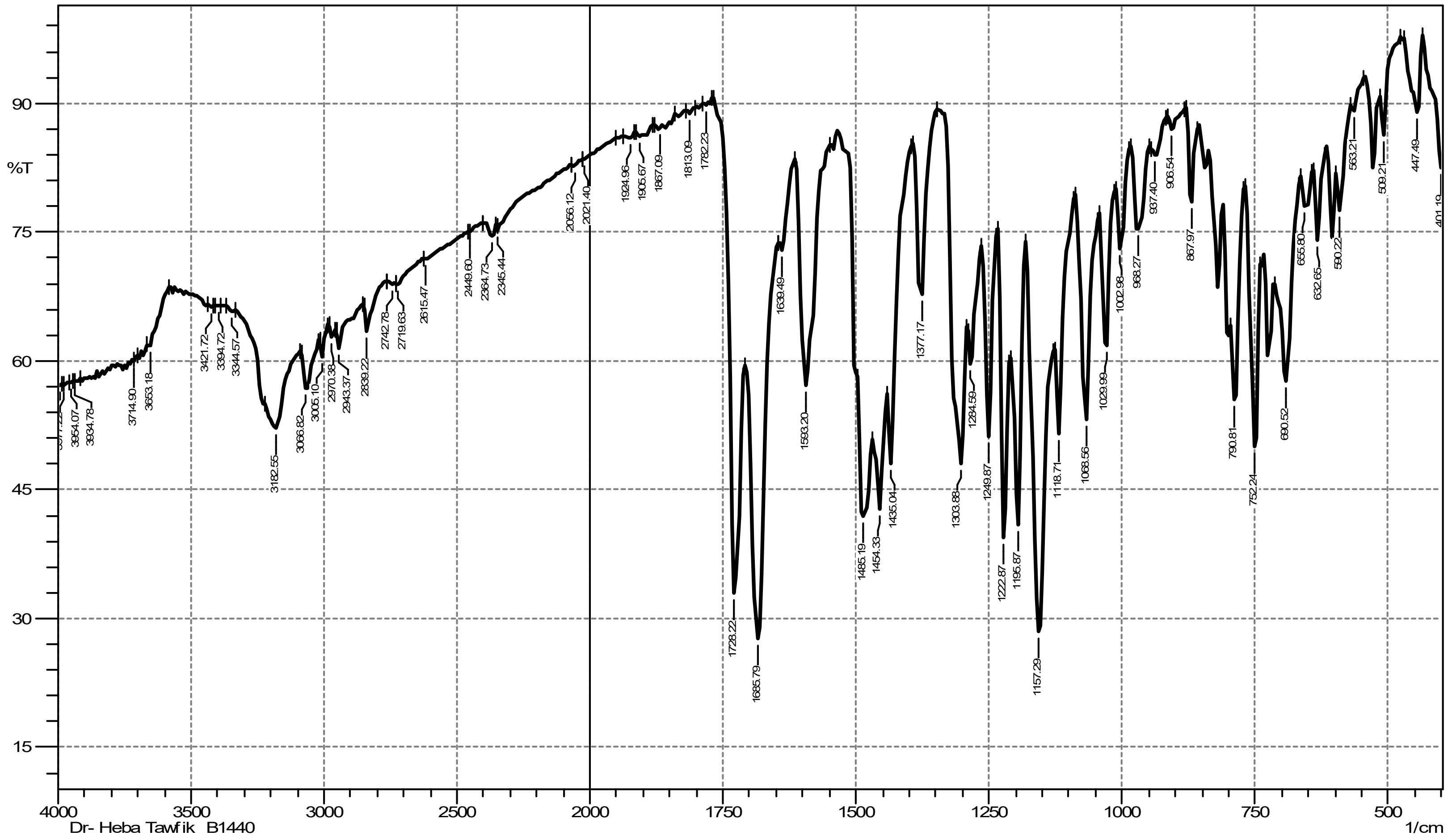

Figure 36. IR chart of **9c**

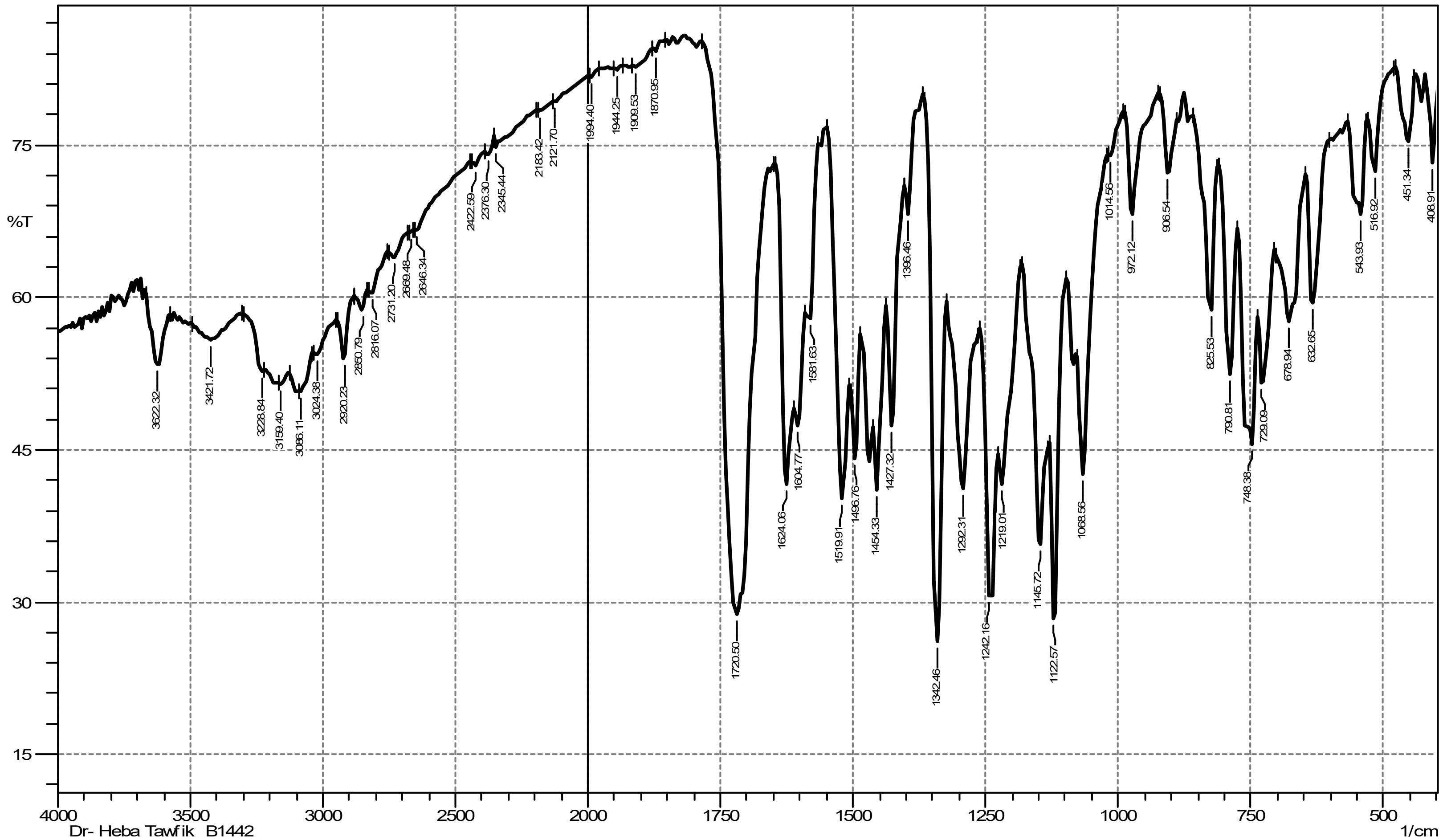

Figure 37. IR chart of **9d**

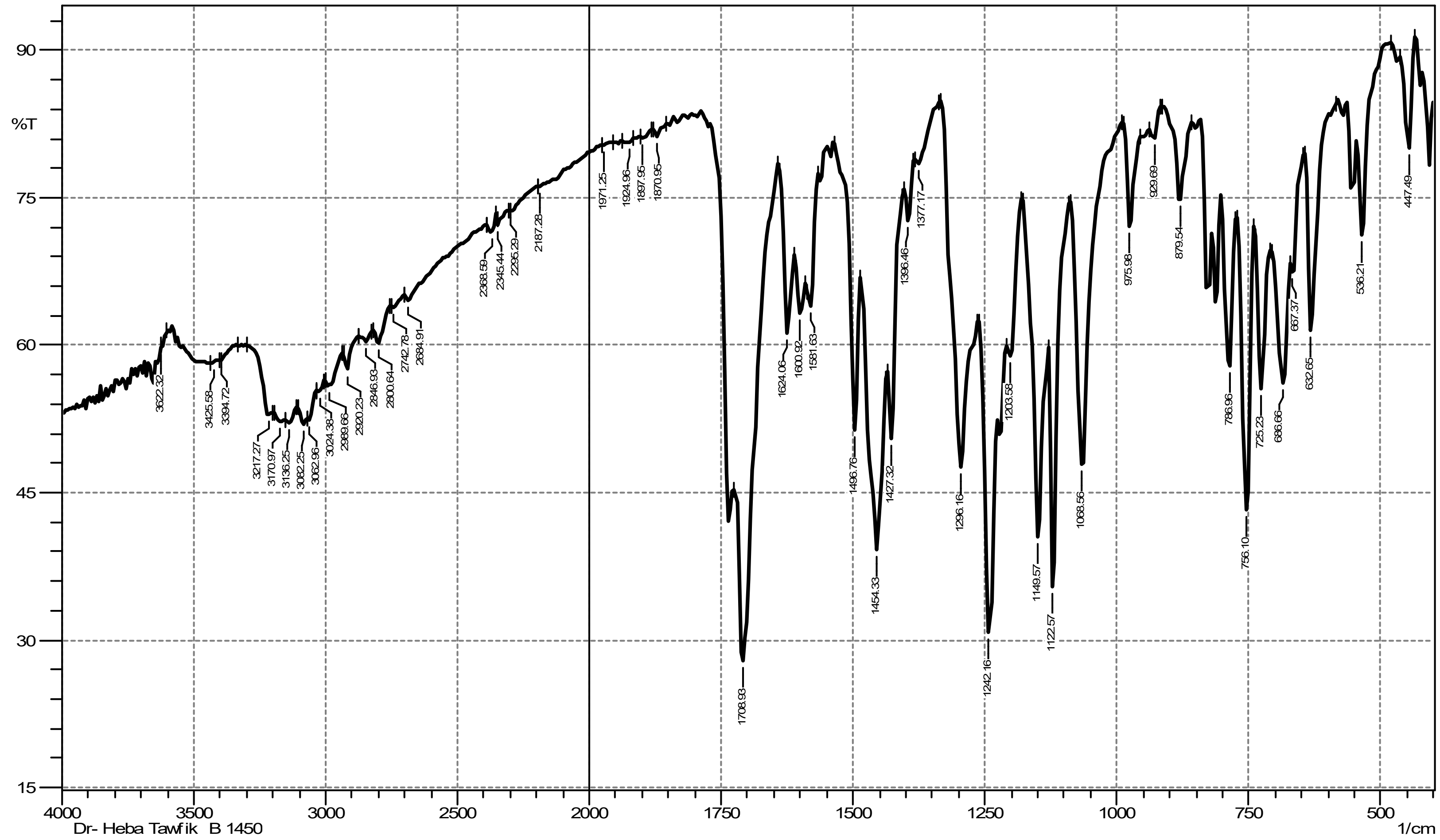

Figure 38. IR chart of **9e**

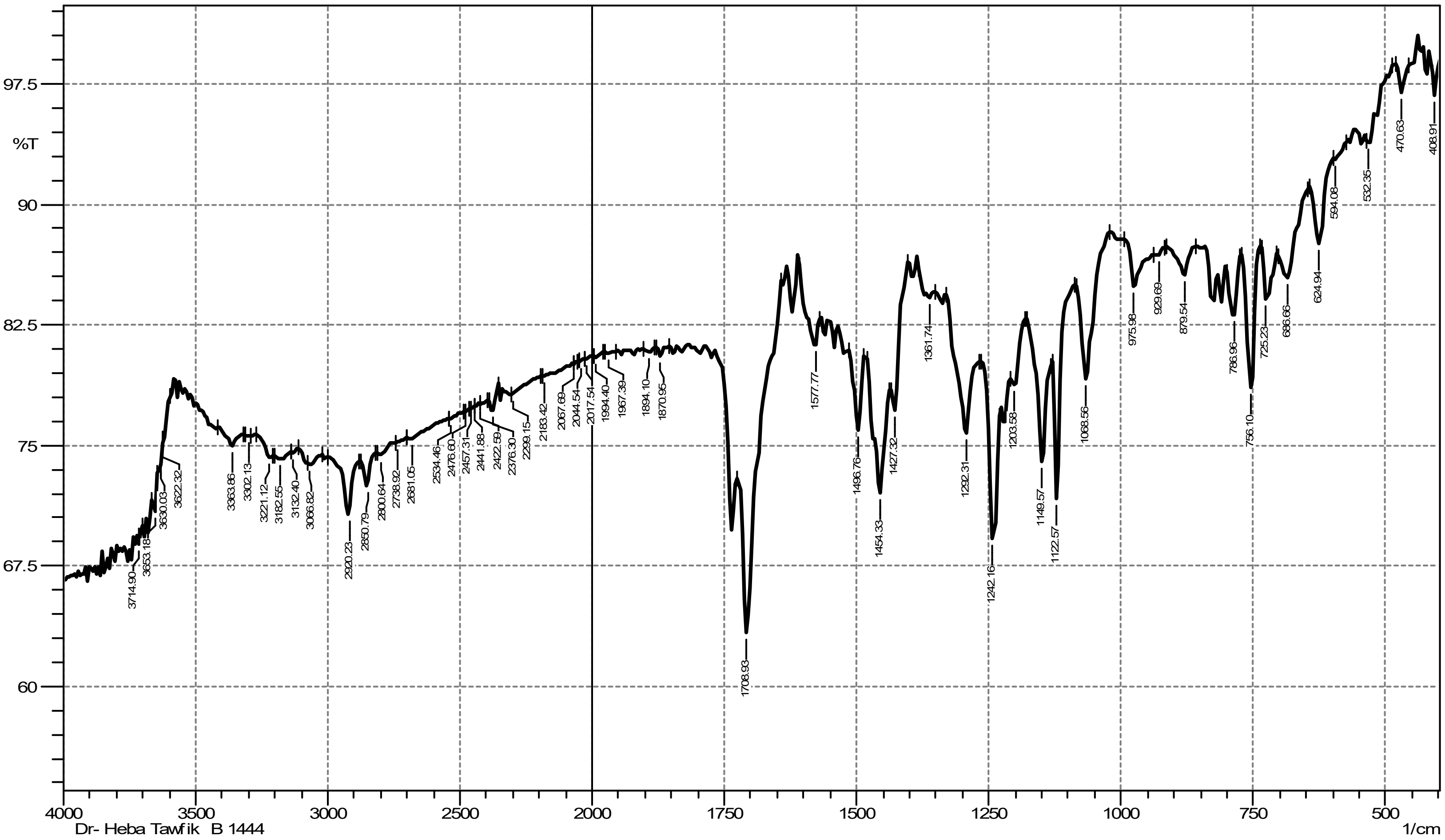

Figure 39. IR chart of **9f**

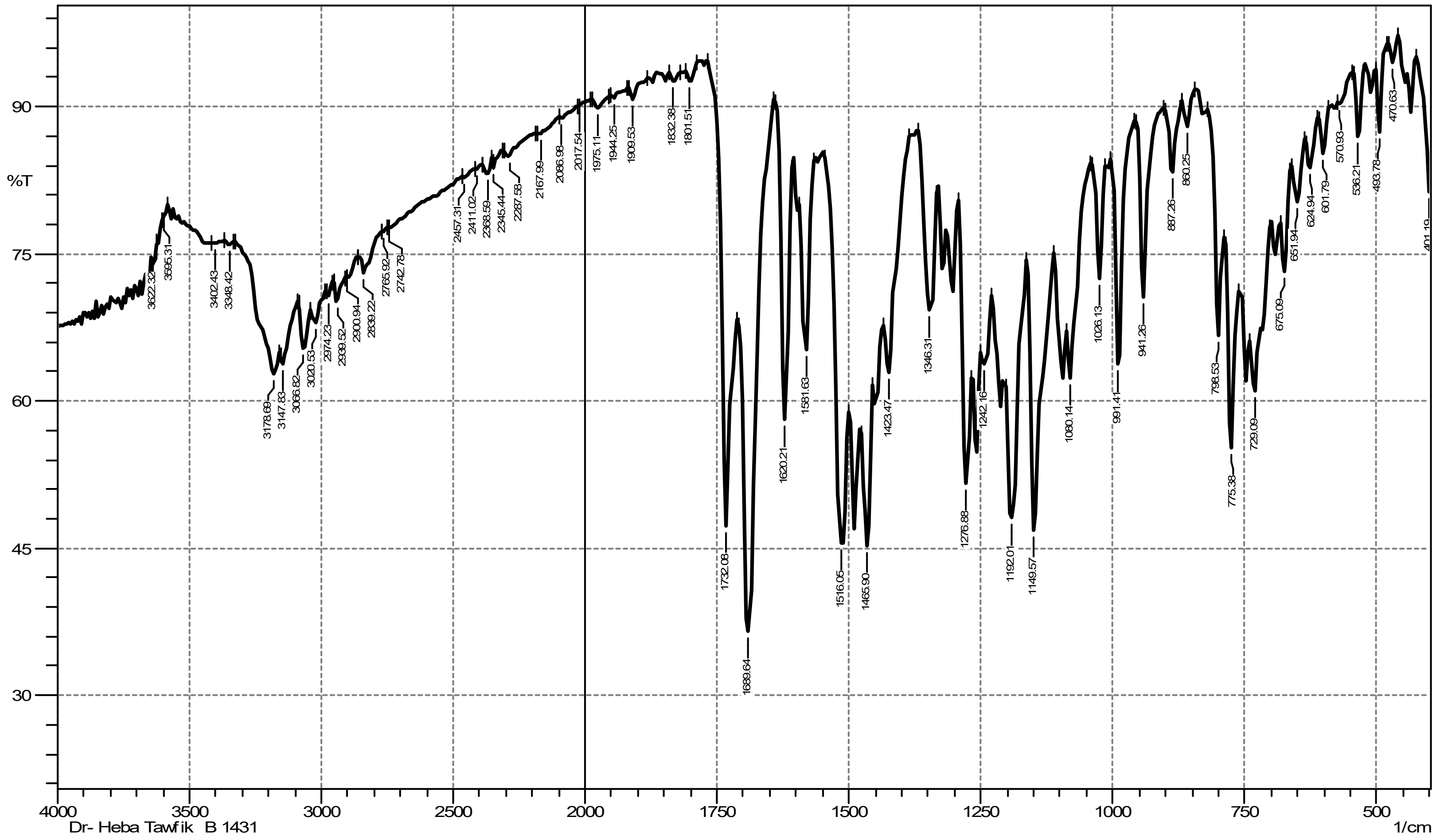

Figure 40. IR chart of **9g**

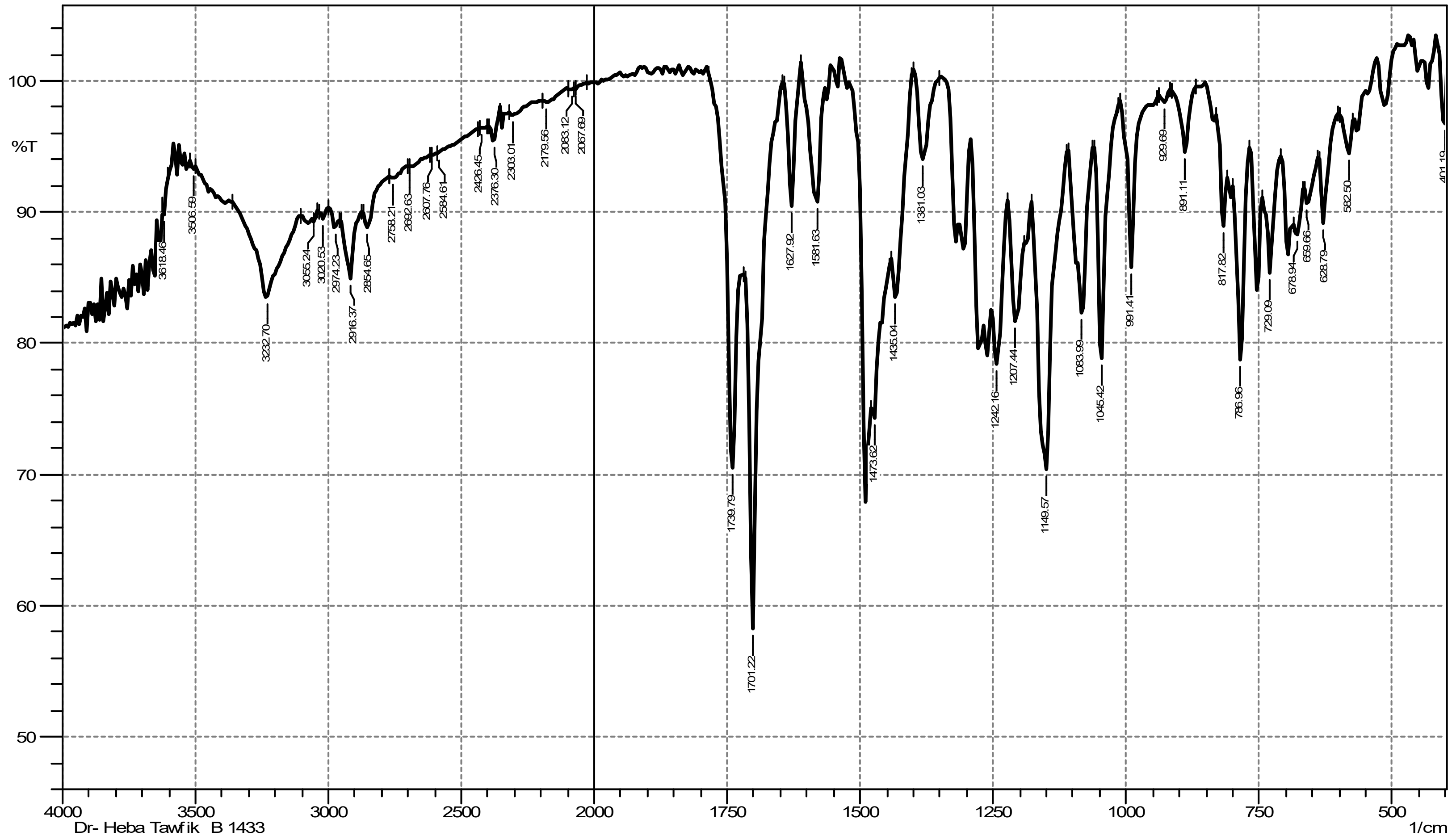

Figure 41. IR chart of **9h**

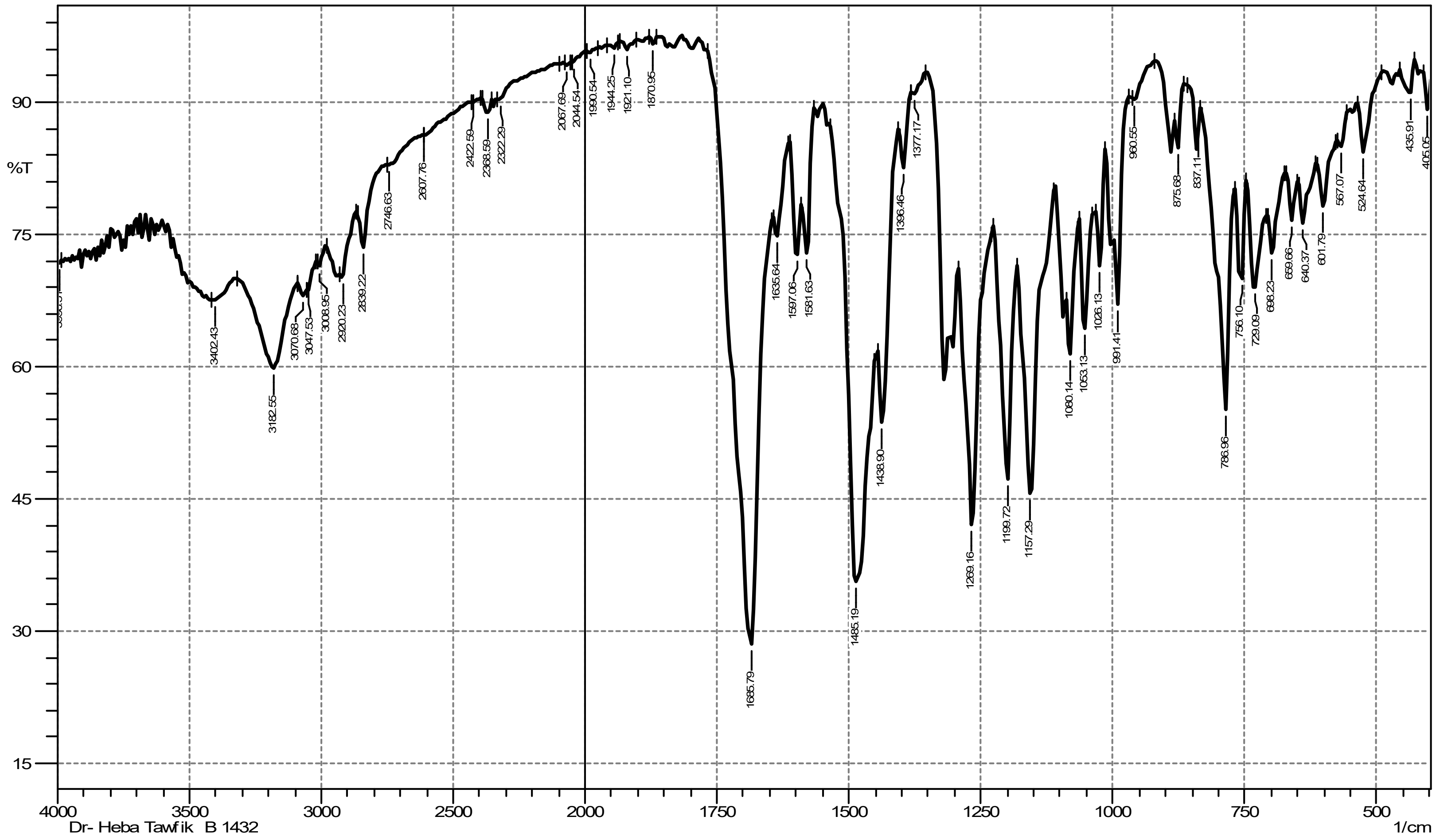

Figure 42. IR chart of **9i**

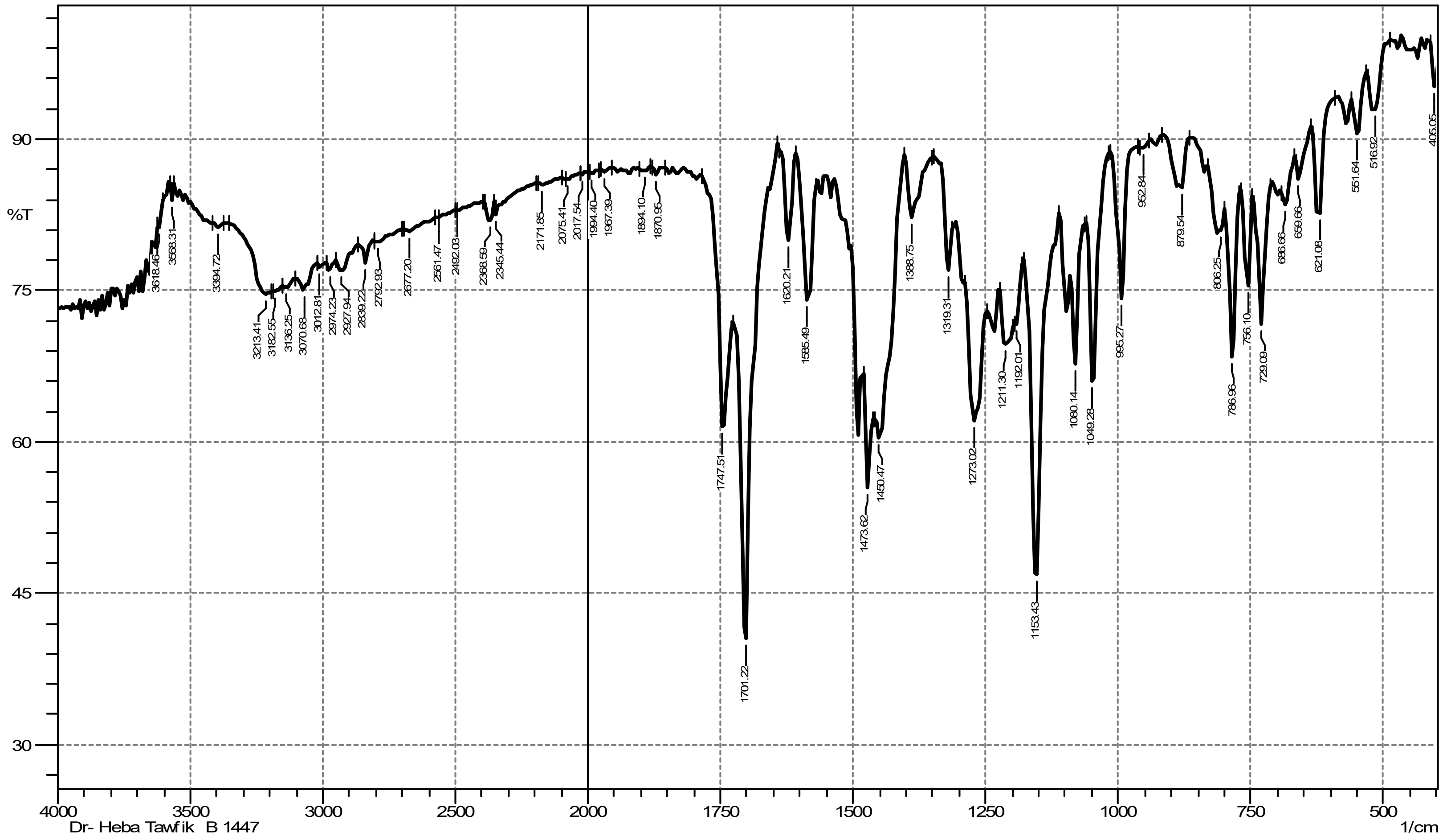

Figure 43. IR chart of **9k**

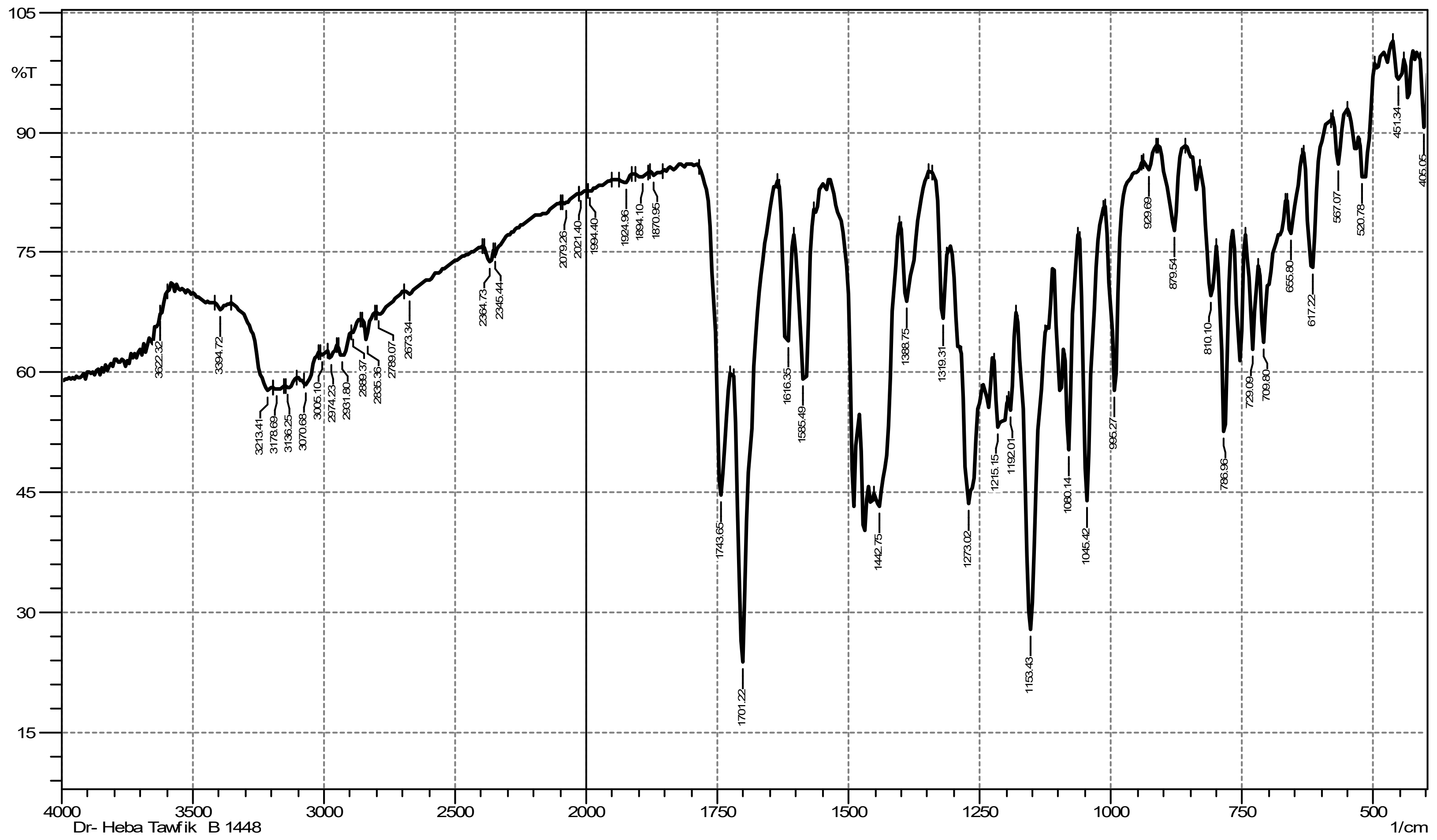

Figure 44. IR chart of **9l**

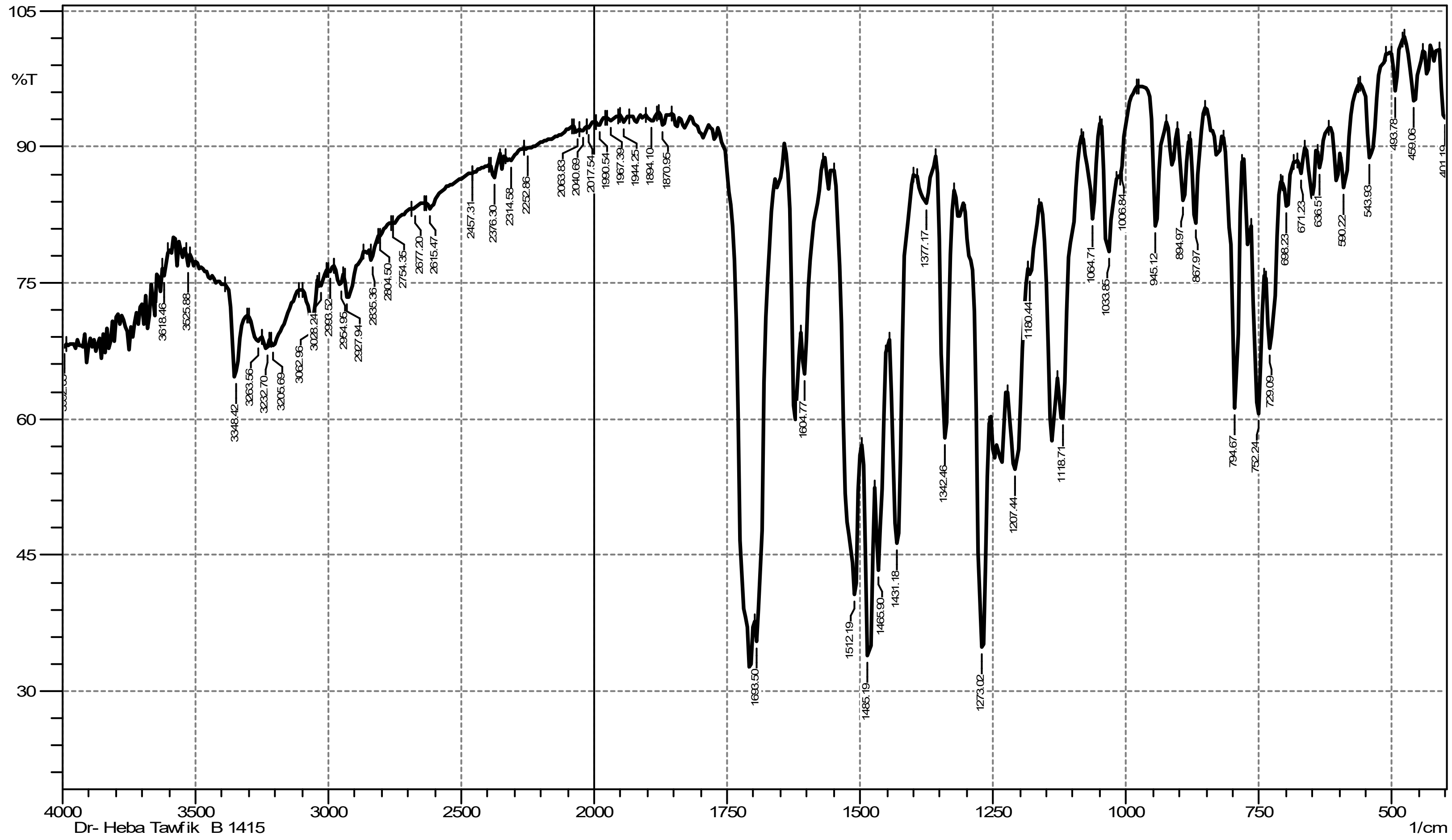

Figure 45. IR chart of **9m**

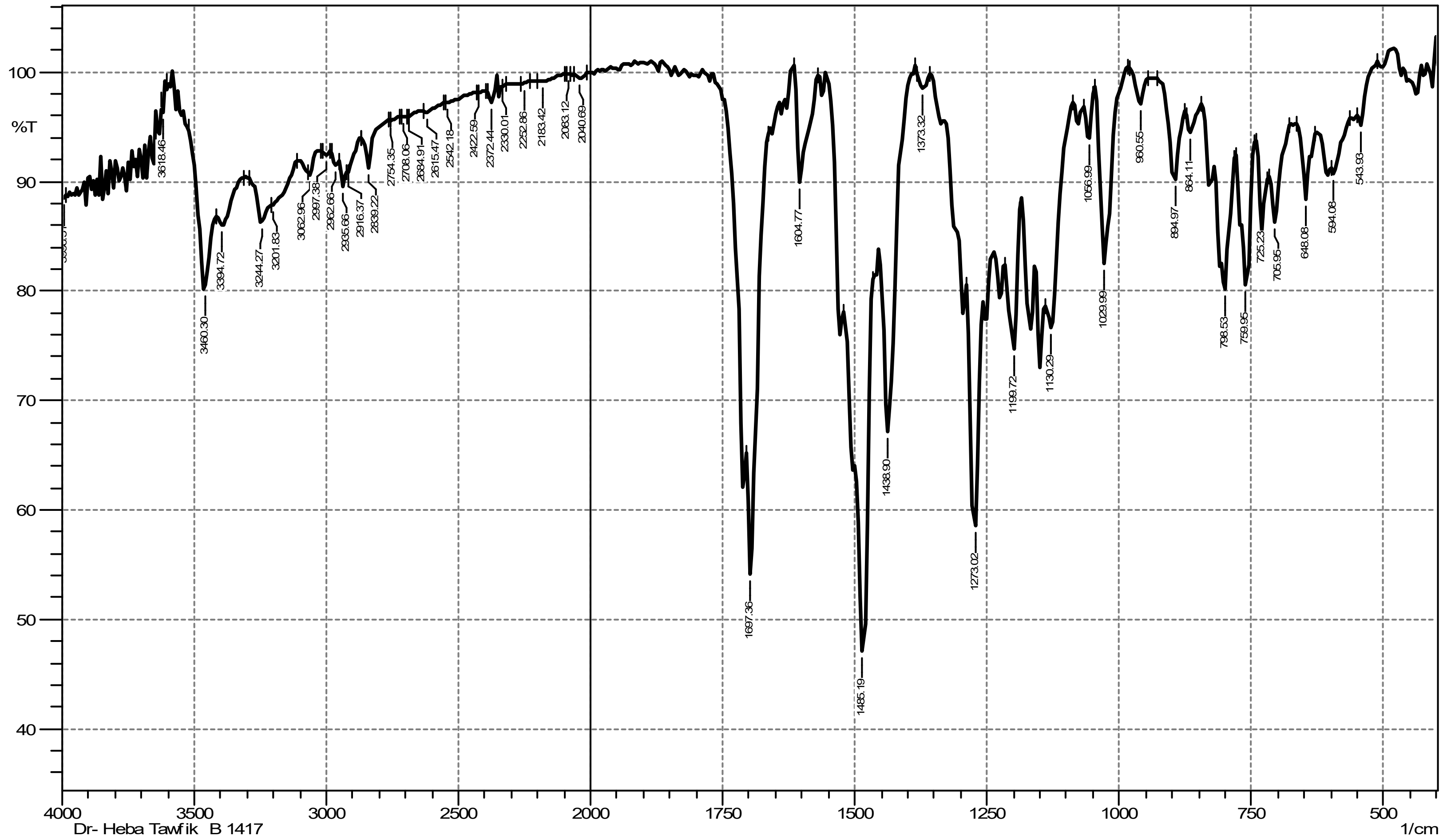

Figure 46. IR chart of **9o**

3. Screening of cytotoxic activity against a panel of sixty human tumor cell lines

The selected compounds by NCI were evaluated for their anticancer activity in a two-stage process. First, these compounds were screened against the full NCI 60 cell lines panel at a single high dose of 10 µM. Then, the output from the single dose screen is reported as a mean graph. Second, compounds exhibiting significant growth inhibition were evaluated against the 60 cell panel at five different minimal concentrations.

Assay protocol. The human tumor cell lines of the cancer screening panel are grown in RPMI 1640 medium containing 5% fetal bovine serum and 2 mM L-glutamine. For a typical screening experiment, cells are inoculated into 96 well microtiter plates in 100 µL at plating densities ranging from 5000 to 40,000 cells/well depending on the doubling time of individual cell lines. After cell inoculation, the microtiter plates are incubated at 37 ^{o}C, 5% CO_2, 95% air and 100% relative humidity for 24 h prior to addition of the experimental drugs.

After 24 h, two plates of each cell line are fixed in situ with TCA, to represent a measurement of the cell population for each cell line at the time of drug addition (Tz). The experimental drugs are solubilized in dimethyl sulfoxide at 400-fold the desired final maximum test concentration and stored frozen prior to use. At the time of drug addition, an aliquot of frozen concentrate is thawed and diluted to twice the desired final maximum test concentration with complete medium containing 50 µg/mL gentamicin. Additional four, 10-fold or ½ log serial dilutions are made to provide a total of five drug concentrations plus control.

Aliquots of 100 mL of these different drug dilutions are added to the appropriate microtiter wells already containing 100 mL of medium, resulting in the required final drug concentrations. Following drug addition, the plates are incubated for an additional 48 h at 37 ^{o}C, 5% CO_2, 95% air, and 100% relative humidity. For adherent cells, the assay is terminated by the addition of cold TCA. Cells are fixed in situ by the gentle addition of 50 µL of cold 50% (w/v) TCA (final concentration, 10% TCA) and incubated for 60 min at 4 ^{o}C. The supernatant is discarded, and the plates are washed five times with tap water and air dried. Sulforhodamine B (SRB) solution (100 mL) at 0.4% (w/v) in 1% acetic acid is added to each well, and plates are incubated for 10 min at room temperature. After staining, unbound dye is removed by washing five times with 1% acetic acid and the plates are air dried. Bound stain

is subsequently solubilized with 10 mM trizma base, and the absorbance is read on an automated plate reader at a wavelength of 515 nm. For suspension cells, the methodology is the same except that the assay is terminated by fixing settled cells at the bottom of the wells by gently adding 50 µl of 80% TCA (final concentration, 16% TCA). Using the seven absorbance measurements [time zero, (Tz), control growth, (C), and test growth in the presence of drug at the five concentration levels (Ti)], the percentage growth is calculated at each of the drug concentrations levels.

Percentage growth inhibition is calculated as: [(Ti Tz)/(C–Tz)] x 100 for concentrations for which Ti>/=Tz and [(Ti-Tz)/Tz] x 100 for concentrations for which Ti < Tz.

Three dose response parameters are calculated for each experimental agent. Growth inhibition of 50% (GI_{50}) is calculated from [(Ti -Tz)/(C–Tz)] x 100 = 50, which is the drug concentration resulting in a 50% reduction in the net protein increase (as measured by SRB staining) in control cells during the drug incubation. The drug concentration resulting in total growth inhibition (TGI) is calculated from Ti = Tz. The LC_{50} (concentration of drug resulting in a 50% reduction in the measured protein at the end of the drug treatment as compared to that at the beginning) indicating a net loss of cells following treatment is calculated from [(Ti–Tz)/Tz] x 100 = -50. Values are calculated for each of these three parameters if the level of activity is reached; however, if the effect is not reached or is exceeded, the value for that parameter is expressed as greater or less than the maximum or minimum concentration tested. Results for each compound were reported as a mean graph of the percent growth of the treated cells when compared to the untreated control cells. There after obtaining the results for one dose assay, analysis of historical Development Therapeutics Programme (DTP) was performed and compounds which satisfies predetermined threshold inhibition criteria is selected for NCI full panel 5 dose assay.

4. One dose mean graphs of the oxindole-benzothiazoles

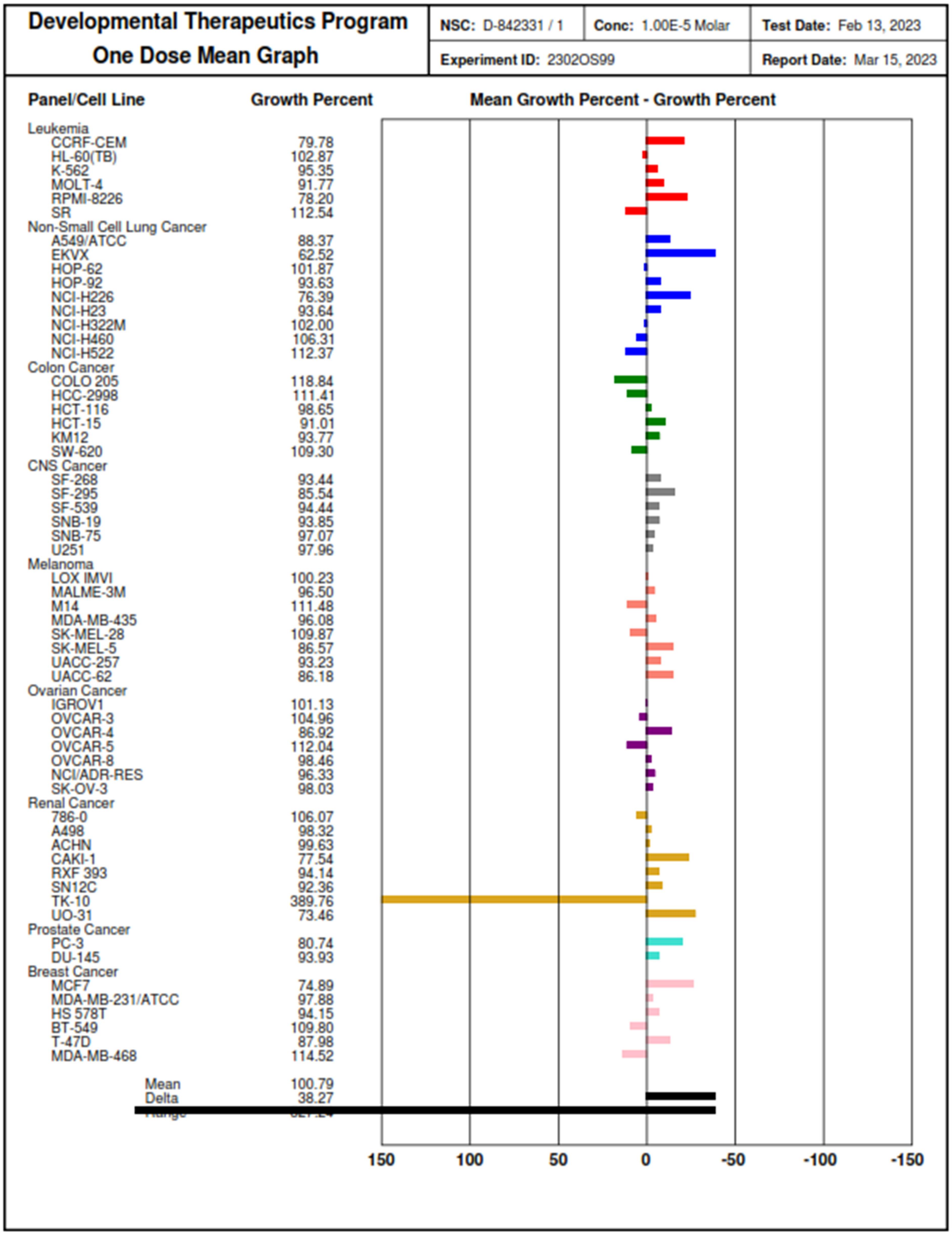

Figure 47. One dose mean graph of 9a

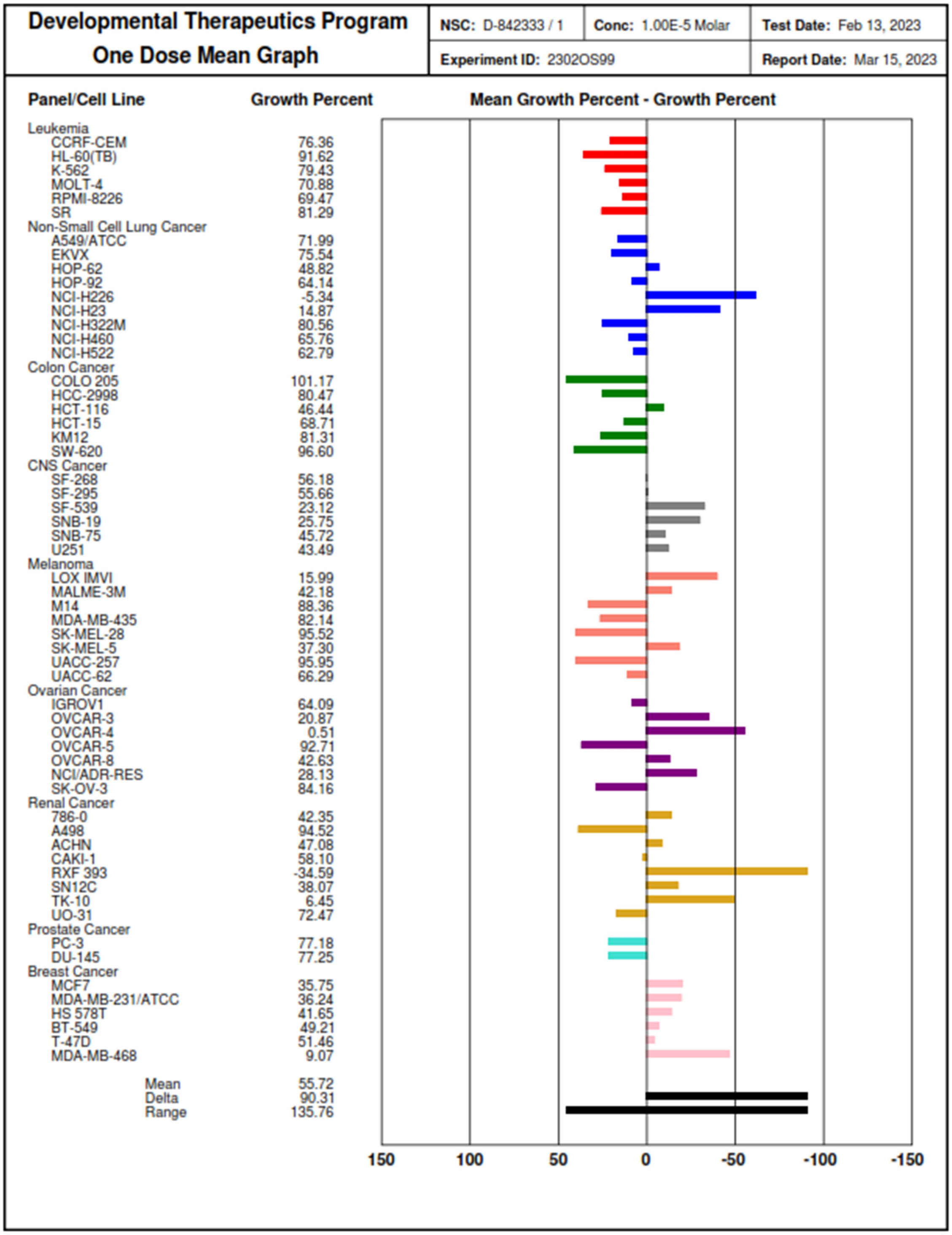

Figure 48. One dose mean graph of 9b

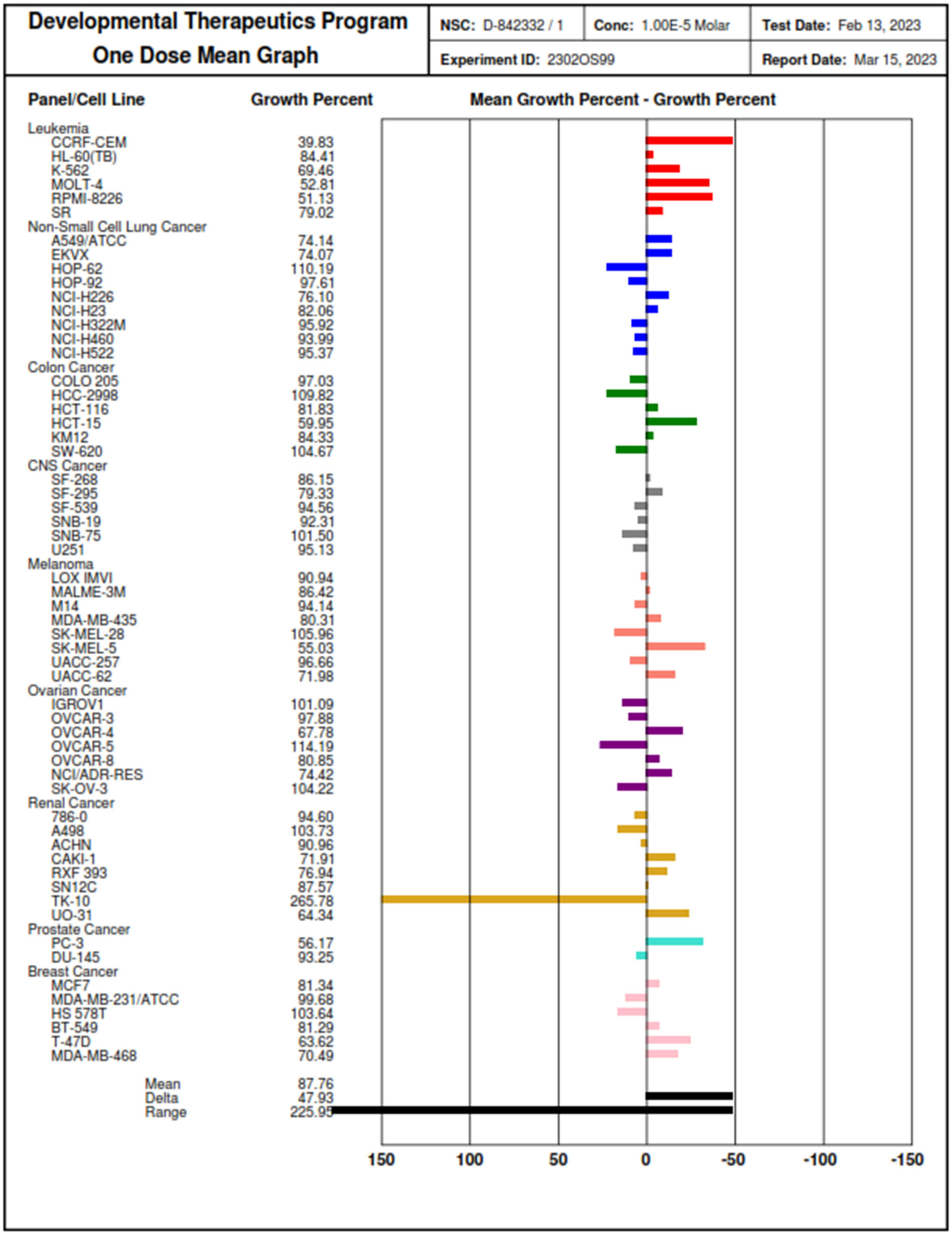

Figure 49. One dose mean graph of 9c

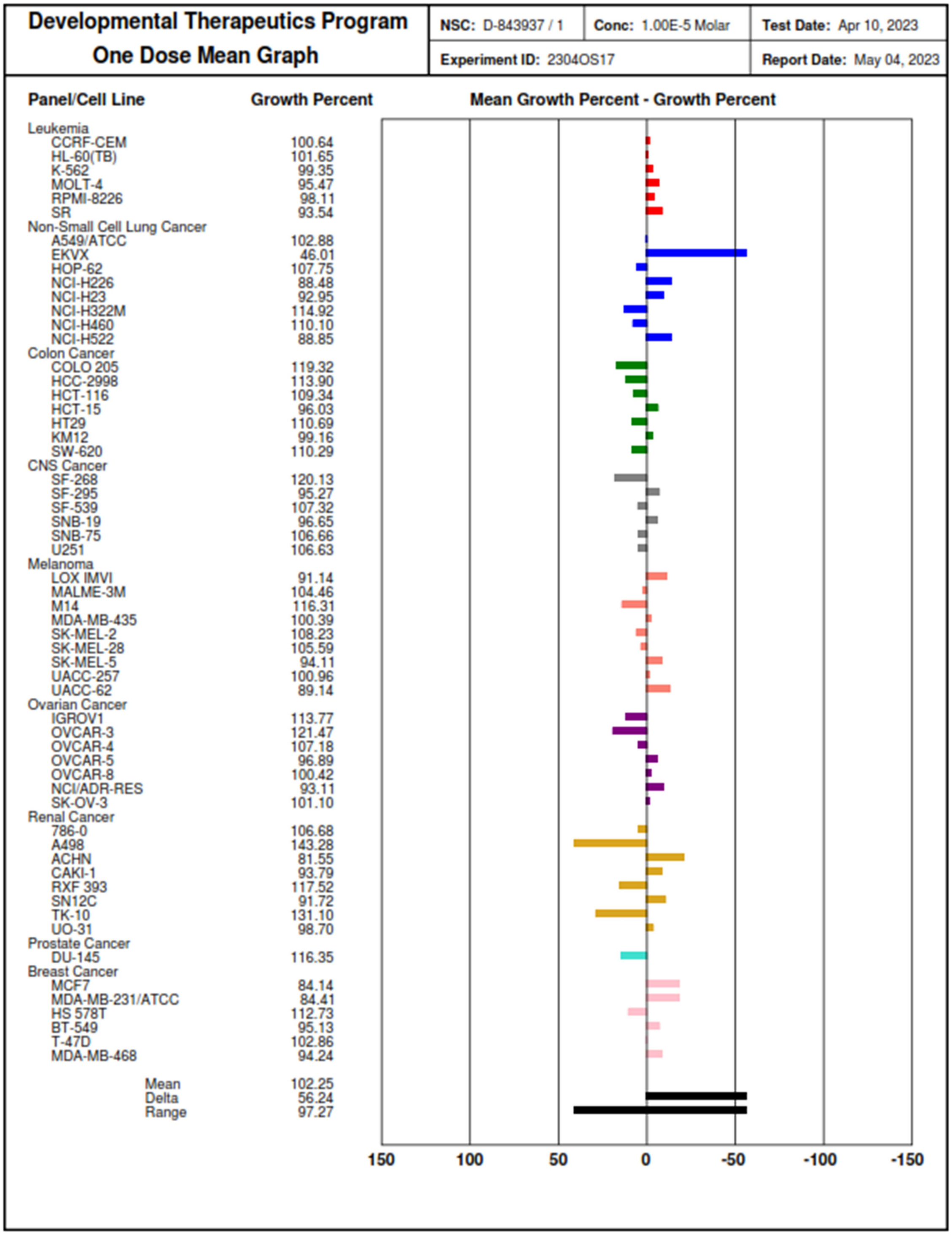

Figure 50. One dose mean graph of 9e

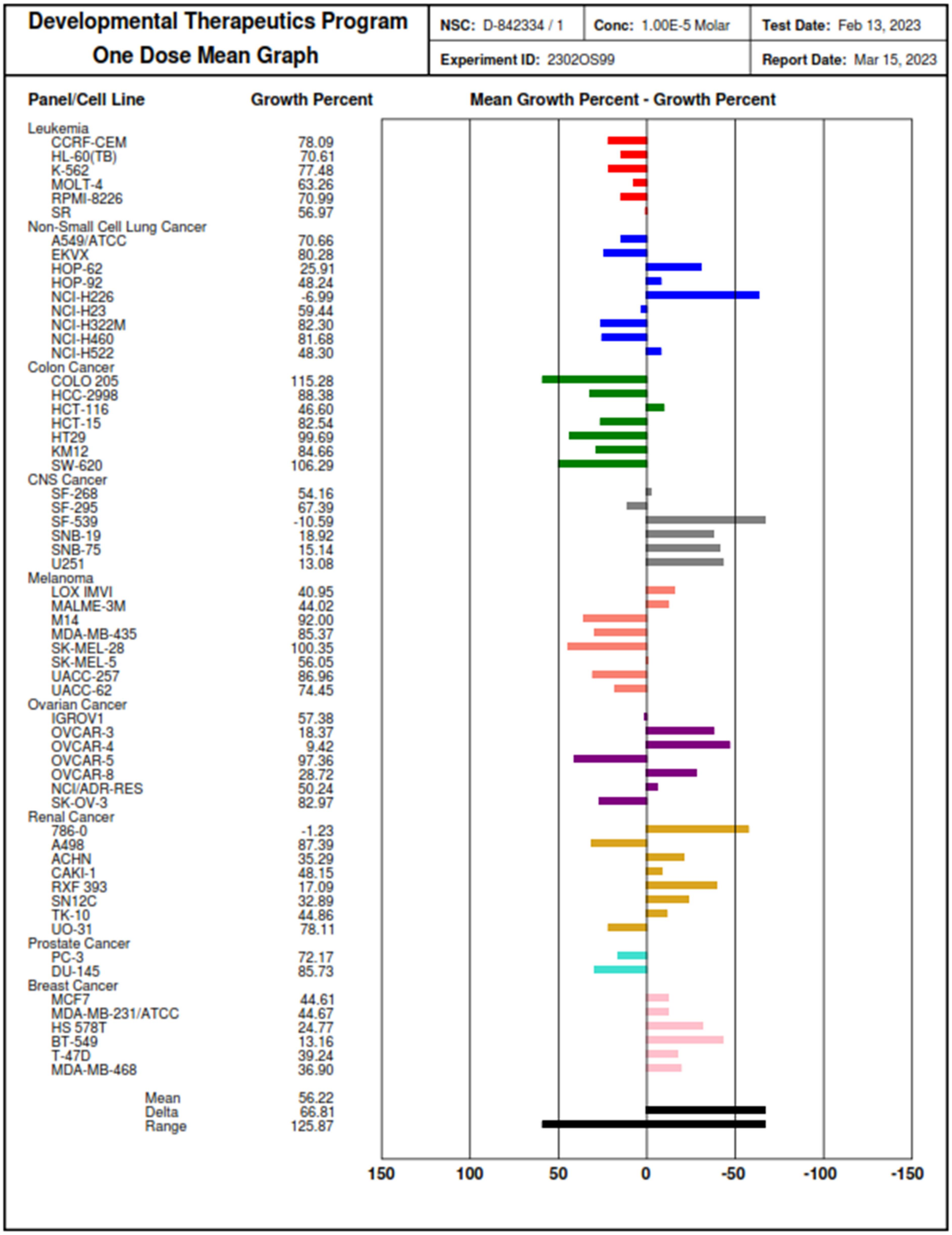

Figure 51. One dose mean graph of 9f

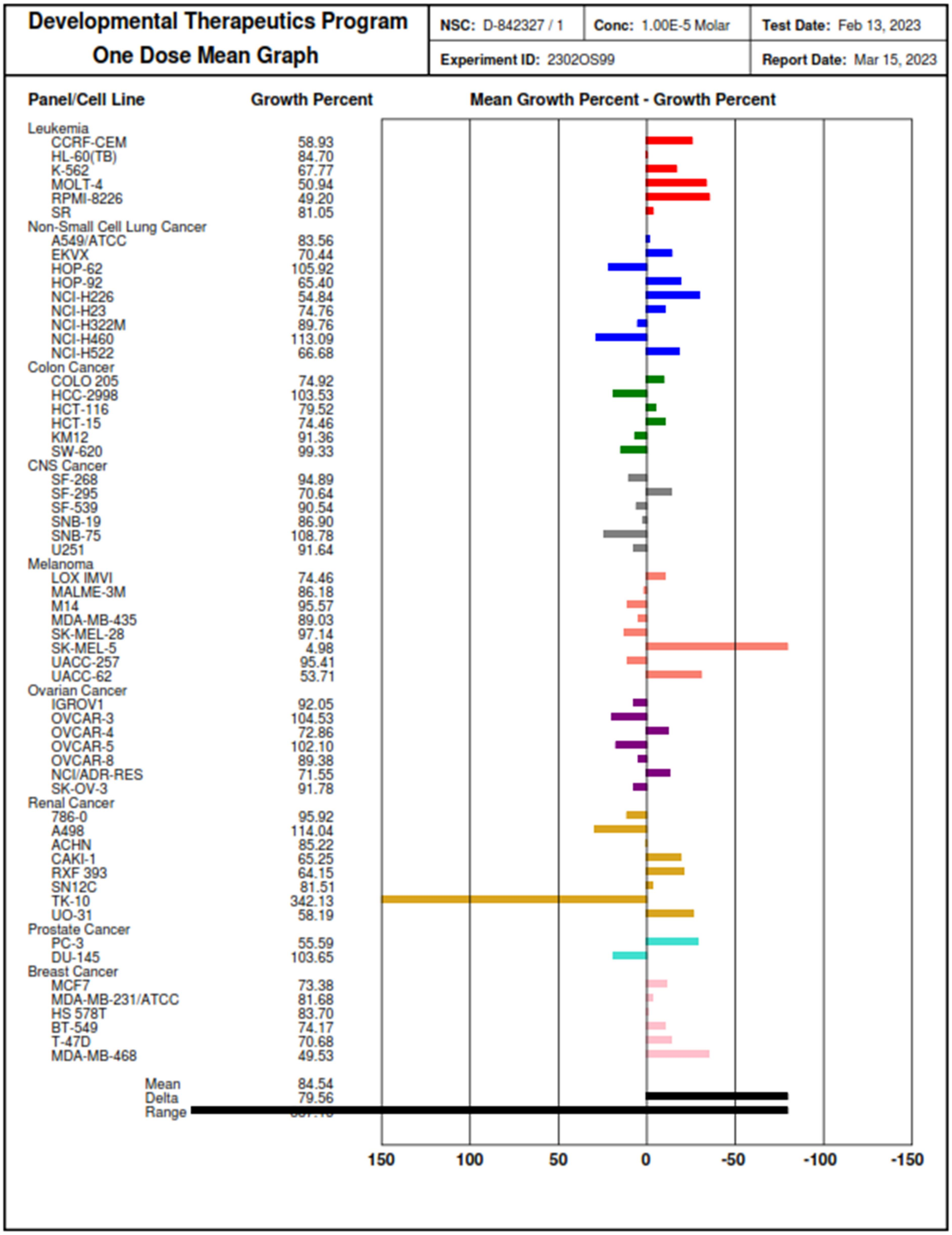

Figure 52. One dose mean graph of 9g

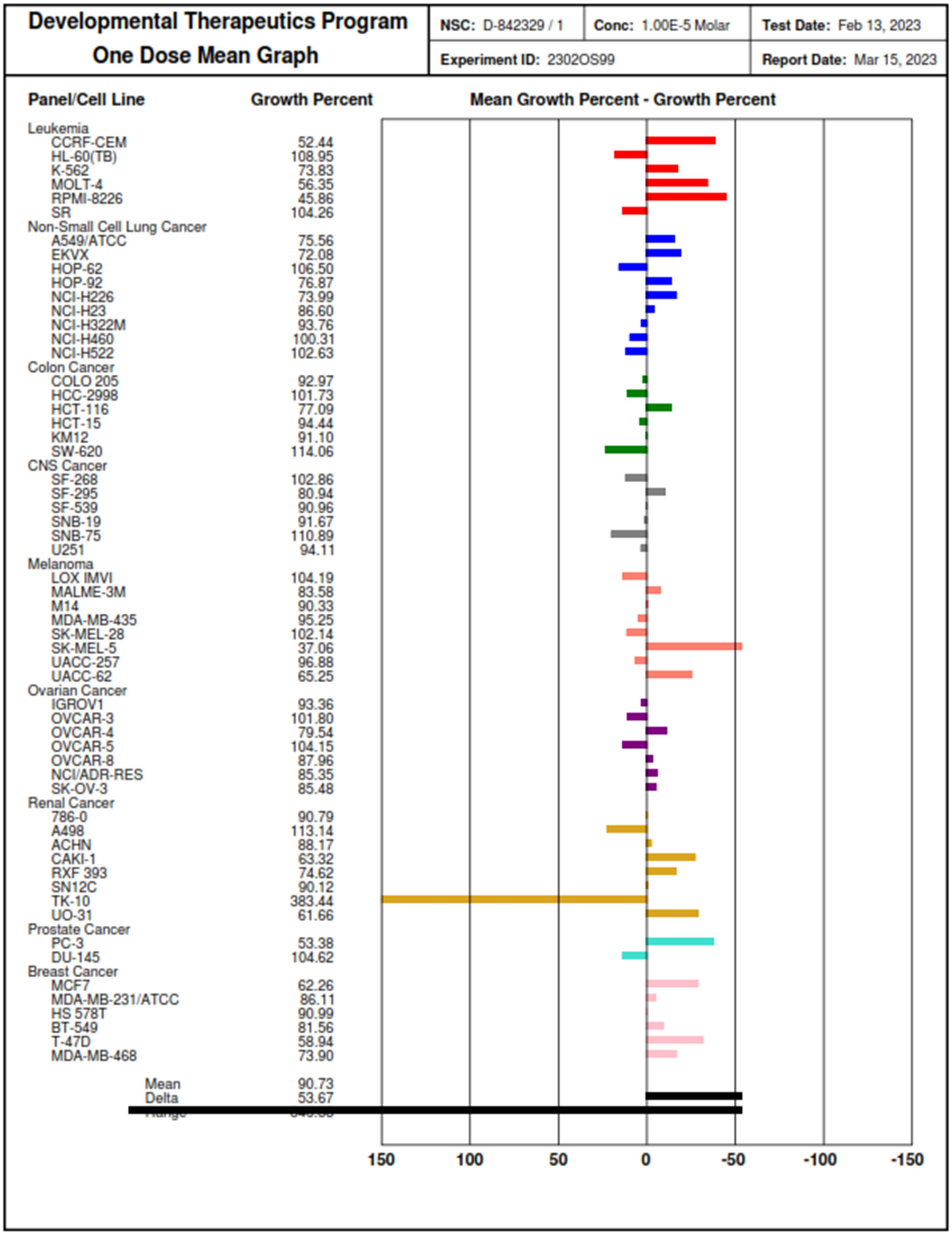

Figure 53. One dose mean graph of 9h

Developmental Therapeutics Program One Dose Mean Graph	NSC: D-842328 / 1	Conc: 1.00E-5 Molar	Test Date: Feb 13, 2023
	Experiment ID: 2302OS99		Report Date: Mar 15, 2023

Panel/Cell Line	Growth Percent
**Leukemia**	
CCRF-CEM	55.76
HL-60(TB)	99.42
K-562	78.05
MOLT-4	54.70
RPMI-8226	52.32
SR	103.25
**Non-Small Cell Lung Cancer**	
A549/ATCC	81.74
EKVX	75.90
HOP-62	105.82
HOP-92	68.34
NCI-H226	70.96
NCI-H23	88.35
NCI-H322M	94.11
NCI-H460	107.33
NCI-H522	92.96
**Colon Cancer**	
COLO 205	97.84
HCC-2998	104.39
HCT-116	82.45
HCT-15	90.13
KM12	91.54
SW-620	117.37
**CNS Cancer**	
SF-268	101.95
SF-295	86.14
SF-539	93.53
SNB-19	88.42
SNB-75	105.47
U251	102.37
**Melanoma**	
LOX IMVI	97.83
MALME-3M	87.19
M14	92.08
MDA-MB-435	95.68
SK-MEL-28	100.76
SK-MEL-5	48.18
UACC-257	102.41
UACC-62	74.67
**Ovarian Cancer**	
IGROV1	99.85
OVCAR-3	107.31
OVCAR-4	79.86
OVCAR-5	104.04
OVCAR-8	88.41
NCI/ADR-RES	89.42
SK-OV-3	90.02
**Renal Cancer**	
786-0	94.38
A498	115.13
ACHN	93.49
CAKI-1	75.02
RXF 393	84.59
SN12C	85.34
TK-10	427.16
UO-31	69.71
**Prostate Cancer**	
PC-3	56.38
DU-145	105.70
**Breast Cancer**	
MCF7	76.88
MDA-MB-231/ATCC	89.70
HS 578T	95.85
BT-549	83.90
T-47D	62.43
MDA-MB-468	69.34
Mean	93.68
Delta	45.50
[illegible]	[illegible]

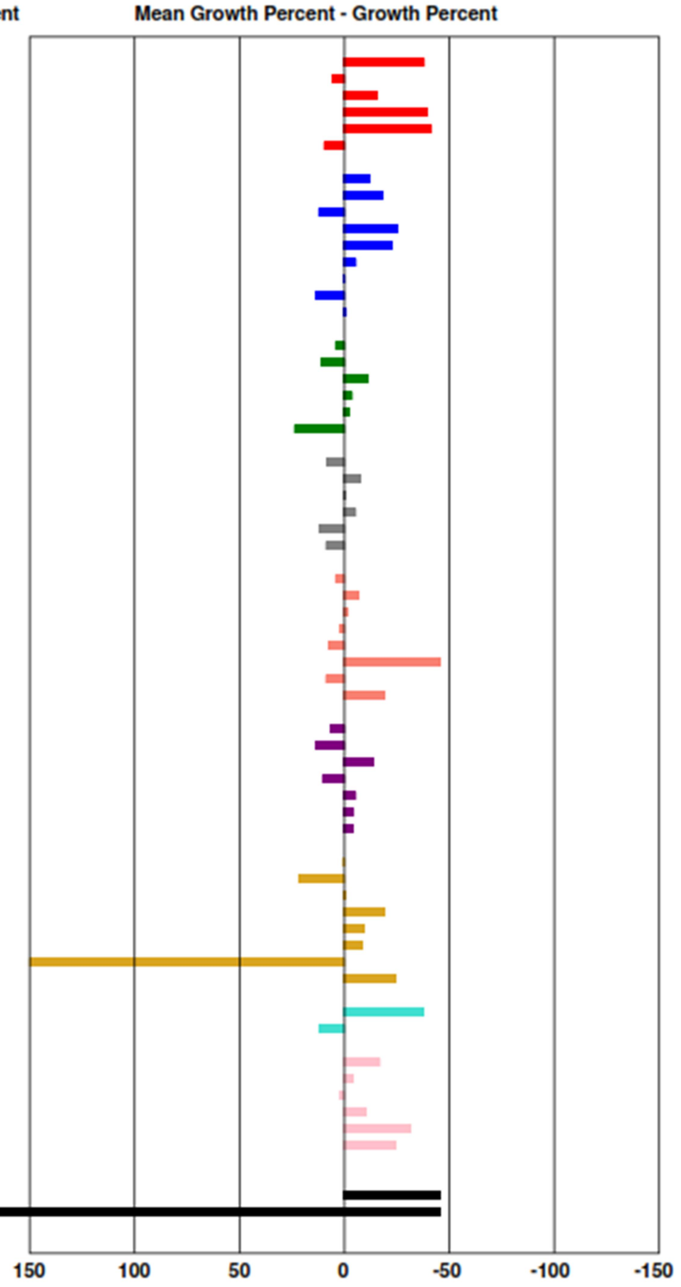

Figure 54. One dose mean graph of 9i

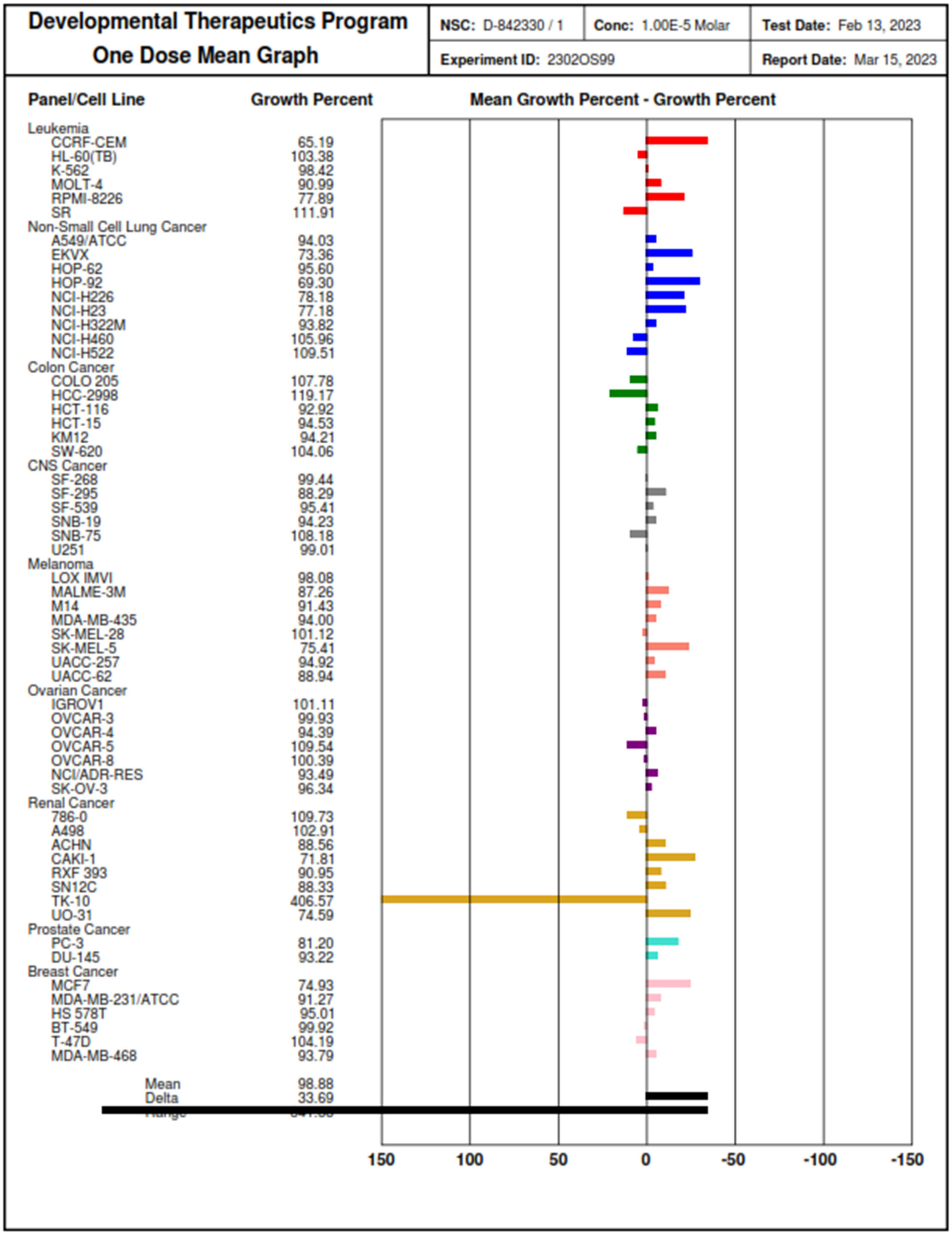

Figure 55. One dose mean graph of 9j

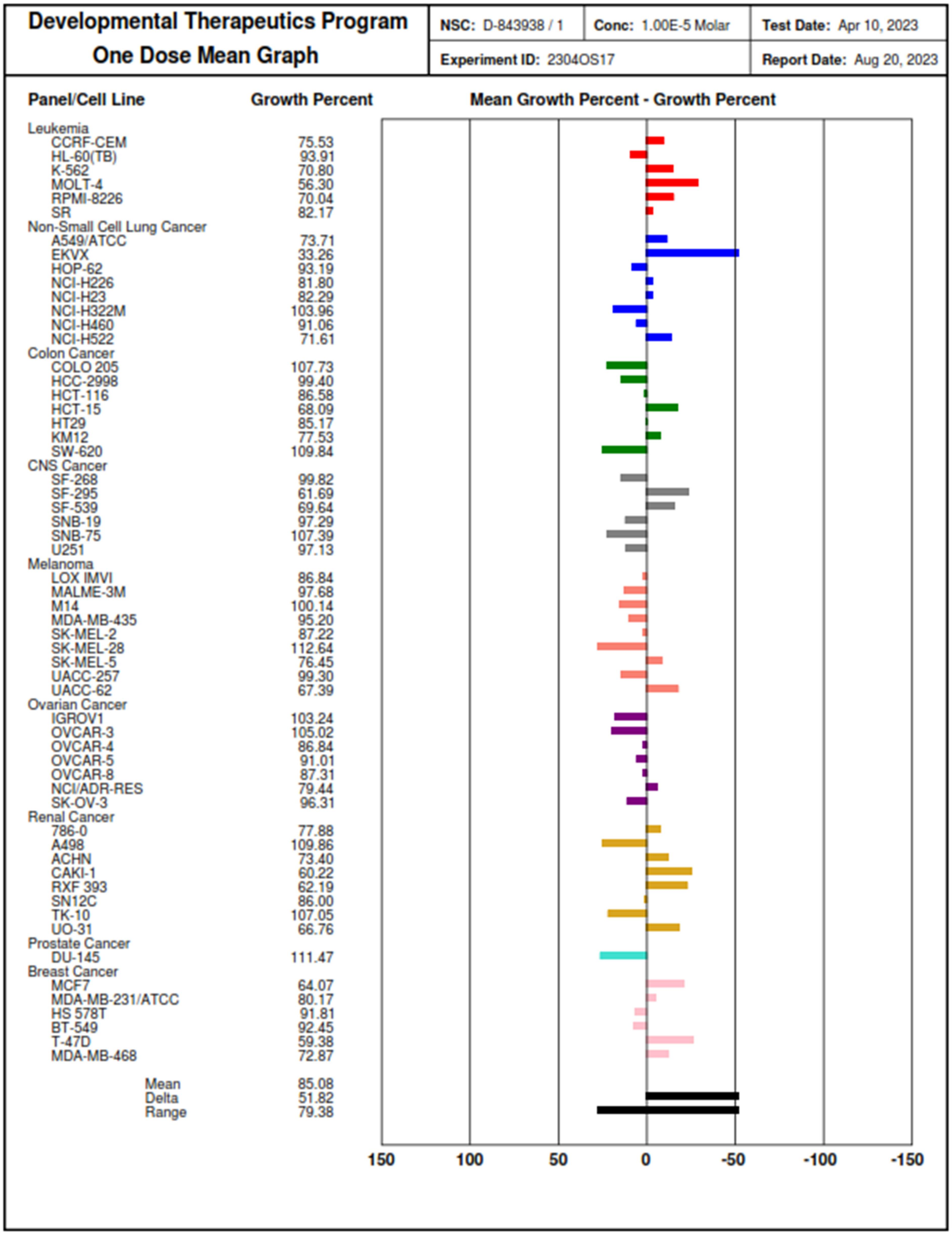

Figure 56. One dose mean graph of 9k

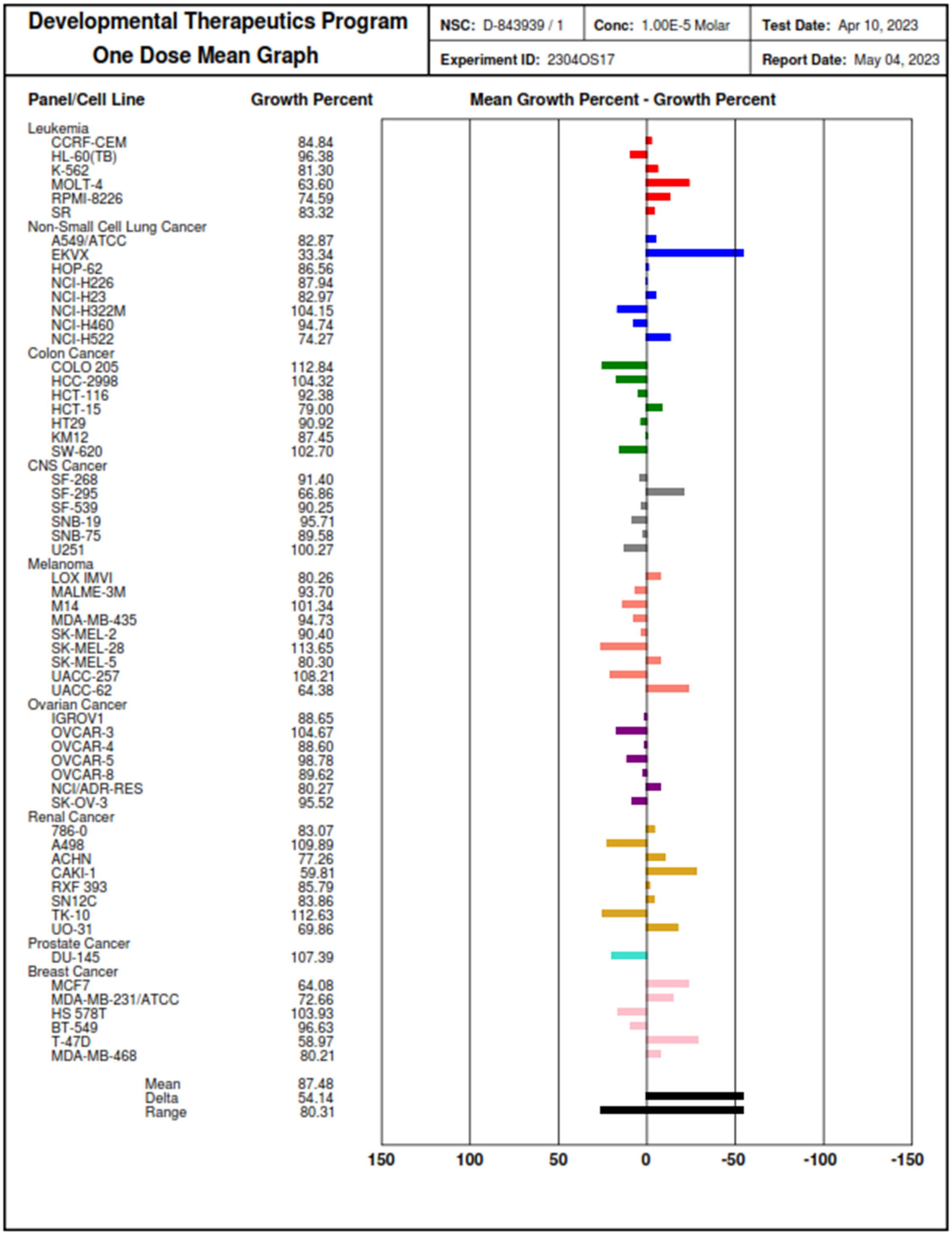

Figure 57. One dose mean graph of 9l

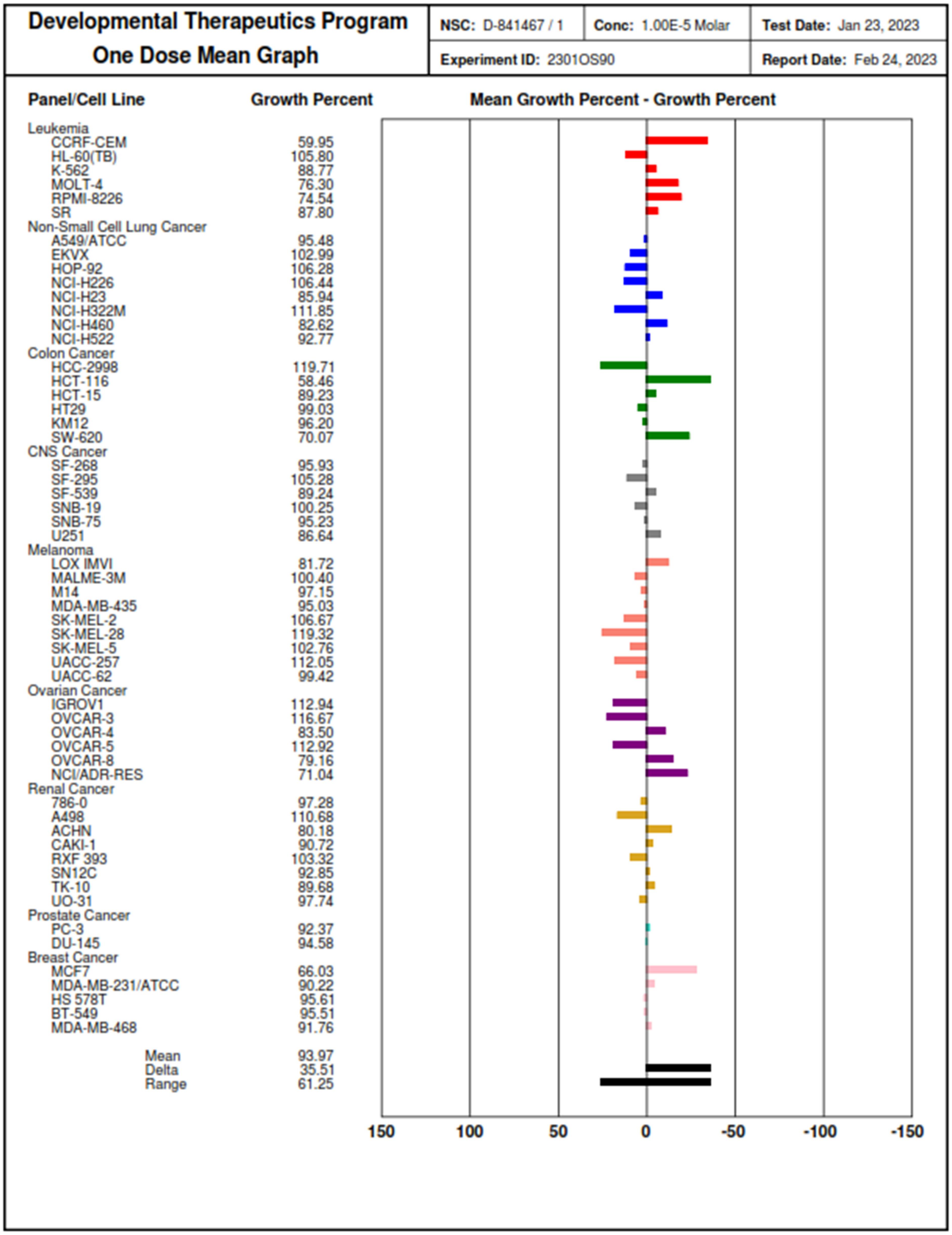

Figure 58. One dose mean graph of 9m

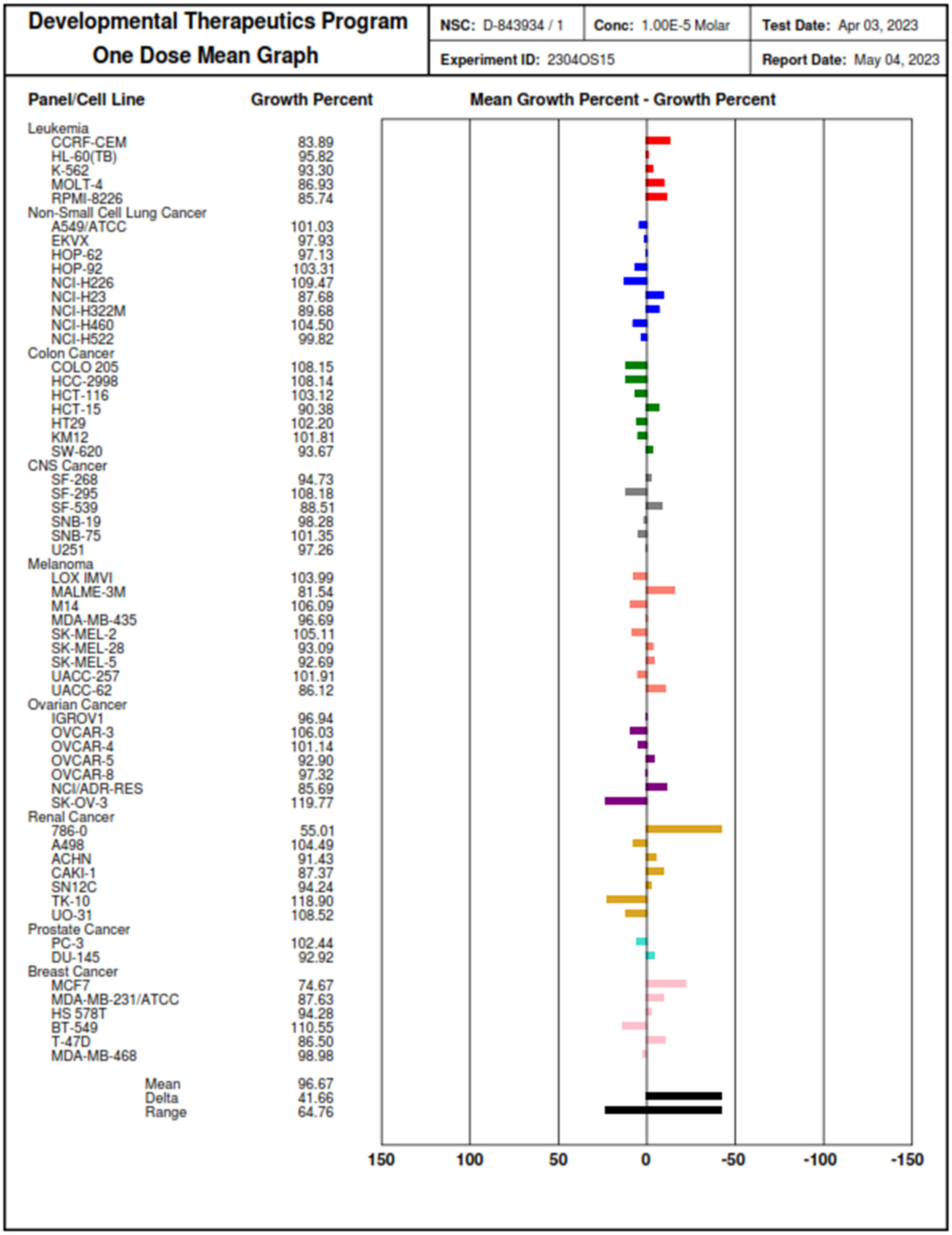

Figure 59. One dose mean graph of 9n

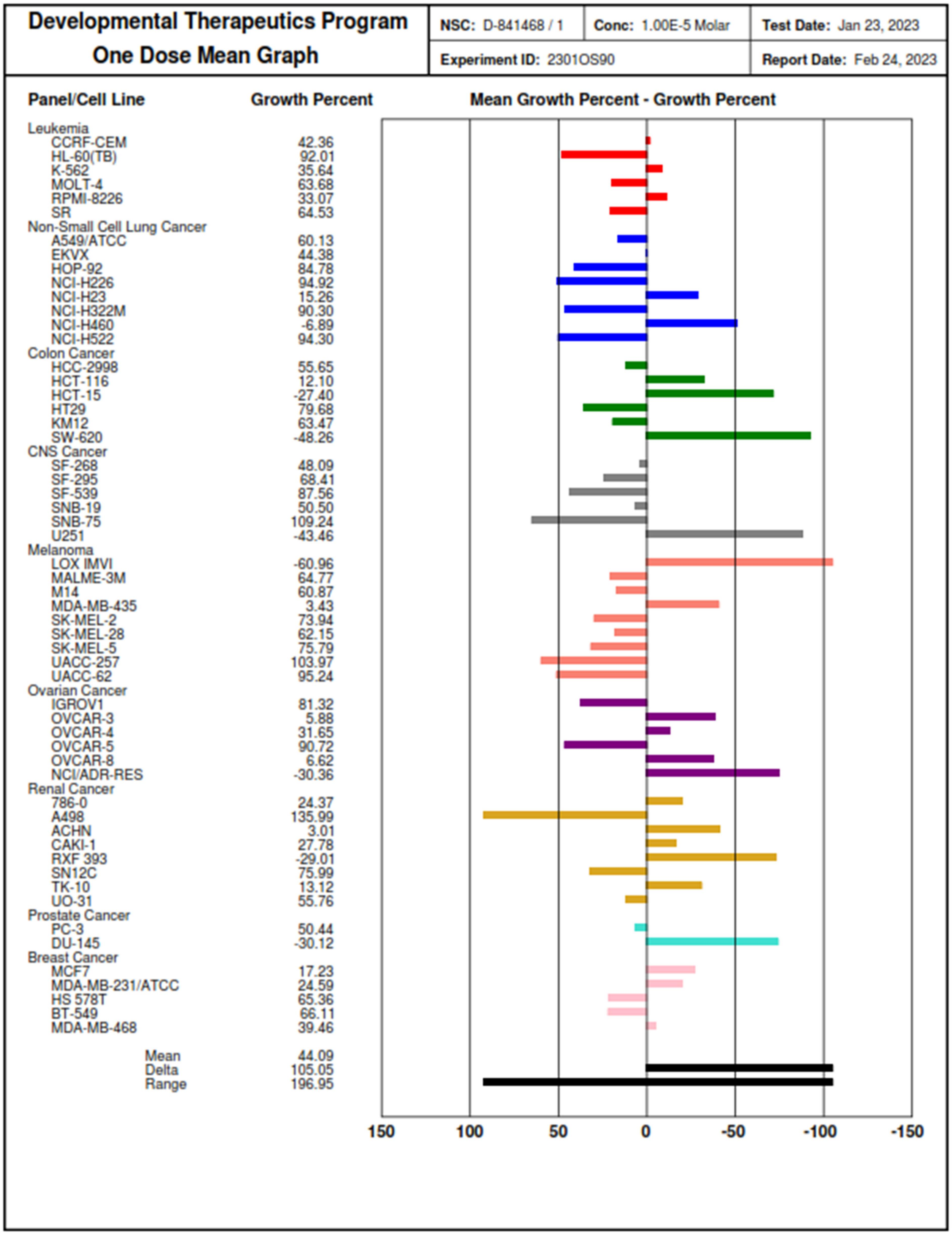

Figure 60. One dose mean graph of 9o

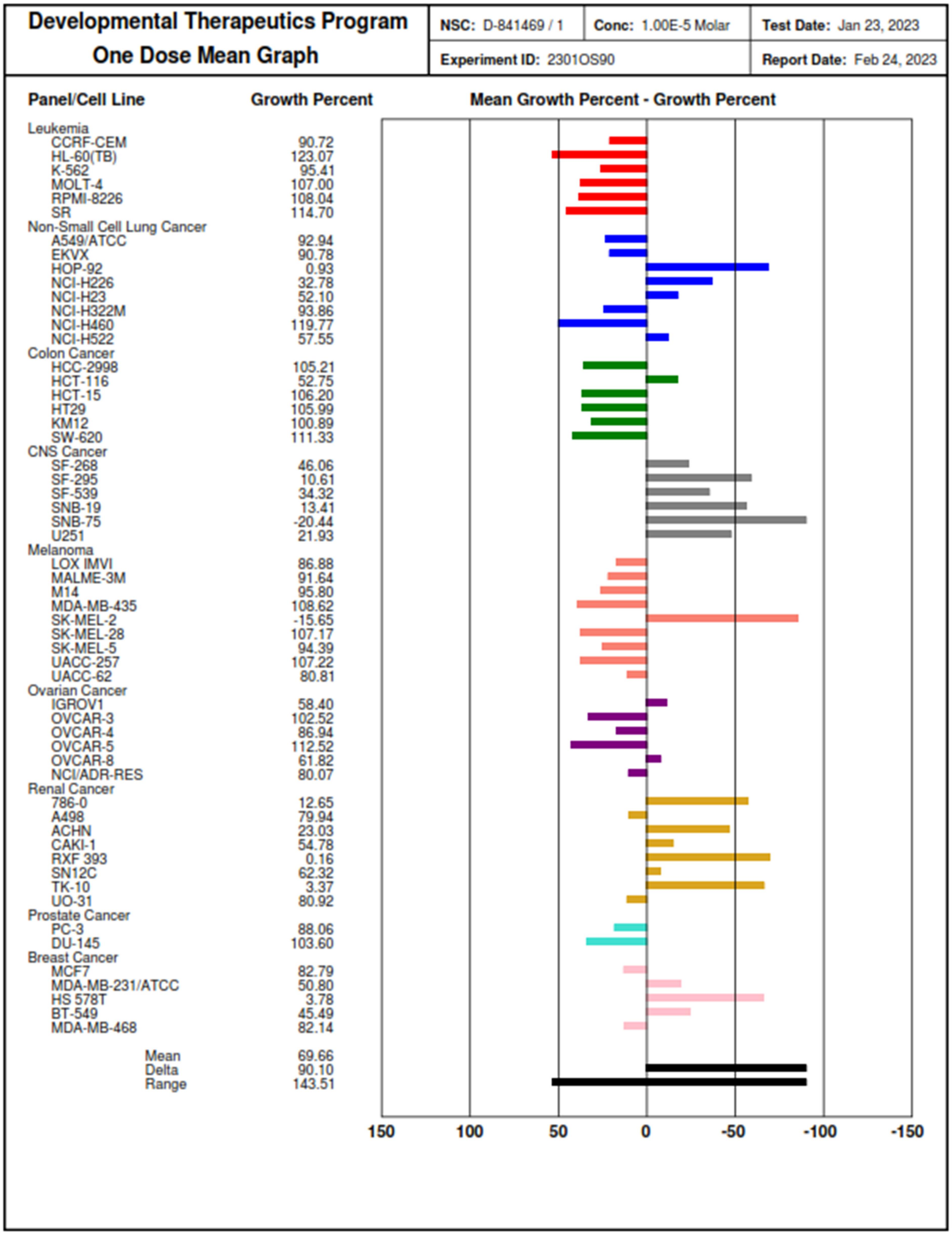

Figure 61. One dose mean graph of 9p

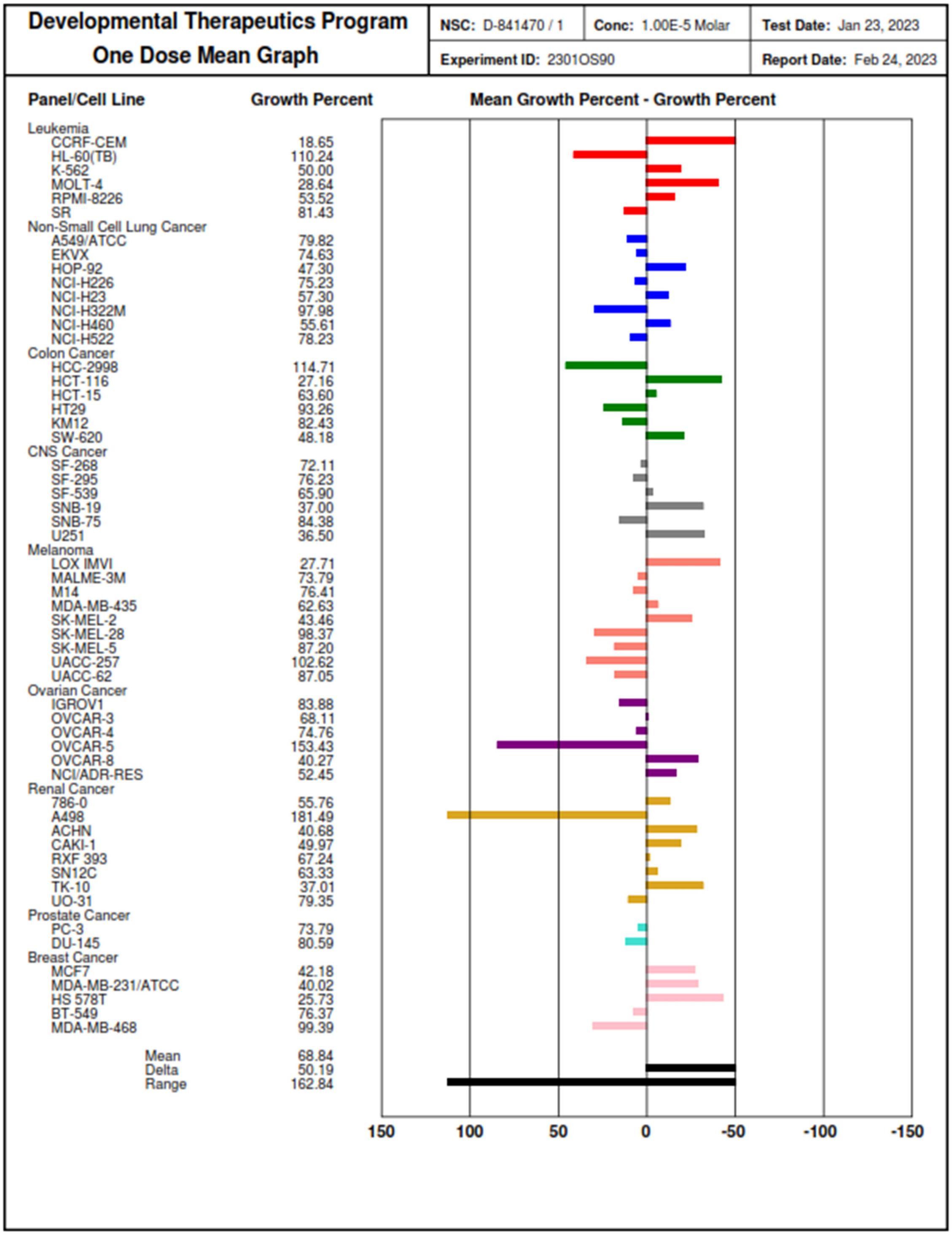

Figure 62. One dose mean graph of 9q

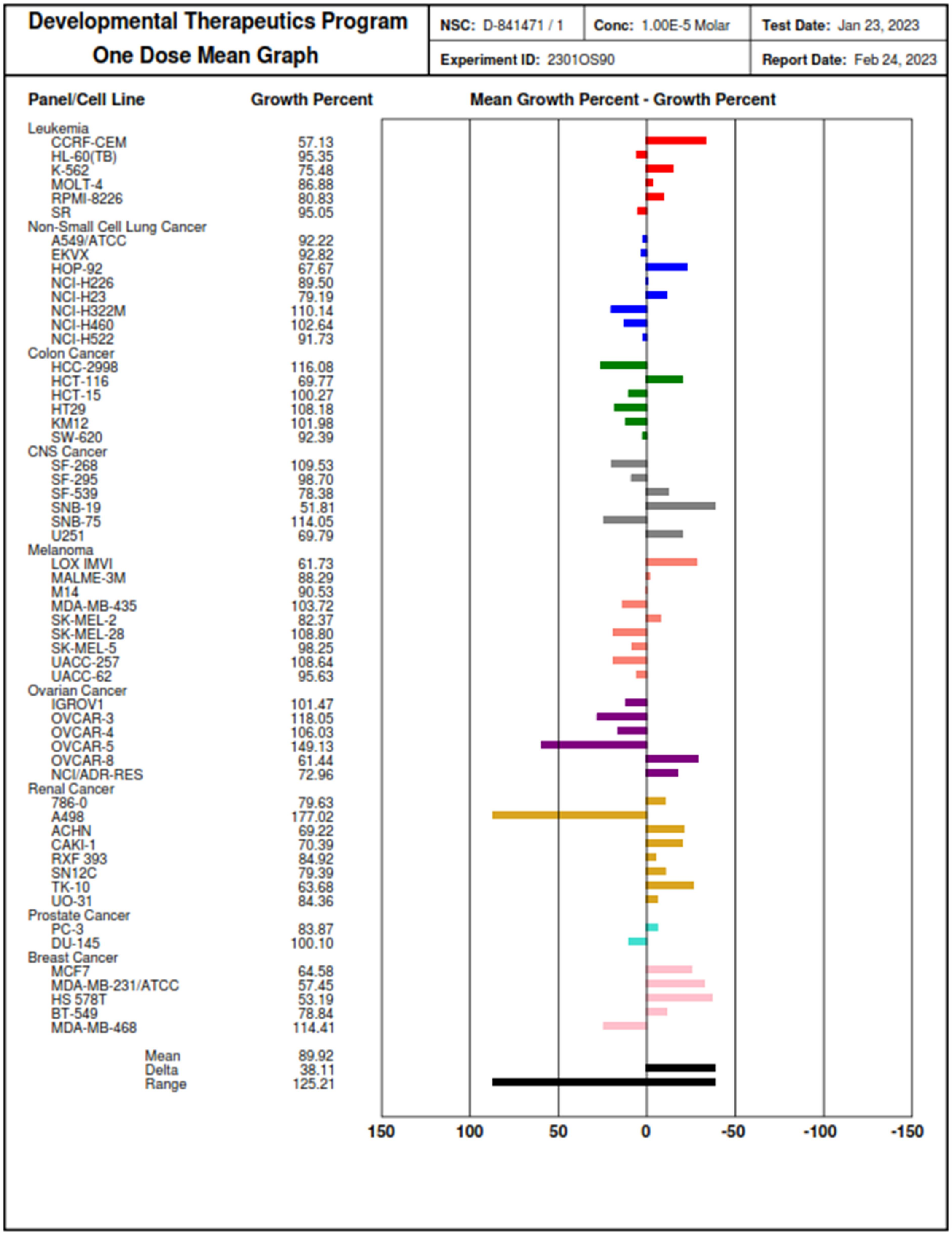

Figure 63. One dose mean graph of 9r

5. Dose response curve of 9o on NCI cancer cell lines

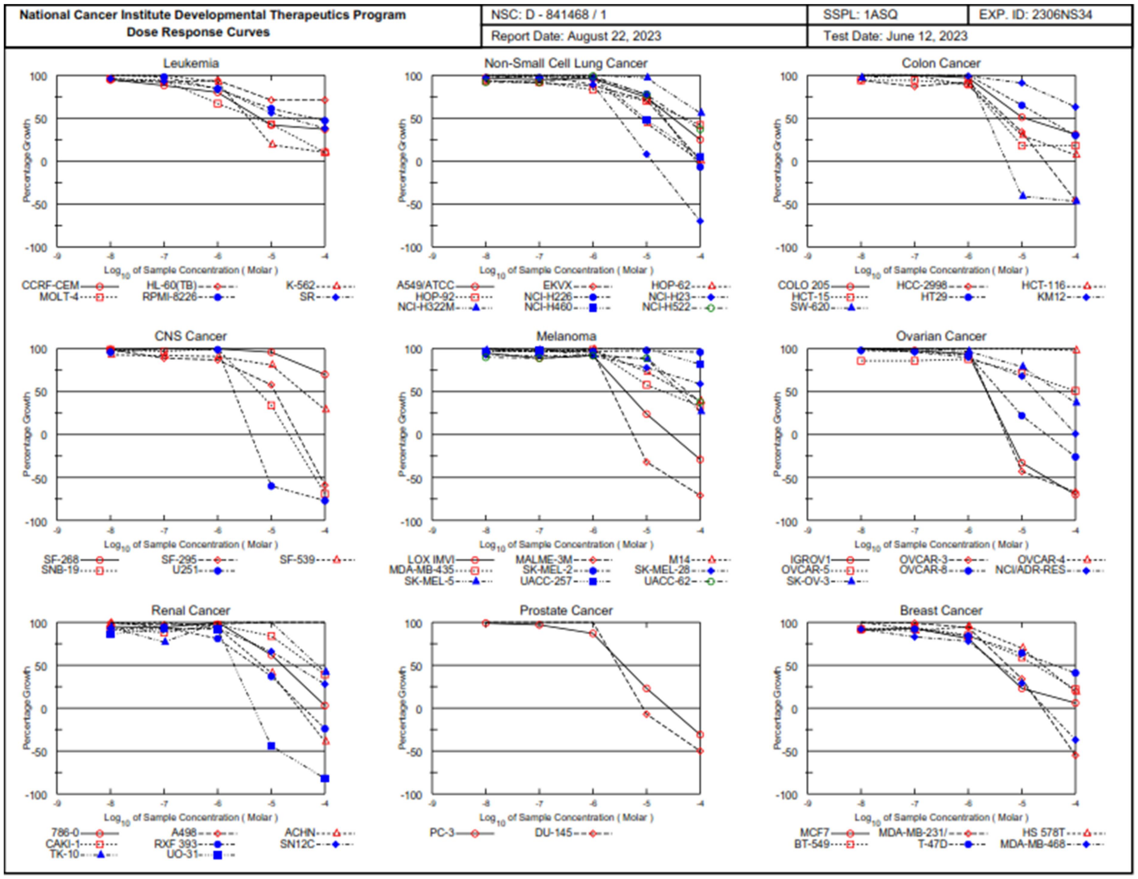

Figure 64. Dose response curves of **9o** on NCI cancer cell lines

6. Analysis of cell cycle distribution

DU-145: Prostate cancer was obtained from Nawah Scientific Inc., (Mokatam, Cairo, Egypt). Cells were maintained in DMEM media supplemented with 100 mg/mL of streptomycin, 100 units/mL of penicillin and 10% of heat-inactivated fetal bovine serum in humidified, 5% (v/v) CO_2 atmosphere at 37 °C. After treatment with **9o** for 48h, cells (105 cells) are collected by trypsinization and washed twice with ice-cold PBS (pH 7.4). Cells are re-suspended in two milliliters of 60% ice-cold ethanol and incubated at 4ºC for 1h for fixation. Fixed cells are washed twice again with PBS (pH 7.4) and re-suspended in 1 mL of PBS containing 50 µg/mL RNAase A and 10 µg/mL propidium iodide (PI). After 20 min of incubation in dark at 37 ^{o}C, cells are analyzed for DNA contents using flow cytometry analysis using FL2 (λex/em 535/617 nm) signal detector (ACEA Novocyte™ flowcytometer, ACEA Biosciences Inc., San Diego, CA, USA). For each sample, 12,000 events are acquired. Cell cycle distribution is calculated using ACEA NovoExpress™ software (ACEA Biosciences Inc., San Diego, CA, USA).

7. Apoptosis assay

Apoptosis and necrosis cell populations are determined using Annexin V-FITC apoptosis detection kit (Abcam Inc., Cambridge Science Park, Cambridge, UK) coupled with 2 fluorescent channels flowcytometry. After treatment with test compounds for 48h, cells (105 cells) are collected by trypsinization and washed twice with ice-cold PBS (pH 7.4). Then, cells are incubated in dark with 0.5 ml of Annexin V-FITC/PI solution for 30 min in dark at room temperature according to manufacturer protocol. After staining, cells are injected via ACEA Novocyte™ flowcytometer (ACEA Biosciences Inc., San Diego, CA, USA) and analysed for FITC and PI fluorescent signals using FL1 and FL2 signal detector, respectively (λex/em 488/530 nm for FITC and λex/em 535/617 nm for PI). For each sample, 12,000 events are acquired and positive FITC and/or PI cells are quantified by quadrant analysis and calculated using ACEA NovoExpress™ software (ACEA Biosciences Inc., San Diego, CA, USA).

8. Biochemical kinase assay procedure

The inhibitory activity against CDK2 was determined using CDK2 kinase kit (cat. ID: 79599), purchased from BPS Biosciences and kinase-Glo Max luminescence kinase assay kit (Promega). Sorafenib was used as a reference multi-kinases inhibitor.

The assay was carried out according to the protocol provided by the manufacturer. A stock solution of the synthesized derivatives in 100% DMSO was prepared. Subsequently, the compounds were diluted to 10% DMSO.

A master mixture was prepared according to the number of wells. For CDK2 assay each well include 6 μl of 5x Kinase Buffer 1 + 1 μl of ATP (500 μM) + 5 μl of 10X CDK substrate peptide 1 + 13 μl of distilled water. 1x Kinase buffer 1 was prepared by mixing 600 μl of 5x Kinase Buffer 1 with 2400 μl water to give 3 mL. Kinases were thawed on ice and were diluted with 1x Kinase Buffer 1.

To start the biochemical reaction, 25 μL of the master mixture was added to each well in 96 well plate. 5 μL of 10% DMSO was added to positive control wells and blank wells. 5 μL of diluted inhibitor was added to each well labelled with the test inhibitor so that the final concentration of DMSO is 1% in all reactions. Then 20 μL of CDK2/CyclinA2 (2.5 ng/μl) in 1x kinase buffer was added to positive control wells and wells labelled with the inhibitor, while 20 μL 1x kinase buffer 1 was added to the blank wells.

The plate was incubated at 30 ^{o}C for 45 min. Subsequently, 50 μl of Kinase-Glo Max luminescence reagent was added to each well and the plate was covered with aluminum foil and incubated at room temperature for 15 min. Finally, the luminescence was recorded using multimode microplate reader. Kinase activity assays were performed in duplicate at each concentration.

The luminescence data were analyzed as follows. The difference between luminescence intensities in the absence of kinase (Lu_t) and in the presence of kinase (Lu_c) was defined as 100 % activity ($Lu_t – Lu_c$). Using luminescence signal (Lu) in the presence of the compound, % activity was calculated as: % activity = $\{(Lu_t - Lu)/(Lu_t - Lu_c)\} \times 100\%$. The concentration of the test compounds required to reduce the kinase activity by 50% was determined from dose-response curves and recorded as their IC_{50}.

9. Docking of the co-crystalized ligand in the binding site of CDK2

Docking experiments were performed by Autodock Vina software [1]. Briefly, the crystal structure of CDK2 (PDB ID: 1FVT) [2] was downloaded from the protein data bank. Initially the protein was prepared for the intended docking study by removal of water molecules followed by protonation. Subsequently, the receptor was saved in pdbqt format. The co-crystalized ligand and **9o** were also saved in pdbqt format. Self-docking of the co-crystalized ligand was performed to validate the docking protocol. After validation experiment (**Figure 65**), **9o** was docked in the binding site of CDK2 and the binding free energy was detected and the results were visualized by BIOVIA Discovery Studio Visualizer 2021 free visualizer (https://discover.3ds.com/discovery-studio-visualizer-download).

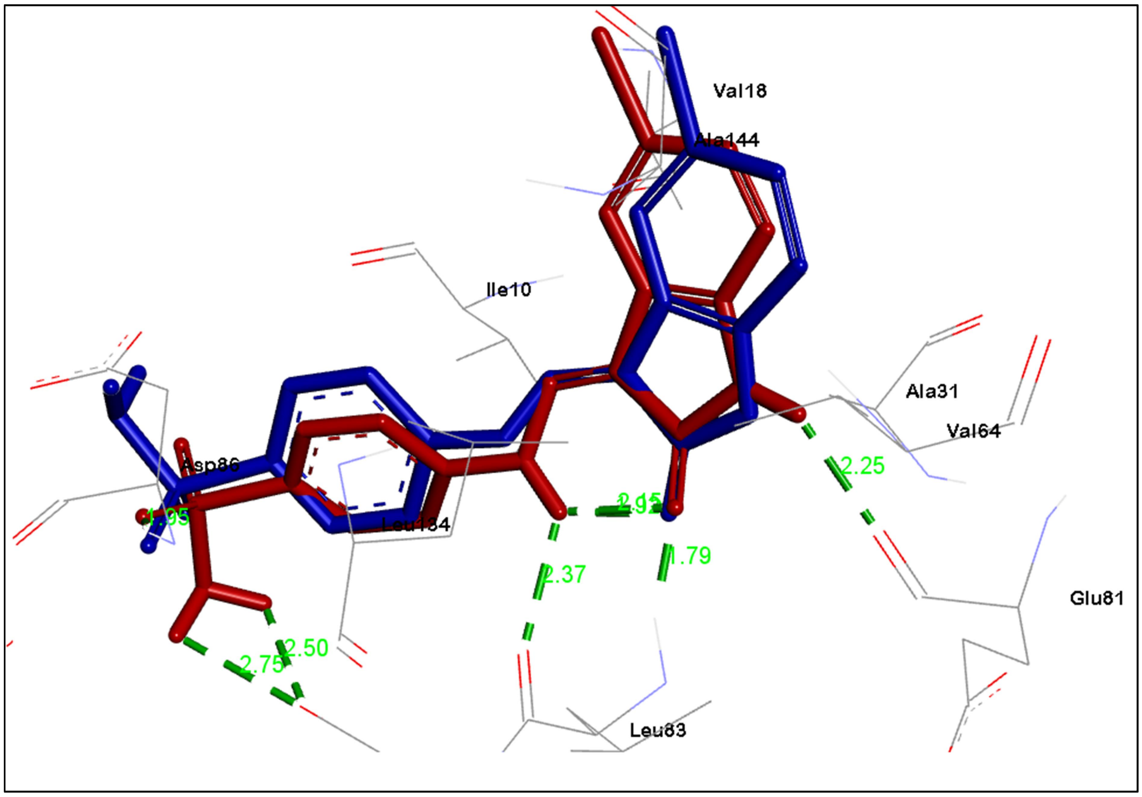

Figure 65. 3D diagram of the overlay of the docked pose (red) and the co-crystallized ligand (blue) in the CDK2 active site

10. Bioavailability Radar charts for 9a-r from Swiss ADME free webtool [3]

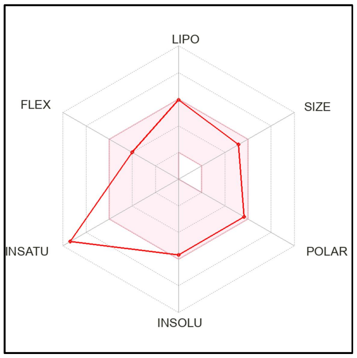

9a

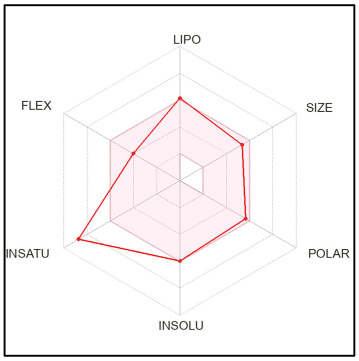

9b

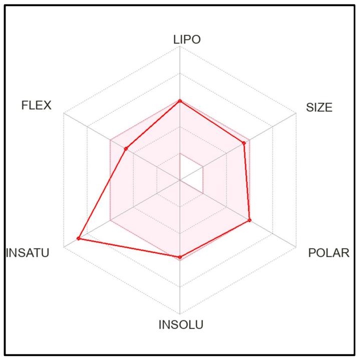

9c

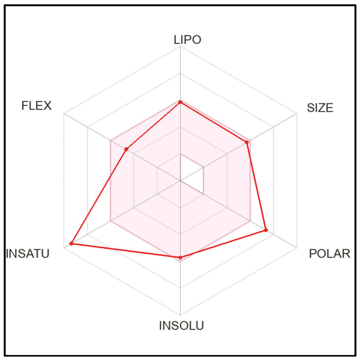

9d

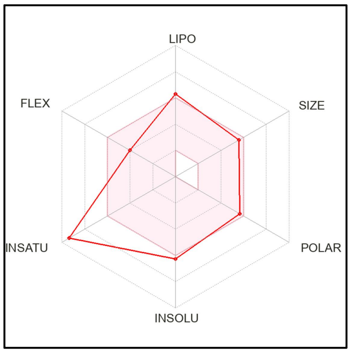

9e

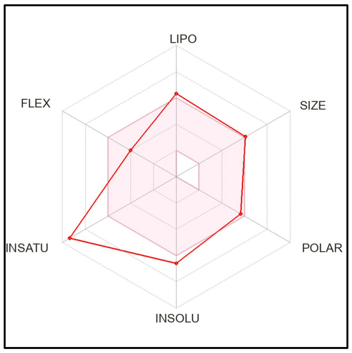

9f

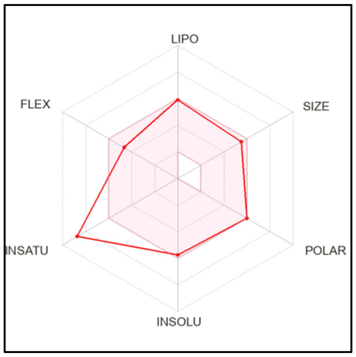

9g

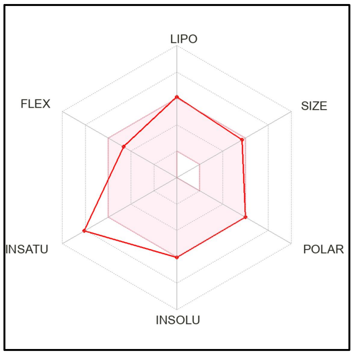

9h

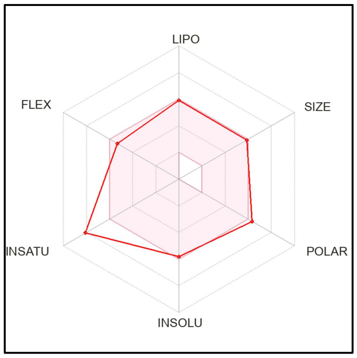

9i

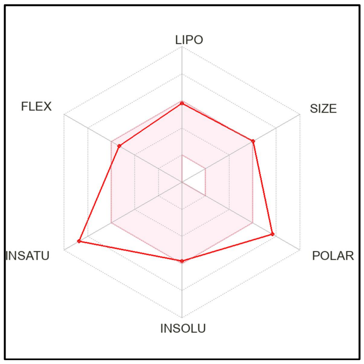

9j

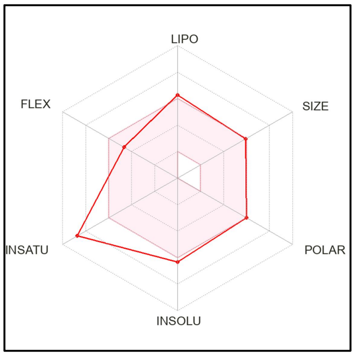

9k

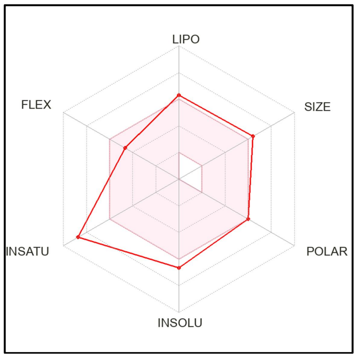

9l

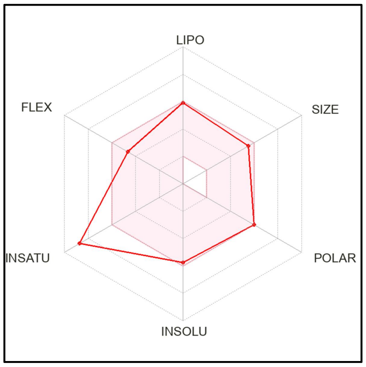

9m

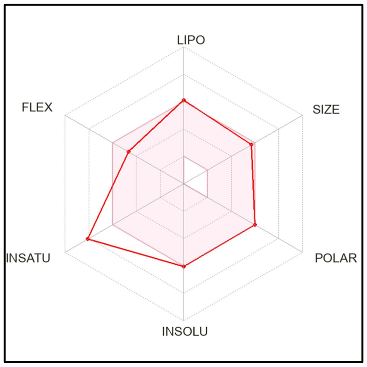

9n

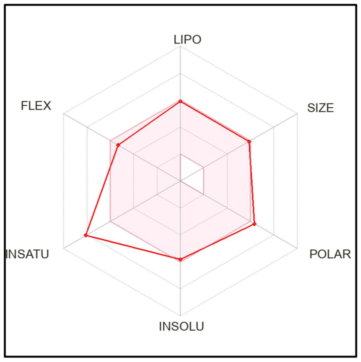

9o

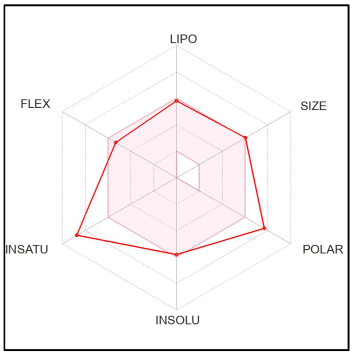

9p

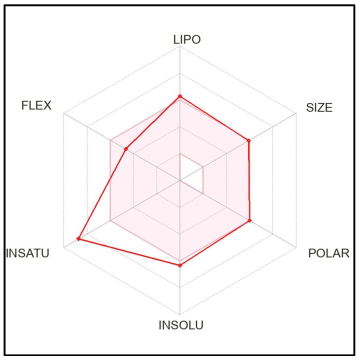

9q

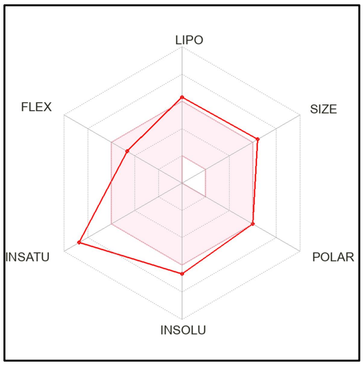

9r

Figure 66. Bioavailability Radar chart for **9a-r** from Swiss ADME free webtool

11. References

[1] O. Trott, A.J. Olson, AutoDock Vina: improving the speed and accuracy of docking with a new scoring function, efficient optimization, and multithreading, J Comput Chem 31 (2010) 455-61, 10.1002/jcc.21334.

[2] S.T. Davis, B.G. Benson, H.N. Bramson, D.E. Chapman, S.H. Dickerson, K.M. Dold, D.J. Eberwein, M. Edelstein, S.V. Frye, R.T. Gampe, Jr., R.J. Griffin, P.A. Harris, A.M. Hassell, W.D. Holmes, R.N. Hunter, V.B. Knick, K. Lackey, B. Lovejoy, M.J. Luzzio, D. Murray, P. Parker, W.J. Rocque, L. Shewchuk, J.M. Veal, D.H. Walker, L.F. Kuyper, Prevention of chemotherapy-induced alopecia in rats by CDK inhibitors, Science 291 (2001) 134-7, 10.1126/science.291.5501.134.

[3] A. Daina, O. Michielin, V. Zoete, SwissADME: a free web tool to evaluate pharmacokinetics, drug-likeness and medicinal chemistry friendliness of small molecules, Sci Rep 7 (2017) 42717, 10.1038/srep42717.
